# Comparative connectomics of the descending and ascending neurons of the *Drosophila* nervous system: stereotypy and sexual dimorphism

**DOI:** 10.1101/2024.06.04.596633

**Published:** 2024-06-06

**Authors:** Tomke Stürner, Paul Brooks, Laia Serratosa Capdevila, Billy J. Morris, Alexandre Javier, Siqi Fang, Marina Gkantia, Sebastian Cachero, Isabella R. Beckett, Andrew S. Champion, Ilina Moitra, Alana Richards, Finja Klemm, Leonie Kugel, Shigehiro Namiki, Han S.J. Cheong, Julie Kovalyak, Emily Tenshaw, Ruchi Parekh, Philipp Schlegel, Jasper S. Phelps, Brandon Mark, Sven Dorkenwald, Alexander S. Bates, Arie Matsliah, Szi-chieh Yu, Claire E. McKellar, Amy Sterling, Sebastian Seung, Mala Murthy, John Tuthill, Wei-Chung A. Lee, Gwyneth M. Card, Marta Costa, Gregory S.X.E. Jefferis, Katharina Eichler

**Affiliations:** 1 Neurobiology Division, MRC Laboratory of Molecular Biology, Cambridge, UK; 2 *Drosophila* Connectomics Group, Department of Zoology, University of Cambridge, Cambridge, UK; 3 Genetics Department, Leipzig University, Leipzig, Germany; 4 Research Center for Advanced Science and Technology, University of Tokyo, Tokyo, Japan; 5 Janelia Research Campus, Howard Hughes Medical Institute, Ashburn, VA 20147, USA; 6 Zuckerman Institute, Columbia University, New York, United States; 7 Department of Neurobiology, Harvard Medical School, Boston, MA 02115, USA; 8 Brain Mind Institute & Institute of Bioengineering, EPFL, 1015 Lausanne, Switzerland; 9 Department of Physiology and Biophysics, University of Washington, Seattle, WA, USA; 10 Princeton Neuroscience Institute, Princeton University, Princeton, USA; 11 Computer Science Department, Princeton University, USA; 12 Centre for Neural Circuits and Behaviour, The University of Oxford, Tinsley Building, Mansfield Road, Oxford OX1 3SR, UK; 13 FM Kirby Neurobiology Center, Boston Children’s Hospital, Boston, MA, USA

## Abstract

In most complex nervous systems there is a clear anatomical separation between the nerve cord, which contains most of the final motor outputs necessary for behaviour, and the brain. In insects, the neck connective is both a physical and information bottleneck connecting the brain and the ventral nerve cord (VNC, spinal cord analogue) and comprises diverse populations of descending (DN), ascending (AN) and sensory ascending neurons, which are crucial for sensorimotor signalling and control.

Integrating three separate EM datasets, we now provide a complete connectomic description of the ascending and descending neurons of the female nervous system of *Drosophila* and compare them with neurons of the male nerve cord. Proofread neuronal reconstructions have been matched across hemispheres, datasets and sexes. Crucially, we have also matched 51% of DN cell types to light level data defining specific driver lines as well as classifying all ascending populations.

We use these results to reveal the general architecture, tracts, neuropil innervation and connectivity of neck connective neurons. We observe connected chains of descending and ascending neurons spanning the neck, which may subserve motor sequences. We provide a complete description of sexually dimorphic DN and AN populations, with detailed analysis of circuits implicated in sex-related behaviours, including female ovipositor extrusion (DNp13), male courtship (DNa12/aSP22) and song production (AN hemilineage 08B). Our work represents the first EM-level circuit analyses spanning the entire central nervous system of an adult animal.

## Introduction

For the body to respond to the higher processing commands of the brain, motor and sensory information must be transferred between the brain and VNC. There are 4 principal classes of neurons that traverse the neck. The three most numerous are: ascending neurons (ANs), which have their soma and dendrites in the VNC, feedback information to the central brain; descending neurons (DNs), with the soma and dendrites in the brain, send commands via axons to the ventral nerve cord (VNC); and sensory ascending neurons (SAs) which have their soma outside of the VNC and send some sensory information directly from the periphery to the brain. Finally 18 motor neurons (MNs) exit the neck connective before reaching the nerve cord and target neck muscles in the periphery.

Previous light microscopy (LM) and genetic studies in *Drosophila* have demonstrated that specific behaviours can be mapped to individual neurons and circuits. For the DN population, a range of behaviours are linked to individual or small groups of DNs: aDN, DNg11 and DNg12 for anterior grooming sequences (Guo et al., 2022; Hampel et al., 2015), DNa02 for turning (Rayshubskiy et al., 2020), DNp50/MDN for backwards walking (Bidaye et al., 2014), DNp01/GF for escape (Lima & Miesenböck, 2005), DNp07 and DNp10 for landing (Ache et al., 2019), DNp15/DNHS1, DNp20/DNOVS1, DNp22/DNOVS2 for flight and neck control (Suver et al., 2016) and others. However, our understanding remains incomplete – only a few studies have examined larger groups of DNs by morphology (Namiki et al., 2018) or behaviour (Aymanns et al., 2022; Cande et al., 2018), and even less is known about ANs (Chen et al., 2023).

Male and female *Drosophila* exhibit sexual dimorphism in their behaviour, controlled through dimorphic neuronal circuits in both the brain and the VNC. Differences exist both in the connections made by neurons that are shared between the sexes and in the presence of neurons that appear to be specific to one sex. The sex of each *Drosophila* neuron is determined genetically, primarily through the expression of the transcription factor genes double sex (*dsx*) and fruitless (*fru*). Studies on *fru* and *dsx* expressing neurons and dimorphic behaviours have revealed several sexually dimorphic neurons and small circuits in the brain and VNC. Females, for example, require oviDNs for egg laying (Ache et al., 2019; F. Wang et al., 2020a) and vpoDN to open their vaginal plate when accepting a male (K. Wang et al., 2021). In contrast male-specific P1 central brain neurons control both intermale aggression and courtship steps such as wing extension (Hoopfer et al., 2015; von Philipsborn et al., 2011) while a set of DNs (pIP10 and pMP2) and VNC neurons (TN1a, dMS2, vPR9, dPR1, dMS9, and vMS12) to coordinate time and shape of sine and/or pulse song (Lillvis et al., 2024; Shiozaki et al., 2023; von Philipsborn et al., 2011). However, there has not been any systematic EM level comparison of dimorphic neurons between males and females.

Connectomic datasets are still scarce and although neuronal stereotypy is often cited as an experimental advantage of *Drosophila*, there is little quantitative data on the expected biological variation in connectivity. The first whole brain comparative connectomics work in *Drosophila* (Schlegel et al., 2023) used two female datasets, FAFB-Flywire and the truncated hemibrain dataset, to obtain initial insights into which connections between neurons are conserved across datasets and specimens, estimating the extent of biological variation in connectomes. Until the male adult nerve cord (MANC) dataset, we could only estimate how many neurons connect the brain and the VNC by the available LM lines. 150 years after the first Golgi stainings the neurons of the neck connective as a complete population were described and typed for the first time, revealing 1328 DNs, 1865 ANs and 535 SAs in *Drosophila* (H. S. J. *. Cheong et al., 2023; Marin et al., 2023; Takemura et al., 2023). Comparing EM datasets across individuals, sexes and developmental stages, as done previously in *Caenorhabditis elegans* (Cook et al., 2019; Witvliet et al., 2021), will reveal the degree of variation in *Drosophila* neuronal circuits.

We now describe all the neck connective neurons of the adult female fly brain (FAFB-Flywire) (Chen et al., 2023; Dorkenwald et al., 2023; Schlegel et al., 2023) and the female adult nerve cord (FANC) (Azevedo et al., 2022; Phelps et al., 2021), and compare them to the MANC dataset (H. S. J. *. Cheong et al., 2023; Marin et al., 2023; Takemura et al., 2023). We present the strategies developed to bridge physically disconnected datasets (brain and VNC) and compare datasets of different sexes. Our work represents the first atlas of DNs, ANs and SAs based on EM connectome data. Based on this complete and comprehensively annotated resource, we then illustrate its utility by addressing three scientific questions: First, we investigate the types of sensory information processed by DNs in the brain and the connections between ANs and DNs in the brain and nerve cord. Secondly, we explore stereotypy across the three datasets at the level of morphology and connectivity. Lastly, we examine the implications of unmatched neurons across datasets, particularly in the context of sexual dimorphism.

## Results

### Matching neurons across three datasets

**Fig. 1:**
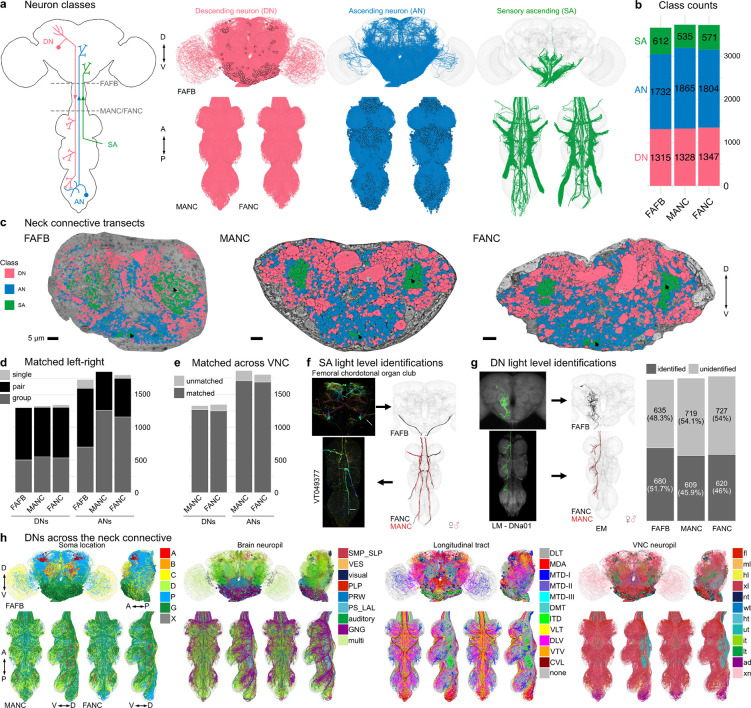
Reconstruction and identification of three neuronal classes across three datasets. **a**, Schematic of the central nervous system with the three neuronal classes that pass through the neck connective: descending neurons (DNs), ascending neurons (ANs) and sensory ascending neurons (SAs). FANC neurons are shown in MANC space here and in all following figures. **b**, Number of neurons in each class and dataset. **c**, Transects through the neck of the three datasets: Female Adult Fly Brain (FAFB), Male Adult Nerve Cord (MANC) and Female Adult Nerve Cord (FANC). These neck connective transects were used as seedplanes to find and reconstruct the three classes of neurons shown in different colours. **d**, Number of DNs and ANs that have been left-right matched into pairs or groups in the three datasets. **e**, Number of DNs and ANs that have a match across the two VNC datasets. **f**, SAs were assigned modalities by matching to light microscopy (LM) images. Left, an example of a LM image of Femoral chordotonal organ club. Right, the EM reconstructions that were matched to the image. **g**, DNs were identified in all three EM datasets by matching the EM reconstructions to LM level descriptions (mainly (Namiki et al. 2018), see supplemental file 2 - DN_identification). Left, an example of a LM image of DNa01 in the brain and VNC and next to it the FAFB, FANC and MANC EM reconstructions that were matched to those images. Right, the quantification of DNs identified in all three datasets. **h**, Identified DNs that can be matched across all three datasets coloured by soma tract, brain neuropil, longitudinal tract and VNC neuropil.

We reconstructed all of neurons that traverse the neck connective in the female adult fly brain (FAFB-Flywire) and the female adult nerve cord (FANC) datasets (see Methods – Neuroglancer Link for details, supplemental file 1 for FAFB and FANC seed planes); we then compared them to the previously published male adult nerve cord (MANC) dataset (H. S. J. *. Cheong et al., 2023; Marin et al., 2023; Takemura et al., 2023) (Fig. 1a). We find that there are between 1315 and 1347 DNs that transmit motor commands and other information from the brain to the VNC; between 1733 and 1865 ANs that report processed sensory and motor state information from the VNC back to the brain; and between 535 and 611 SAs that convey sensory information directly from the periphery to the brain (Fig. 1b). The position of these neurons in the neck connective is morphologically stereotyped, with DNs more dorsal, ANs more ventral, and the SAs localised in two main and two smaller bundles on each side (Fig. 1c, see black arrows and Fig.1 - figure supplemental 1). DNs and ANs were matched across the two sides into pairs or groups in all datasets, and matched between the male and female VNC by their morphology and connectivity (Fig. 1d,e).

We have contributed our proofreading and annotation of these neck connective neurons to the separate online platforms hosting each of these EM datasets. However, we have found that comparisons across datasets are more powerful when each dataset can be visualised simultaneously in the same physical space with a common interface for querying and viewing annotations. We have therefore provided access to co-registered and uniformly annotated neck connective neurons using the Neuroglancer viewer (Maitin-Shepard et al., 2021) (see Methods – Neuroglancer Link). This combined 3D web atlas can be viewed by following https://tinyurl.com/NeckConnective.

Currently available EM datasets comprise either the brain or the VNC and are therefore truncated at the neck during specimen preparation. This creates a considerable challenge for identifying the three primary neuron classes sending projections through the neck. Matching prior light-level descriptions of these neurons to their EM-reconstructed counterparts is necessary for identifying these neurons across EM datasets, bridging brain and VNC, as well as linking the morphology to behavioural data. The ANs and SAs have recently been typed in the male VNC (Marin et al., 2023), but published LM information for these neurons is currently limited, however, by comparing with the available LM images, we were able to assign gross sensory modalities across the neck connective into the brain (Fig. 1f, Fig.1 - figure supplemental 2, Supplementary file 2 - FAFB_SA_identification).

The entire DN population has only been described once in the male VNC EM connectome (H. S. J. *. Cheong et al., 2023). However, there is a significant amount of LM data for DNs (in contrast to ANs or SAs). By overlaying EM morphologies on these LM images, primarily sourced from Namiki et al. (2018) and Namiki et al. (2024, manuscript in preparation), we were able to identify 51.7% of FAFB, 45.9% of MANC and 46% of FANC DNs (supplemental file 2 - DN_identification). By separately matching the brain and VNC portions of a given DN to the same driver line, we were able to bridge connectome datasets. We could therefore analyse input and output, as well as compare between the male and female VNC (Fig. 1g). DNs have previously been grouped by different characteristics and we demonstrate how soma location, longitudinal tract and neuropil innervation compare across the three datasets (Fig. 1h, Fig.1 - figure supplemental 3–6) (H. S. J. *. Cheong et al., 2023). As previously reported (H. S. J. *. Cheong et al., 2023; Namiki et al., 2018), soma location correlates strongly with other features such as axon tract and VNC neuropil innervation; we do note that neurons of the DNa and DNb soma groups innervate a combination of leg or upper tectulum neuropils (Fig.1 - figure supplemental 3a,b). Examining brain and VNC neuropil innervation patterns together highlights some interesting correlations. For example, DNs that innervate the SMP and SLP regions in the brain (higher order processing centres for olfactory stimuli) mainly target the abdominal ganglion of the VNC where they are likely to regulate reproductive or digestive functions (Fig.1 - figure supplemental 4a, 5i). DN axon tracts also highlight interesting groups: e.g. MTD-II tract DNs target the upper tectulum in the VNC (associated with wing pre-motor circuits) and receive input primarily from brain neuropils associated with multimodal integration and steering, the posterior slope (PS) and lateral accessory lobe (LAL). Of course neuropil innervation and tract assignment are still quite coarse organisational features and are only a guide to the function or sensory input for any given DN.

### Sensory input onto descending neurons

**Fig. 2:**
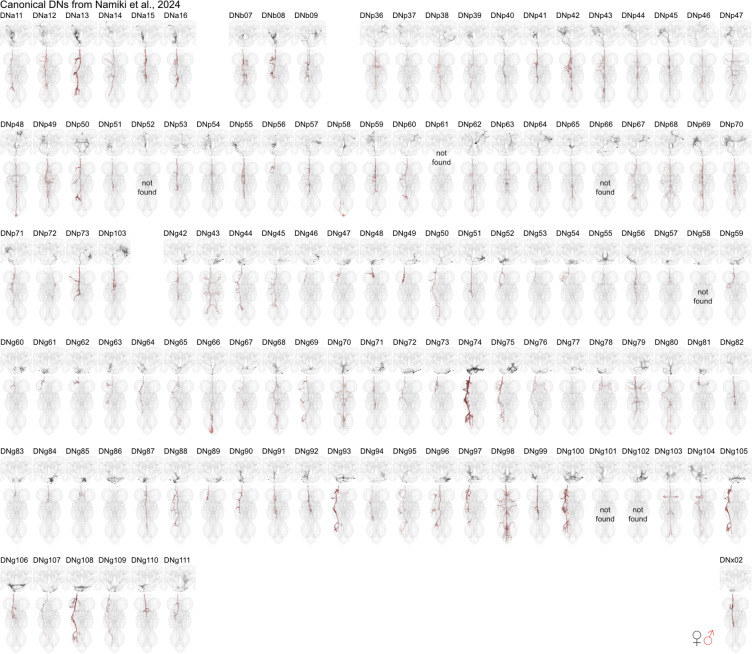
DN matching to Namiki et al. 2024 in prep. Morphology of identified DNs across all three datasets with nomenclature as described in Namiki et al. 2024 in prep. One DN type could not be found as it was duplicated (DNp61) and five could only be found in the brain (DNp52, DNp66, DNg58, DNg101, DNg102). See Fig.2 - figure supplemental 1 for matching to DNs previously characterised at light level by Namiki et al. (2018). See supplementary file 2 - DN_identification for details. In black the morphology of DNs in female datasets (FAFB, FANC) in red from the male dataset (MANC).

By matching our EM reconstructions to LM data and linking them to previously published genetic or electrophysiological studies, we can make new predictions about DN functions and their circuits. DNs have diverse morphologies in the brain and VNC that can be uniquely identified in different EM datasets and LM lines (Fig. 2). Of the 223 DN types identified by LM data, just 2 could not be found in any of our EM datasets while a third turned out to be a duplicate; for 6 LM types we could identify a matching type in the brain but were unsure in the VNC (Fig. 2, Fig.2 - figure supplemental 1, supplementary file 2 - DN_identification).

**Fig. 3:**
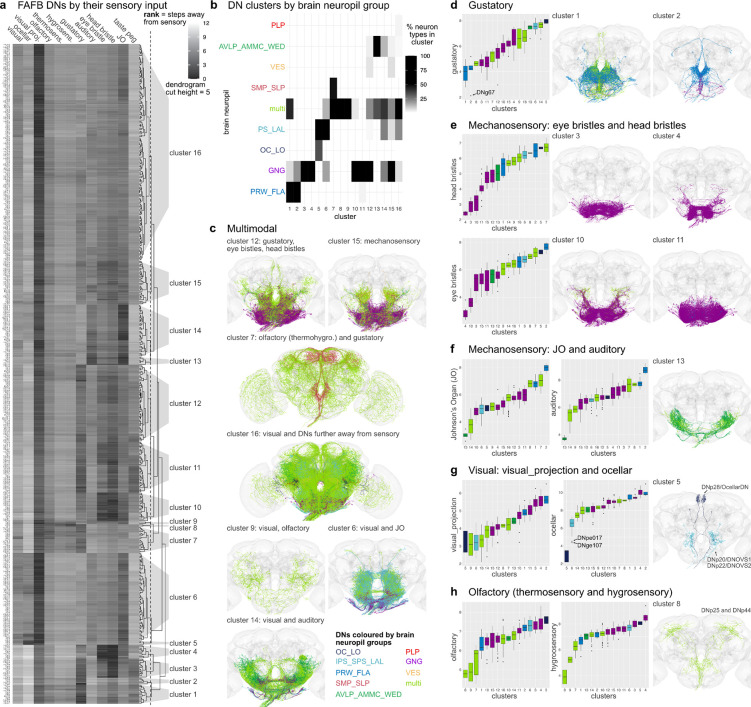
Sensory ranking of descending neurons. Clustering of FAFB DNs by their sensory input rank. **a**, All FAFB DN types, apart from sensory DNs (DNx01, DNx02, LN-DNs), were clustered by their rank to different sensory modalities. The ranks, ranging from 1 to 12, taken from (Dorkenwald et al. 2023) are defined as the traversal distances from a given sensory modality to each DN and then averaged by type. Low rank indicates a more direct connection from sensory modality to DN type. A cut height of 5 (dotted line in dendrogram in a) produces 16 clusters. **b**, Clusters shown in **a** by the brain neuropil assigned to DN types in that cluster in % of all types in that cluster. **c**, DN morphologies of clusters that are close in rank to several sensory modalities in the brain. **d-h**, DN morphologies of clusters that are close in rank to one particular sensory modality. Plots on the left show the average rank of the clusters defined in a for the different sensory modalities. Arrows point to specific DN types that stand out. DN morphologies are plotted in by their brain neuropil colours.

Many DNs identified at light level have been associated with specific behaviours, specific sensory stimuli and evoked motor programs (Simpson, 2024). We have previously described the wide range of motor circuits targeted by the DNs population in the male VNC (H. S. J. *. Cheong et al., 2023). By bridging the neck connective, we can now analyse at scale the sensory information received by DNs in the brain and how that relates to the circuits targeted by their axons in the VNC. Sensory neurons in the FAFB dataset have been annotated extensively and grouped into distinct sensory modalities: visual (photoreceptors and ocellar), olfactory, thermosensory, hygrosensory, auditory, mechanosensory eye bristles, mechanosensory head bristles, mechanosensory Johnson’s Organ (JO), mechanosensory taste peg neurons (Schlegel et al., 2023). We looked at how far DNs are from these sensory modalities in the brain by using the information flow ranking established in Dorkenwald et al. (2023). This analysis excluded 4 DN types that are themselves sensory neurons (DNx01, DNx02, LN-DN1, LN-DN2). DNs were assigned to 16 clusters by their similarity in sensory input (Fig. 3a). DNs in each cluster typically have dendrites in the same brain neuropils (Fig. 3b, Fig.3 - figure supplemental 1 for FAFB neuropil assignments, supplemental file 2 - FAFB_DNs). For example, the two first clusters mostly innervate the prow and flange (Ito et al., 2014), brain regions that receive taste information from the proboscis; these DNs are the closest (lowest average rank) to gustatory sensory neurons based on their modality (Fig. 3a, b, d). Seven of these DN clusters receive a combination of sensory modalities (Fig. 3c) and nine smaller clusters are specific to gustatory, bristle, auditory, ocellar or olfactory sensory information (Fig. 3d–h).

By looking at the DNs previously linked to specific behaviours and from what we know about different neuropils in the *Drosophila* brain, we can assess if these clusters and the sensory modalities assigned to them are reasonable. DNs in cluster 7, for example, integrate olfactory and gustatory information (Fig. 3c); this cluster includes the oviposition-promoting oviDN neurons, which would require exactly this sensory information to select an appropriate nutrient-rich food source (F. Wang et al., 2020a). Interestingly most DNs in cluster 6, receiving a combination of visual and auditory information, innervate the posterior slope (IPS,SPS) and lateral accessory lobes (LAL), brain regions that are known in the insect literature for higher order sensory processing and steering (Currier & Nagel, 2020; Namiki & Kanzaki, 2016; Steinbeck et al., 2020). These two sensory cues would be essential for a navigation behaviour linked to DNs in this cluster, such as DNb06, allowing the fly to turn away from a sound or a visual stimulus, (Yang et al., 2023).

DNs close to mechanosensory inputs fall into three main groups. Four clusters (3,4,10,11) are close to eye and head bristles (Fig. 3e), two (13 and 14) are close to a combination of JO and auditory sensory neurons (Fig. 3f) and cluster 15, receives input from a combination of mechanosensory modalities. This also aligned with the neuropil innervation that is more GNG based for the DNs close to bristle sensory inputs and more WED, AMMC and AVLP for the DNs linked to auditory and JO information. We assume that this large group of DNs would be responsible for the highly targeted grooming of the corresponding bristle locations (Eichler et al., 2024). Most DNs are close to visual stimuli, but only one small cluster (cluster 5, Fig. 3g), containing 3 DN types (DNp28/OcellarDN, DNp20/DNOVS1 and DNp22/DNOVS2), is specific for visual sensory information from the Ocellar. *Drosophila* has three Ocelli, that sense light in addition to the compound eye. Both DNOVS1 and DNOVS2 additionally receive input from optic lobe output neurons that encode pitch-associated or roll-associated optic flow and are involved in fast flight and neck motor control (Suver et al., 2016). Olfactory, thermosensory and hygrosensory information converge onto the same clusters, especially onto cluster 8 (Fig. 3h). There are only 2 DN types in this cluster, DNp25 and DNp44, both previously suggested to be close to olfactory sensory inputs in the hemibrain dataset (Schlegel et al., 2021). The lack of big DN clusters associated specifically with vision or olfaction suggests that this sensory information is more likely to be integrated with other sensory modalities, i.e. olfactory with gustatory, visual with auditory, or preprocessed in higher brain regions further away from DNs, cluster 16. Thus, we are able to separate DN groups by their most likely sensory modalities, pull groups of DNs by their brain innervation, neuropil and sensory integration, and, by matching them to light level lines, follow them into the VNC. Offering insights into the kind of sensory information conveyed to the VNC and its potential functions.

### DN and AN interactions

**Fig. 4:**
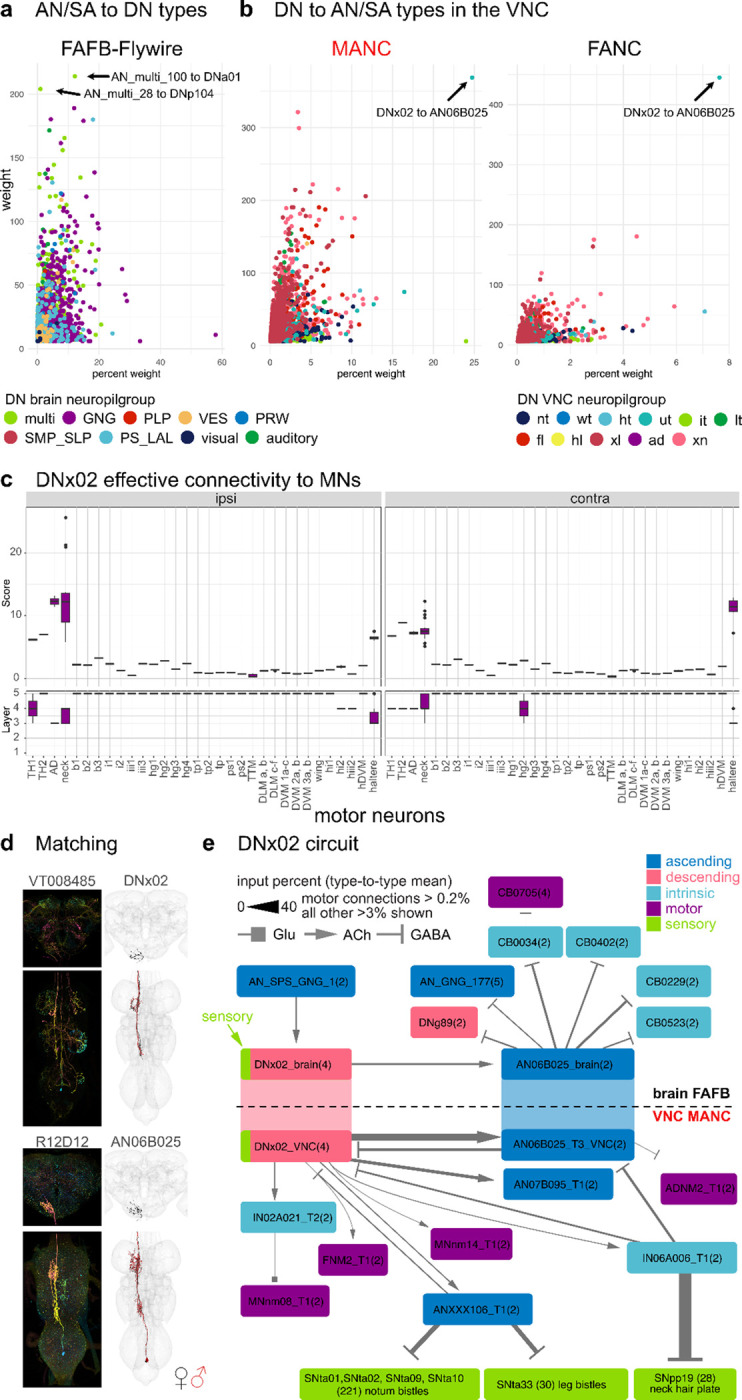
Direct descending and ascending neuron connections. Connectivity of AN/SAs to DNs and vice versa. **a**, Direct connectivity of AN/SAs onto DNs in the brain. DN and AN/SAs connections are averaged by type and plotted by mean weight in percent to mean weight. Arrows point to the two strongest connections in weight from AN/SAs onto DNs. **b**, Direct connectivity of DNs in the VNC onto ANs and SAs. Connections are averaged by type, like in **a**. Arrows point to the one connection that stands out in both MANC and FANC. **c**, The effective connectivity to motor neuron targets ipsilateral and contralateral to the root side of DNx02. **d**, Morphology of DNx02 and AN06B025 in the brain and VNC. In black the EM morphology from female datasets (FAFB, FANC) in red from the male dataset (MANC). **e**, DNx02 circuit in the brain (FAFB-Flywire) and in the VNC (MANC). Connections in both datasets are averaged by type and shown in the percent input to the receiving neuron.

To guide sequences of behaviour to given stimuli, we expect there to be feedback from the VNC ANs back onto DN circuits in the brain. Strong direct connections between DNs and ANs are uncommon in the VNC and the brain (connections above 200 in weight: 2 in FAFB, 9 in MANC, 1 in FANC) (Fig. 4a,c). However, there is one exception that stands out in the VNC, DNx02 output onto AN06B025, which is strong in the number of synapses as well as percent of the total synaptic output (Fig. 4b,c). DNx02 are sensory descending neurons, two on each side, that enter the brain via the occipital nerve, a nerve that was only recently identified (Eichler et al., 2024). Analogously to DNx01, that responds to mechanosensory stimuli on the antenna and is serial to the bilateral campaniform sensillum (bCS) neurons, we predict DNx02 would respond to mechanosensory stimuli from the eye potentially with grooming behaviours (Aymanns et al., 2022; Namiki et al., 2018). DNx02 in the brain stays within the GNG, while in the VNC they project into the neck and haltere neuropils (Fig. 4d). The effective connectivity of DNx02 shows the strong connection onto neck and haltere MNs both ipsi- and contralateral, 2–3 layers into the VNC (Fig. 4c). We were able to find a line for AN06B025 allowing us to identify it in the brain (Fig. 4d, supplemental file 2 - AN_identification). Matching to LM lines for DNx02 and AN06B025 allow us to study this circuit across the neck connective (Fig. 4e).

This revealed that the top target of DNx02 is AN06B025 not only in the VNC but also in the brain and that the GABAergic AN06B025 in turn shuts off DNx02 (Fig. 4e). Additionally, DNx02 targets neck MNs directly and quite strongly via several hops. Two of the neck MNs, FNM2 and ADNM2, have been previously matched to data available from the blowfly, Calliphora erythrocephala (H. S. J. *. Cheong et al., 2023; Strausfeld et al., 1987). The FNM2 in the blowfly is connected to the adductor muscle that moves the head both upwards and inwards while ADNM2 together with the cervical nerve motor neuron (CB0705) innervates the TH2 that control yaw-movement of the head (Kauer et al., 2015; Strausfeld et al., 1987). We propose that DNx02 moves the head upwards and inwards in response to sensory stimuli. It is then inhibited by AN06B025 which inhibits the ADNM2 and disinhibits CB0705 that both project to the TH2 muscle, potentially preparing for an additional sideways deflection of the head. Both movements that could be part of a head grooming sequence. In the brain DNx02 has only one strong upstream partner, which also collects neck information from the VNC (AN_SPS_GNG_1/AN06B057, see supplemental file 2 - AN identification). Through one hop in the VNC DNx02 inhibits sensory neurons coming from bristles (leg and notum) and neck hair plate neurons via an ascending and intrinsic neuron, potentially dampening sensory information from these regions until the grooming movement is complete (Fig. 4e).

This is just one example of the kind of sensorimotor analysis made possible by our matching of brain and VNC neurons through the neck, across the entire central nervous system of *Drosophila*. Future EM datasets that contain a brain with attached VNC will make it possible to look at larger scale feedback loops and sequences of DN and AN activations required for serial behaviours such as grooming (Seeds et al., 2014).

### Stereotypy in the VNC

**Fig. 5:**
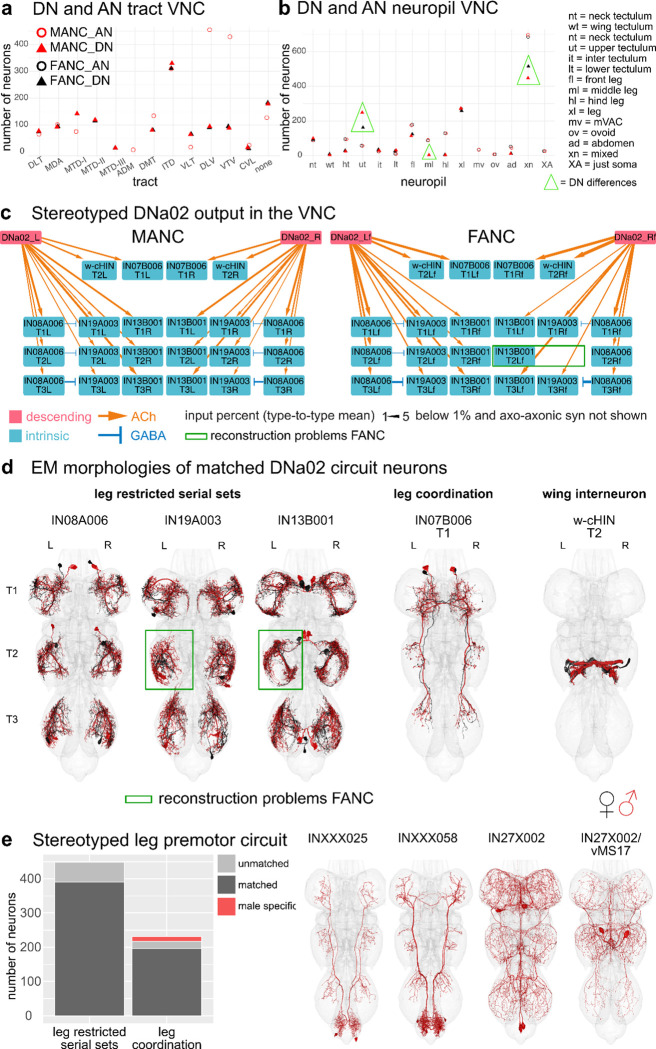
Across VNC dataset comparisons. **a**, Number of DNs/ANs assigned to a tract in MANC and FANC. **b**, Number of DNs/ANs assigned a VNC output/input neuropil in MANC and FANC. **c**, Example of a stereotypes circuit in the VNC. DNa02 in MANC and FANC in percent connect onto 3 sets of serial leg restricted neurons (IN08A006, IN19A003, IN13B001), the w-cHIN and a bilaterally projecting neurons (IN07B006). The types were matched across the two datasets and given the MANC type names accordingly. Downstream targets were selected by receiving more than 2% of DNa02 output. Arrow thickness corresponds to the percent input to the receiving neuron and only values above 1% are shown. **d**, EM morphologies of the neurons shown in the connectivity graphs in **c**. In black reconstructions from FANC in red from MANC. **e**, MANC leg premotor circuit neurons published in (Cheong et al. 2023) matched to FANC. All leg restricted serial sets were found, although some are missing one or the other side in FANC. All apart from 4 types of leg coordination neurons were matched to FANC. EM morphologies of those 4 unmatched types shown on the right as potentially male specific neurons.

One important step for comparing between EM datasets is to assess how stereotyped the morphology and connectivity of neuron types are across the two sides of an animal as well as between animals (Schlegel et al., 2023). From the 223 LM described DN types available to us we matched all but 3 types in the brain and all but 6 types in both VNC datasets, supporting the statement that *Drosophila* neurons are highly stereotyped across individual animals (Fig. 2, supplemental file 2 - DN_indentification). Matching across sides in the 3 datasets and across the two VNC datasets for DNs and ANs (Fig. 1d,e), even when not able to match to LM data, suggest a high degree of stereotypy in these two neuronal classes (matching in supplemental file 2). In addition, we have quantified their consistency in tract and VNC neuropil innervation (Fig. 5a,b, Fig.5 - figure supplemental 1,2). There are a few cases in which the neuropil assignment does not agree across the two VNC datasets. We assume this is a combination of biological variation and the differences in the created neuropil meshes for the two different VNC datasets. For example, the DN type targeting specifically the middle leg neuropil in MANC (DNml) has a considerable amount (>5%) of its synapses in the front leg neuropil in FANC, and is therefore in the neuropil category xl (for multiple leg neuropil innervating) (Fig. 5b, highlighted green triangle). An example of variability that we believe are due to differences in neuropil meshes between the datasets include some of the upper tectulum (ut) DNs in FANC that fall below our 80% synaptic output threshold and thus get assigned to multiple neuropil innervating (xn) (Fig. 5b, highlighted green triangle). Their morphology is, however, unique enough to match with high confidence across the two datasets (supplemental file 2 - FANC_DNs, MANC_DNs).

When normalising the number of synaptic connections using the percent input to the receiving neuron, we can recapitulate a previously analysed circuit of DNa02 (H. S. J. *. Cheong et al., 2023) in the FANC dataset (Fig. 5c, supplemental file 2 - other_MANC_FANC_matches), demonstrating that at a threshold of above 1% we identify the same downstream targets. The downstream partners consist of 3 serially repeated local neuron sets, located in the leg neuropils, a bilaterally projecting neuron, and a connection to the w-cHIN neurons, that control wing MNs. The neurons were matched across the two datasets based on their morphology (Fig. 5d). The connection strengths across the two sides of the animal are comparable to the differences seen across the two datasets, with the exception of two neurons in FANC that are not well reconstructed (Fig. 5c,d, green box). Leg restricted serial sets are preferable when looking for stereotypy as we are able to match sets of 6 or 12 neurons per type (1 or 2 per leg neuropil), rather than individual neurons that might not be found because of reconstruction status or borderline significant differences in morphology. We concentrated on the previously published MANC leg premotor circuit (H. S. J. *. Cheong et al., 2023), as it includes 67 leg restricted serial types (448 neurons) and 75 leg interconnecting types (231 neurons) that are strongly connected to the serially repeated leg MNs. We matched these neurons by morphology and connectivity in the FANC dataset (Fig. 5e, supplemental file 2 - other_MANC_FANC_matches), and while there are many cases in which we have not yet found a match on one or the other side of FANC, we identified matches to all 67 serial sets (Fig. 5e). From the set of 75 interconnecting neurons we did not find any FANC neurons of the following 4 types: INXXX025, INXXX058, IN27X002 and IN27X002/vMS17 (Fig. 5e, morphologies on the right), suggesting that they might be male specific or sexually dimorphic in morphology to the extent that we cannot confidently match them. The first two types (INXXX025 = predicted cholinergic, INXXX058 = predicted GABAergic) project from the abdominal ganglion to the leg neuropils and would be good candidates for male specific leg movements in response to for example abdominal curling. The other two types are very similar to one another and have therefore been given the same systematic type (IN27X002 and IN27X002/vMS17). The neuron with the T2 soma has been identified in LM as vMS17 and is reported to be involved in male courtship song (Lillvis et al., n.d.). Thus, we suggest that the other two neurons of this systematic type regulate similar male specific behaviours.

While complete matching of all VNC neurons across the female and male datasets is still in progress, the neurons we have matched so far (including DNs, ANs, SAs, and the leg premotor circuit, supplemental file 2) exhibit highly stereotyped morphology and connectivity across sides, neuromeres, and datasets. Until now these two datasets have only been analysed independently (Azevedo et al., 2022; Lesser et al., 2024) and MANC (H. S. J. *. Cheong et al., 2023)

### Dimorphism

Some neurons could not be matched across male and female VNC datasets (Fig. 1e, MANC DNs = 59, FANC DNs = 97, MANC ANs = 155, FANC ANs = 115). This could be due to differences in connectome reconstruction, inter-individual biological variation or sexual dimorphism. Previous literature has already identified several sexually dimorphic (sex. dimorphic) and female or male specific neurons (sex-specific) (McKellar et al., 2019; F. Wang et al., 2020a, 2020b; K. Wang et al., 2021). In the following section we will present known sex. dimorphic, and sex-specific DNs, such as the oviDNs, DNp13 and DNa12/aSP22. We defined sex-specific DNs and ANs as neurons that are well reconstructed and can be confidently paired across the two sides of the VNC, but cannot be matched across the VNC datasets. We consider neurons potentially sex. dimorphic if they differ in morphology between the two VNC datasets, but ideally have a confident match across both sides of the nervous system. Both will be referred to as sex-specific or sex. dimorphic in figures and text (details on single neurons can be found in supplemental table 2 -dimorphic_DNs, dimorphic_ANs).

**Fig. 6:**
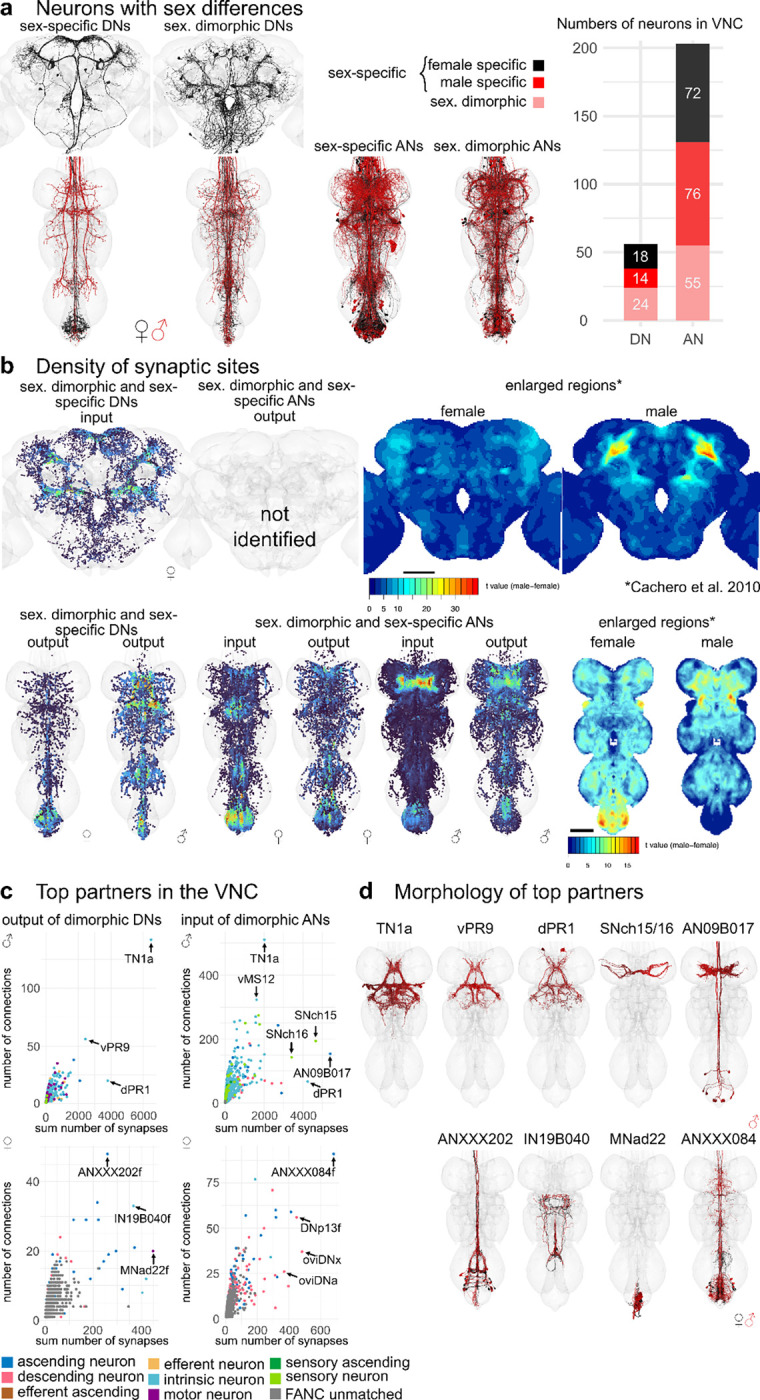
Sex-specific or dimorphic neurons. **a**, Morphology of DNs in three datasets and ANs in the two VNC datasets that are sexually dimorphic (sex. dimorphic) or sexually specific (sex-specific) as described in the literature or predicted by the matching. In black the EM morphology from female datasets (FAFB, FANC) in red from the male dataset (MANC). **b**, Density of pre- or postsynapses of the DNs and ANs shown in a. Compared to previously published images of enlarged regions in female and male central nervous system. **c**, Downstream or upstream partners of sex. dimorphic or sex-specific DNs or ANs respectively in FANC and MANC. Arrows point to the strongest partners by number of synaptic connections and number of neurons connecting onto them. **d**, Reconstructions of neurons pointed out in c in MANC (top row) or in FANC that were matched to MANC neurons of that type.

With the aforementioned definition, we assigned 1% of DNs (18 female/14 male neurons) and 4% of ANs (72 female/76 male neurons) to be sex-specific, as well as 2% of DNs (24 neurons) and 3% of ANs (55 neurons) to be sex. dimorphic in morphology (Fig. 6a). Using this information, we can identify the brain and VNC regions where sex-specific and sex. dimorphic neurons receive and send information (Fig. 6b). In FAFB-Flywire, we observed that input synapses to sex-specific DNs are present, especially in the protocerebral bridge, partially but confidently overlapping with previously published images of enlarged regions in the female fly brain (right panel Fig. 6b) (Cachero et al., 2010). The dimorphic DN output in the VNC also aligns with the enlarged abdominal ganglion in females and the distinct, dimorphic triangular region in males. In females, the input to dimorphic ANs is most pronounced within the abdominal region. Conversely, in males, the input to dimorphic ANs is concentrated in the T1 leg sensory area, as observed in Cachero et al. 2010 (Fig. 6b, right hand side).

Next, we wanted to see if there were many upstream or downstream targets that were shared among neurons that we had defined as dimorphic. For this, we looked at input and output partners to dimorphic DNs and ANs by the sum number of connections to that type, as well as how many times a connection (above a weight of 5) occurred (Fig. 6c,d). The top output partners of dimorphic DNs are all part of the male song circuit: TN1a (silencing decreases sine song), vPR9 (silencing alters the amounts pulse and sine song) and dPR1 (silencing increases sine song) (Lillvis et al., 2024). Top input partners of dimorphic ANs include some of the song circuit neurons already mentioned, as well as two sets of sensory neurons coming from leg taste bristles (SNch15 and SNch16) and AN09B017, which is one of the dimorphic ANs that targets itself and other dimorphic ANs in the same leg sensory area in T1. This type of analysis is difficult without systematically defined cell types that pair neurons across the sides and define functional units. Our preliminary analysis of the FANC dataset found that the top output partners of dimorphic DNs may also be dimorphic. ANXXX202 is dimorphic by its innervation in the abdominal ganglion, IN19B040 has differences in morphology and the abdominal motor neuron MNad22 is dimorphic in connectivity. Input partners to dimorphic ANs are interestingly 3 dimorphic DNs: oviDNa, oviDNx and DNp13, as well as the dimorphic ANXXX084 (see Fig. 6d for morphologies). Our analysis suggests, within the VNC, male dimorphic DNs and ANs predominantly are involved in the song circuit and the response to taste information coming from the front legs while female dimorphic DNs and ANs have important abdominal targets and that the oviDNs require a direct fast feedback via dimorphic ANs.

### Sexually dimorphic DNs involved in courtship and egg laying

**Fig. 7:**
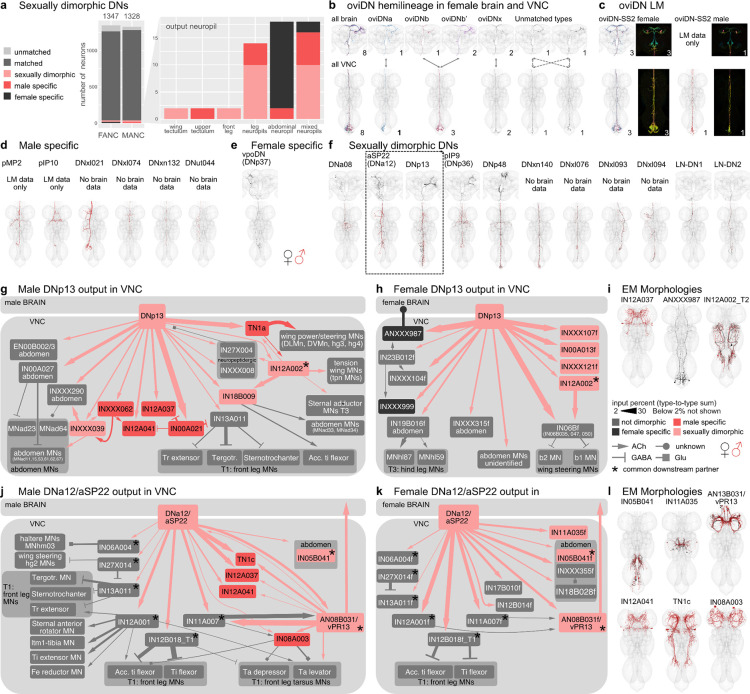
Sexually dimorphic and sex-specific descending neurons. **a**, Proportion of DNs that are sex-specific or sex. dimorphic by dataset and primary input neuropil. **b**, Morphology of DNs in the three datasets belonging to the oviDN hemilineage. **c**, EM morphologies identified within the LM images of female and male of the oviDN-SS2 line. **d**, EM morphologies of previously LM characterised male specific DNs and new potentially male specific DNs. **e**, EM morphology of the female specific DN, vpoDN (DNp37). **f**, EM morphologies of LM characterised sex. dimorphic DNs and new potentially sex. dimorphic DNs. **g**,**h**, Connectivity downstream of the sex. dimorphic DNp13 in MANC (**g**) and FANC (**h**). There is only one downstream partner in common, IN12A002 marked with a *. All other partners do not exist (coloured black or red for sex-specific) or are not downstream of DNp13 in the other dataset (coloured grey). **i**, EM morphology of some of the top VNC targets. **j**,**k**, Connectivity downstream of the sex. dimorphic DNa12/aSP22 in MANC (**j**) and FANC (**k**). There are 8 downstream neurons in common. Only T1 leg motor neurons (MNs) have been systematically identified between the two datasets, thus other FANC Leg MNs are not shown. **l**, EM morphology of some of the top VNC targets. In black the EM morphology from female datasets (FAFB, FANC) in red from the male dataset (MANC). * indicates shared partners downstream of dimorphic DN pairs.

The oviposition-promoting oviDNs are probably the best known female-specific DNs. They have previously been divided into two subtypes based on LM data (F. Wang et al., 2020a). By direct comparison with EM reconstructions, we have defined six female oviDN types and one male type (Fig. 7a,b). Existing LM data were insufficient to match two of the oviDNs across the brain and VNC; EM data clearly distinguish these as two separate types (see Fig. 7a unmatched types). In addition to the oviDNs, one female-specific DN, vpoDN/DNp37 which has previously been described as important in female receptivity by controlling opening of the vaginal plate (K. Wang et al., 2021). We also identified 6 types of male-specific DN. Two of these, pMP2 and pIP10, have previously reported functions in (Kohatsu et al., 2011; Shirangi et al., 2016; von Philipsborn et al., 2011). We also identified 11 DN types which can be found in both male and female datasets but appear sexually dimorphic; for 7 (DNa08, aSP22, DNp13, pIP9, DNp48, LH-DN1 and -DN2) light level matches providing additional evidence for sexual dimorphism (Fig. 7f).

After comprehensive identification of sexually dimorphic DNs, we then carried out analysis of their downstream connectivity. We focused on dimorphic DNs making connections outside of the abdominal ganglion of the VNC due to reconstruction issues in this region of the FANC dataset. We selected DNa12/aSP22 and DNp13 for detailed study; both are fully proofread, present in both sexes, dimorphic in morphology, and have reported roles in both male and female mating behaviours (McKellar et al., 2019; Mezzera et al., 2023; F. Wang et al., 2020b). Robust comparative analysis of the downstream connections from DNs onto VNC neurons requires that these neurons have been both adequately proofread and matched across datasets. Starting from the automated segmentation (Azevedo et al., 2022), at the time of writing the FANC community has proofread just over 5000 neurons (including the 1804 ANs reported in this study) out of the approximately 16,000 intrinsic neurons expected in VNC (Marin et al., 2023). Prior to this work, there has been relatively little matching of precise cell types between the FANC and MANC datasets, with the notable exception of foreleg MNs and wing MNs (Azevedo et al., 2022; H. S. J. *. Cheong et al., 2023). Through our detailed analysis of the MANC and FANC neck connective neurons combined with new computational approaches for intrinsic neurons we have now matched over 4000 neurons across the datasets including top targets of these DNs (supplemental table 2 - other_MANC_FANC_matches, see Methods).

Although identifiable as the same cell type across males and females, DNp13 has highly distinctive morphology and connectivity (Fig. 7f). In both sexes their downstream circuits ultimately target MNs in the wing neuropil and abdominal ganglion. However, these MNs are of distinct types and are targeted via different VNC interneurons in each sex. The top partner of DNp13 in MANC is the male specific double-sex positive neuron TN1a, which is particularly important for sine song (Shirangi et al., 2016), however, we also find that there are several strongly connected wing MNs directly downstream (Fig. 7g). DNp13 in females has been shown to respond to courtship song, and activation of DNp13 leads to ovipositor extrusion (F. Wang et al., 2020b). Unsurprisingly, abdominal MNs are top targets of DNp13 in FANC (Fig. 7h), however, we were surprised to see that the top partners of DNp13 in FANC are three very similar looking IN06B neurons (typeS: IN06B035, IN06B047, IN06B050) that strongly output to b1 and b2 wing MNs (Fig. 7h,i), suggesting that there might be a female wing phenotype that has not yet been described experimentally. This is reminiscent of recent observations that vpoDN may control a wing-spreading behaviour in the evolutionarily related Drosophilid *D. santomea* (Li et al. 2023). The only common downstream target of DNp13 in males and females, with a 2% threshold, is IN12A002 (Fig. 7g,h black star, morphology shown in 7i). IN12A002 has similar morphology in both sexes, but different connectivity between the two circuits suggesting that it is also dimorphic.

DNa12, also known as aSP22, shows greater similarity in connectivity and morphology between the two datasets when compared to DNp13 (Fig. 7j,k). Activation of DNa12 elicits proboscis extension, front leg extension, spontaneous posture adjustments, and abdomen movements in both sexes (McKellar et al., 2019). Interestingly, the type of abdominal movement elicited by DNa12 differs between males (abdominal bending) and females (abdominal extension). In accordance with this work we see that with a threshold of >2% input percent DNa12 neurons share 8/12 MANC and 8/13 FANC downstream partners. The partners that are not shared are also not present when considering all downstream partners with a synapse weight threshold >10. To confirm this, we additionally matched all downstream partners of the MANC DNa12 to those in the FANC dataset. All partners can be matched between the datasets except for the sex-specific TN1c, IN12A037, IN12A041, and IN08A003, which are not targeted by the FANC DNa12.

The connections to the front leg Tibia extensors can be found in both MANC and FANC, in line with the foreleg lifting seen in both sexes (McKellar et al., 2019). DNa12 connects to the sex. dimorphic AN08B031 in both sexes. In turn, AN08B031 has distinct connectivity in males and females. This connection provides an example of a sex. dimorphic AN-DN pair which form sexually diverging circuits while preserving their connection with one another (Fig. 7j,k,l). As this AN cannot be linked to LM, we cannot say if it conveys the proboscis extension that has been described in both sexes in response to DNa12/aSP22 activation. The most surprising connection from the MANC DNa12 is to TN1c, a neuron shown to modulate pulse song (Shirangi et al., 2016) as a phenotype in song has not been reported at time of publication. DNa12 in MANC both directly as well as indirectly connects to TN1c via the sex-specific AN08B043 and AN13B031 (Fig. 7j,k). Two especially interesting neurons that are only downstream of DNa12 in FANC are IN05B041 and INXXX335. They target the abdominal ganglion and we suggest they are responsible for the dimorphic abdomen extension in females (Fig. 7k).

These two examples show that together with the available literature we can now use this LM matching to understand and compare EM circuits across the two VNC datasets, and help us explain the phenotypes observed in behavioural experiments and genetic activation experiments and link them to yet unstudied neurons in the VNC. Moreover, it gives us the chance to make new hypotheses about the function of these neurons, principally suggesting potential roles in song or wing movement that should be looked at experimentally for both DNp13 and DNa12.

### Sex-specific ANs of the 08B hemilineage

While the literature and experimental research have explored some sex. dimorphic and sex-specific DNs, there remains a notable gap in our understanding of ANs in general, particularly with respect to dimorphism. Work on ANs as a population has started to appear, for example ANs encoding behavioural states (Chen et al., 2023; McKellar et al., 2019) but only very few genetically identifiable AN cell types with LM lines are available: LAL-PS-ANs (Fujiwara et al., 2022) and Lco2N1/Les2N1D ANs (Tsubouchi et al., 2017), moonwalker ANs (Bidaye et al., 2014; Tsubouchi et al., 2017) and PERin ANs (Mann et al., 2013). We are not currently aware of any literature reporting sex-specific or sex. dimorphic ANs, therefore all ANs we have annotated are being reported here for the first time (morphologies shown in Fig.8 - figure supplemental 1). We caution that this label is putative: a definitive classification of these ANs as sex. dimorphic or sex-specific will require systematic identification and confirmation with LM data.

**Fig. 8:**
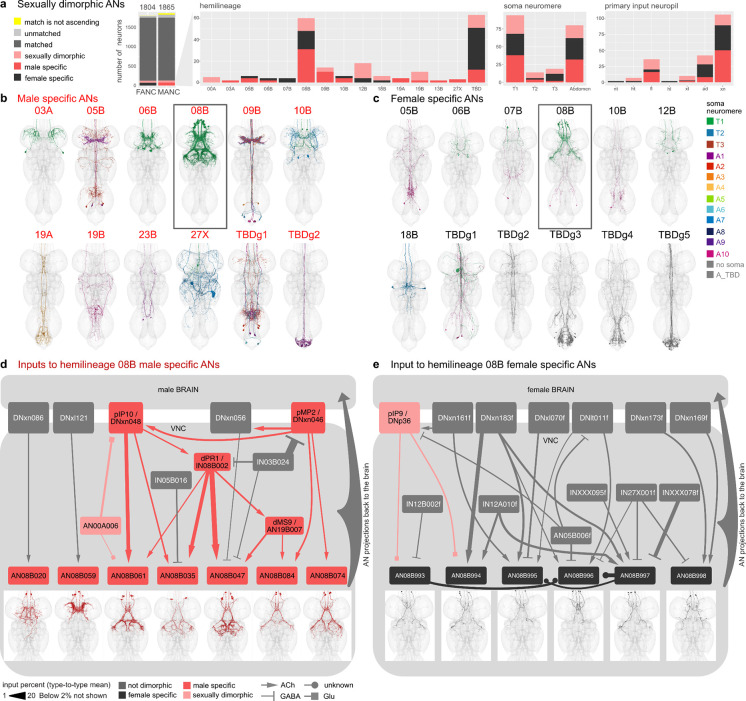
Sexual dimorphism and sex-specific ascending neurons. **a**, Proportion of ANs that are potentially sex-specific or potentially sex. dimorphic by hemilineage, soma neuromere and primary input neuropil. **b**,**c**, Morphology of ANs that are potentially sex-specific in males (**b**) and females (**c**) by hemilineage. FANC neurons were assigned hemilineages and somaneuromere if possible and given new type names. **d**,**e**, Input circuit in the VNC to potentially sex-specific AN types of hemilineage 08B with soma location in T1 (black box in **b** and **c**). Morphology of AN types underneath. All input neurons with more than 2% input onto the receiving AN are shown. FANC neurons in e were matched to MANC neuron types by morphology and connectivity and given the MANC names with an addition of f for female.

We identified the hemilineage and soma neuromere for all dimorphic ANs in FANC when possible, and compared the number of dimorphic ANs across the two datasets (Fig. 8a). We found that the hemilineages with the highest number of dimorphic ANs are the hemilineage 08B and the ANs in the abdominal ganglion, which we were unable to assign a hemilineage to in either MANC or FANC. To avoid reconstruction issues in the abdominal ganglion, we focused our analyses on sex-specific 08B neurons in both datasets. The sex-specific ANs, both in MANC and FANC are the only sex-specific ANs that clearly innervate the mesothoracic triangle associated with dimorphic neurons involved in male song production, which include pMP2 or vPR1 (Yu et al., 2010) (Fig. 8b,c). The male spec. 08B ANs consist of 7 types (Marin et al., 2023); here, we categorised the female spec. 08B ANs into 6 types based on morphology and connectivity (Fig. 8d,e). For the male spec. ANs, we see that 5 of them connect into an interconnected circuit including the dimorphic DNs pMP2 and piP10, IN dPR1 and sex. dimorphic AN19B007/dMS9, thus we conclude that they are all involved in the male song circuit as expected by their innervation pattern. AN08B020 and AN08B059, on the other hand, only have connections from two DNs, neither of which have been associated to song production at this time point, but could be involved in a different male specific behaviour based on their innervation (Fig. 8d). The upstream circuit to female sex-specific 08B ANs does not include any of the neurons upstream of the MANC sex-specific 08B ANs. This supports the idea that these neurons differ both in terms of their morphology and connectivity. Another supporting factor is that the known sexually dimorphic fru+ pIP9/DNp36 (Yu et al., 2010) inputs onto two of the AN types. The other neurons upstream in FANC all exist in MANC but do not connect to any ANs of the 08B hemilineage.

We conclude that 5 out of the 7 newly identified types of male-specific 08B ANs are important for providing feedback during male song production. This may represent another example of ANs acting as a corollary discharge to suppress the auditory response to self-generated song (Poulet and Hedwig 2006; H. S. J. Cheong et al. 2024). Conversely, the female spec. 08B ANs transmit feedback about sexually dimorphic information (such as mating state) back to the brain, a pathway which either takes a different course or does not exist in the male nervous system. Here, we present two strategies of dimorphic circuits in the nervous system: 1) Sex-specific neurons interact with one another to produce a sex-specific behaviour, and 2) circuit elements present in both sexes interact with sex-specific neurons to establish representations which are necessary in one sex but not the other.

## Conclusion

Through detailed reconstruction of DNs, ANs, and SA neurons across three EM connectome datasets, we present, for the first time, a complete set of these neuronal classes spanning the neck connective. We have categorised the neurons by sensory modality (for FAFB DNs, subclassification of SAs), neuropil innervation across the brain and nerve cord, as well as tract and soma location. We have established a platform for systematic neuron typing based on light-level and cross-dataset identification, which we encourage the community to utilise for studying their specific circuits of interest. Pre-publication access to our work has already benefited a number of publications (Braun et al., 2023; H. S. J. Cheong et al., 2024; Dallmann et al., 2024; Lee et al., 2024; Lesser et al., 2024; Sapkal et al., 2023; Yang et al., 2023).

The sensorimotor reconstructions presented enable us to infer the circuit basis of behaviours, including sexually dimorphic patterns, allowing us to formulate new hypotheses regarding numerous circuit components. Future studies, whether at EM, LM or functional genetic level (e.g. calcium imaging) of any part of the CNS can now use this resource to link their work with the connectome. While previous studies have often focused on a single neuropil of the brain, i.e. Antennal Lobe for odour processing or Mushroom Body for learning and memory, this approach overlooks the full complexity of sensory inputs, underlying sensorimotor circuits and behavioural sequences. We anticipate that future research will adopt similar whole CNS approaches and require the integration of several datasets as demonstrated here.

Despite the common challenge of small sample sizes in connectomics, our study stands out for its utilisation of multiple datasets from multiple individuals (also see (Schlegel et al., 2023) for comparative FAFB-hemibrain and (Valdes-Aleman et al., 2021) for an example from the *Drosophila* larva). By systematically comparing DNs, ANs and SAs from both male (MANC) and female (FANC) VNC datasets, we categorised similarities and differences between the two sexes. This represents the first comprehensive comparison of *Drosophila* neuronal morphology and connectivity between sexes at EM resolution. We have identified all previously published dimorphic DNs, and describe the circuits of DNa12 and DNp13 in both datasets. Moreover, we have excluded and annotated all differences that we believe are due to biological variation between individuals (variation in numbers, single, missing or additional neurons of a type) or reconstruction state in one of the two datasets. Our findings suggest potential sex-specific or sex. dimorphic DNs and ANs, with a specific focus on circuits of sex-specific ANs from the 08B hemilineage, associated with male song during courtship, laying the groundwork for understanding the circuitry underlying sex-related behaviours.

## Methods

### Neck connective proofreading and annotation

We defined a perpendicular plane through the neck connective posterior to the cervical nerve for the two VNC datasets (MANC and FANC), and anterior to the cervical nerve for the FAFB dataset. Supplemental file 1 includes two tables, FANC_seed_plane and FAFB_seed_plane, that list all profiles with their xyz coordinates in this plane, ids and the neuronal class. Every neuronal profile passing through these planes in FANC or FAFB was individually reviewed, reconstructed and annotated by manual proofreading of the corresponding automated segmentations. We reviewed 3874 profiles (which received a total of 100,747 edits) in FANC, and 3693 profiles (which received 131,207 edits) in FAFB. Both datasets provide open community-based proofreading platforms (see https://flywire.ai/ and https://github.com/htem/FANC_auto_recon/wiki), and some of these edits were due to general proofreading in each volume, but the majority were from our comprehensive proofreading of neck connective neuron. The first pass review of the MANC neck connective was carried out in mid 2021; for FAFB the initial review periods were late 2020/early 2021 and again in mid 2022. After initial review of ANs and SAs in the VNC datasets, neurons were assigned a putative soma side programmatically, directly or indirectly via a MANC mirroring registration (H. S. J. *. Cheong et al., 2023). Neurons were mirrored based on their soma side or their neck plane side and NBLAST clustered (Costa et al., 2016). This analysis allowed for an initial grouping of left-right homologous sets and to identify neurons with different morphologies on each side of the nervous system, triggering further proofreading (since these differences usually resulted from residual segmentation errors). The combination of comprehensive proofreading of the whole dataset followed by within dataset matching and focussed proofreading was essential to ensure high quality connectome data and annotation. Most DNs and ANs have a unique morphology and were grouped into pairs, otherwise neurons were combined into larger groups containing more than one neuron per side. This was especially the case for SA neurons in FAFB. A similar approach has recently been described for MANC (H. S. J. *. Cheong et al., 2023). Note that proofreading across the FlyWire-FAFB dataset was reported in aggregate in (Dorkenwald et al., 2023) and that a first version of the neck connective annotations was released as part of the brainwide FlyWire annotations paper (Schlegel et al., 2023).

### Light microscopy identification

DNs from LM images were identified by overlaying the EM reconstructed DNs with images of Gal4 lines, mainly from the Namiki collection ((Namiki et al., 2018) and in preparation Namiki et al. 2024), Janelia’s Gal4 and Split-Gal4 collections (Jenett et al., 2012; Meissner et al., 2024; Tirian & Dickson, 2017), or via the neuronbridge tool (Clements et al., 2022; Meissner et al., 2023) for MANC DNs. To compare the reconstructions and LM images in the same space, the latter were segmented and transformed into MANC space as described in (H. S. J. *. Cheong et al., 2023) or into FAFB space. The full list of DN types with the identifier for the LM image (slide_code) and for the type (VFB_ID) can be found in supplemental file 2 - DN_identification. A small list of ANs were also matched to LM in FAFB and MANC as they were of special interest for the circuit described in Fig. 3 (see supplemental file 2 - AN_indentification). We did not match ANs to LM images systematically, due to a lack of a catalogue describing these neurons (as is available for DNs; (Namiki et al., 2018)). SA neurons were divided into subclasses by comparing them to LM images of Janelia’s Gal4 and Split-Gal4 datasets using the neuronbridge tool (Clements et al., 2022; Meissner et al., 2023) for MANC and then manually matching their axonal continuations into the brain to FAFB neuron reconstructions. Fig.1 - figure supplemental 2 shows the LM line that SA neurons were matched to, as well as the assigned long_tract and entry_nerve that were used to give SA neurons a subclass name and aided the identification (see also supplemental file 2 - FAFB_SA_identification).

The process of matching to LM data is not exhaustive (in part because LM data is not yet available for all neurons) and we kindly ask the *Drosophila* community to contact the authors with missing identifications which can be reviewed and integrated in this resource.

### Matching of neurons across VNC datasets

FANC DNs and ANs were transformed into MANC space using the transform_fanc2manc function from the fancr R package (https://github.com/flyconnectome/fancr). This is a one step thin plate spline transform based on 2110 landmark pairs fitted to a complex transformation sequence mapping FANC to the JRCVNC2018F template (Phelps et al., 2021) and JRCVNC2018F to MANC (Takemura et al., 2023). A combination of NBLAST (Costa et al., 2016), and connectivity analysis was used to identify candidate morphological matches. These were assessed manually and assigned MANC names if the match was of high confidence (confidences ranged from 1 to 5, high is >3, Supplemental file 2). Additionally all ANs that were not matched with high confidence were assigned hemilineage and soma location in FANC and were compared by two independent annotators within each hemilineage after thorough review of the non-matching ANs to exclude reconstruction problems as a cause. ANs were first matched between FANC and MANC as individual neurons. We then reviewed these MANC-FANC matches to ensure that they respected the groups of neurons previously defined in MANC, thus providing an additional layer of validation. Cosine similarity as well as the identity of strong upstream and downstream synapses partners was used to help resolve ambiguous cases.

### Tract identification

VNC longitudinal tracts for MANC ANs, MANC SAs and FANC DNs were identified as previously described in (H. S. J. *. Cheong et al., 2023). In brief, neurons were simplified to their longest neurite starting from the VNC entry point at the neck and subsequently NBLAST clustered (Costa et al., 2016). The clusters were manually assigned a tract by overlaying with tract meshes made for MANC (H. S. J. *. Cheong et al., 2023).

Analysis of AN tracts revealed for the first time that one cluster did not match any of the previously published DN tracts. This new tract was given the name **A**N specific **d**orsal **m**edial tract (ADM) in accordance with the tract naming of (Court et al., 2020).

### Neuropil identification

Primary brain neuropils were assigned in the FAFB dataset using the per neuron neuropil counts of presynapses for ANs and the postsynapses for DNs available for the 783 FAFB version (available for download at https://codex.flywire.ai/api/download). A single brain neuropil was assigned if 80% of all synapses were within that neuropil, two neuropils were assigned as a name (primaryneuropil_secondaryneuropil) if combined they reached the 80% threshold and each contained at least 5%. An assignment as *multi* was given to 367 DNs and 282 ANs as they collected input or gave significant output (>20%) to more than two neuropils.

Primary VNC neuropils were assigned to all DNs and ANs in the MANC/FANC datasets as previously performed for MANC DNs (H. S. J. *. Cheong et al., 2023). For MANC AN synapses, we used the neuprint synapse ROI information of the manc:v1.2. For FANC AN and DN synapses we retrieved the synapses allocated to AN and DN IDs from the synapse parquet file, retrievable via FANC CAVE and available from the FANC community upon request (provided by Stephan Gerhard).

A single neuropil abbreviation was given to a DN/AN if they innervated a VNC neuropil with >80% of their pre/post synapses. The two letter abbreviations nt, wt, hl, it, lt, fl, ml, hl, mv, ov, ad correspond to NTct, WTct, HTct, IntTct, LTct, LegNpT1, LegNpT2, LegNpT3, mVAC, Ov and ANm, respectively. Additionally DNs/ANs which innervated a combination of upper tectulum (ut) or leg neuropils (xl) with more than 80% of their pre/post synapses were given those abbreviations accordingly. Any neuron that did not fall into one of those two categories was grouped as xn, standing for multiple neuropils. ANs that only contained a soma and soma tract in the VNC were excluded from this neuropil analysis and referred to as XA as previously described (Marin et al., 2023).

If the neuropil names were inconsistent within a group or pair of neurons, we calculated the mean of the pre- or post synapses to determine the assignment.

### Information flow ranking

The information flow ranking previously reported by Dorkenwald et al. (2023) for FlyWire, was subsetted for descending neurons and averaged by DN type. The information flow analysis is based on an algorithm implemented in Schlegel et al. (2021) (https://github.com/navis-org/navis). A low rank indicates a more direct connection from sensory inputs to that DN type.

### Sexually dimorphic and sex-specific neurons

DNs previously described to be sex-specific such as the female specific oviDNs or male specific pIP1 were matched to the available light level data and referred to as **sex-specific** throughout the paper. Other DNs and ANs that we could not match between the VNC datasets (between female and male), couldn’t be matched to light level data, but were well reconstructed and had a left-right partner were considered to be potentially female/male specific, also referred to in text and figures as **sex-specific**.

DNs such as DNa08 that exist in both sexes but are known to be dimorphic in morphology were matched to light level data and referred to as **sex. dimorphic**. Other DNs and ANs that we could confidently match across the two VNC datasets but that were dimorphic in morphology are also referred to as **sex. dimorphic**. The following neurons were not considered even though they show morphological differences:

- Specifically, neurons presumed to be neuropeptidergic were not included, as big morphological differences in neuronal arbour are common even between left and right of the same animal (Marin et al., 2023).
- The ascending histaminergic neurons (AHNs), which have been shown to have a difference in morphology not related to the sex of the animal (H. S. J. Cheong et al., 2024).
- Those neurons that innervate the abdominal ganglion, where there are problems in the FANC dataset that make it impossible to distinguish between a difference in reconstruction state and potential dimorphism, noted as reconstruction issues in the supplementary tables.
- Differences in number of ANs or DNs of a type were not considered as dimorphism in this paper as they occurred in neurons that we consider populations and whose numbers differed across the two sides of one animal. A difference in number was noted in the supplementary tables as biological variation, a match that is not ascending, or a general matching problem, if one side was not found (Supplemental file 2).

### Synapse density plots

To calculate the synapse density of sexually dimorphic and sex-specific neurons in the VNC we collected all synapses of the identified neurons in each dataset (FAFB: cleft-score > 50 applied). We then tiled the space their synapses occupy into roughly isotropic voxels of 5 μm size and counted synapses in each voxel. Synapses were then colour coded by density and plotted in three-dimensional space.

### FANC neuron types

All FANC neurons that can be matched to MANC neurons are referred to by their MANC name, with an additional “f” denoting female, when presented in comparative graphs or connectivity plots. All FANC neurons identified are listed in the supplementary material (Supplemental file 2 - FANC_DNs, FANC_ANs, other_MANC_FANC_matches).

FANC ANs and DNs that were not previously identified in LM and that could not be matched between datasets were assigned new type names. For ANs and DNs, the type names were given in accordance with the previously established systematic type names (DN-target neuropil abbreviation-number or AN-hemilineage abbreviation-number). To distinguish from the previous type names, the numbering starts at 999 and goes down.

### Connectivity

For connectivity graphs we used a threshold of weight >10 and percent output >0.5% for the initial retrieval of partners of the neurons of interest. In the following step we added all MNs, SNs or SAs that connected to those with a weight >5 to adjust for known reconstruction problems in these neurons and the fact that sensory neurons tend to make fewer synapses with their partners individually and connect as a population of the same sensory origin (reflected by their type). Once all neurons of interest had been defined we took an all-by-all connectivity adjacency matrix, in which all values were converted to input percent to the receiving neuron, averaged by type (unless otherwise indicated). The graphs shown in the figures note the additional percent thresholds that were chosen for the nodes plotted in the graph.

### Neuroglancer Resource

To help compare the neurons described in our work we created a neuroglancer environment (Maitin-Shepard et al., 2021) displaying meshes for all three datasets in a common space. This environment can be opened in any modern web browser (we use Google Chrome) by following the short URL https://tinyurl.com/NeckConnective.

We opted to use the Janelia FlyEM male CNS dataset as a single anatomically consistent target space for display based on resources provided by (Nern et al., 2024). FlyWire neurons were transformed into the space of the male CNS brain using rigid and non rigid consecutive registrations (Bates et al., 2020; Nern et al., 2024). Meshes for MANC neurons are those released by (Takemura et al. 2023); we then use neuroglancer to apply an affine registration “on-the-fly” to place them within the space of the VNC of the male CNS volume. We applied non-rigid transformations (fancr::transform_fanc2manc function described above) to put FANC neurons into MANC space and then used the same MANC to male CNS affine registration within neuroglancer to complete the transformation into male CNS space. Metadata annotations are provided for the three datasets using the format Type_Side_Class format. At present only the optic lobe portion of the male CNS EM volume has been released but having all the data transformed into male CNS space means that this neuroglancer scene can be modified with minimal effort to display the full male dataset when it becomes available.

## Supplementary Material

Supplement 1

Supplement 2

Supplement 3

Supplement 4
