# Supplementary material for: Comparative connectomics of the descending and ascending neurons of the Drosophila nervous system: stereotypy and sexual dimorphism
Source: bioRxiv. 2024 Jun 28:2024.06.04.596633. Originally published 2024 Jun 6. Preprint. [Version 2] doi: 10.1101/2024.06.04.596633 (PMC11185702; doi:10.1101/2024.06.04.596633)
Supplement: Supplement 4 [file media-4.zip › Fig8-dimorphic_ANs_formatted1200.pdf]

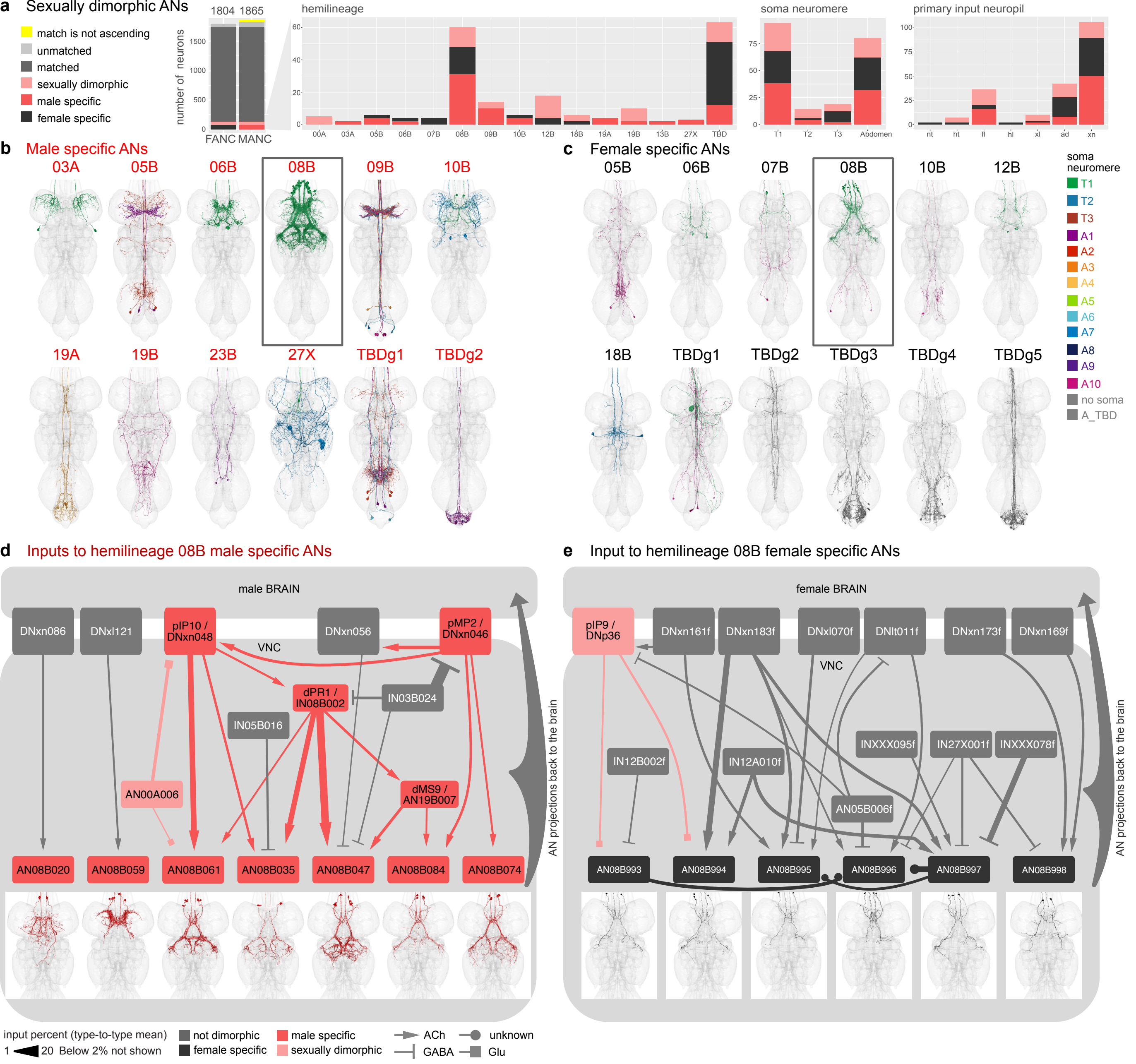

a Sexually dimorphic ANs
match is not ascending
unmatched
matched
sexually dimorphic
male specific
female specific
1804 1865
number of neurons
FANC MANC
hemilineage
soma neuromere
primary input neuropil
00A 03A 05B 06B 07B 08B 09B 10B 12B 18B 19A 19B 13B 27X TBD
T1 T2 T3 Abdomen
nt ht fl hl xl ad xn
b Male specific ANs
03A 05B 06B 08B 09B 10B
19A 19B 23B 27X TBDg1 TBDg2
c Female specific ANs
05B 06B 07B 08B 10B 12B
18B TBDg1 TBDg2 TBDg3 TBDg4 TBDg5
soma neuromere
T1 T2 T3 A1 A2 A3 A4 A5 A6 A7 A8 A9 A10 no soma A_TBD
d Inputs to hemilineage 08B male specific ANs
male BRAIN
DNxn086 DNxl121 pIP10 / DNxn048 DNxn056 pMP2 / DNxn046
VNC
dPR1 / IN08B002 IN03B024 IN05B016 AN00A006 dMS9 / AN19B007
AN08B020 AN08B059 AN08B061 AN08B035 AN08B047 AN08B084 AN08B074
AN projections back to the brain
e Input to hemilineage 08B female specific ANs
female BRAIN
pIP9 / DNp36 DNxn161f DNxn183f DNxl070f DNlt011f DNxn173f DNxn169f
VNC
IN12B002f IN12A010f INXXX095f IN27X001f INXXX078f AN05B006f
AN08B993 AN08B994 AN08B995 AN08B996 AN08B997 AN08B998
AN projections back to the brain
input percent (type-to-type mean)
1 20 Below 2% not shown
not dimorphic
female specific
male specific
sexually dimorphic
ACh
GABA
unknown
Glu
