# Supplementary material for: Comparative connectomics of the descending and ascending neurons of the Drosophila nervous system: stereotypy and sexual dimorphism
Source: bioRxiv. 2024 Jun 28:2024.06.04.596633. Originally published 2024 Jun 6. Preprint. [Version 2] doi: 10.1101/2024.06.04.596633 (PMC11185702; doi:10.1101/2024.06.04.596633)
Supplement: Supplement 4 [file media-4.zip › Extended_Data_Fig2_formatted600.pdf]

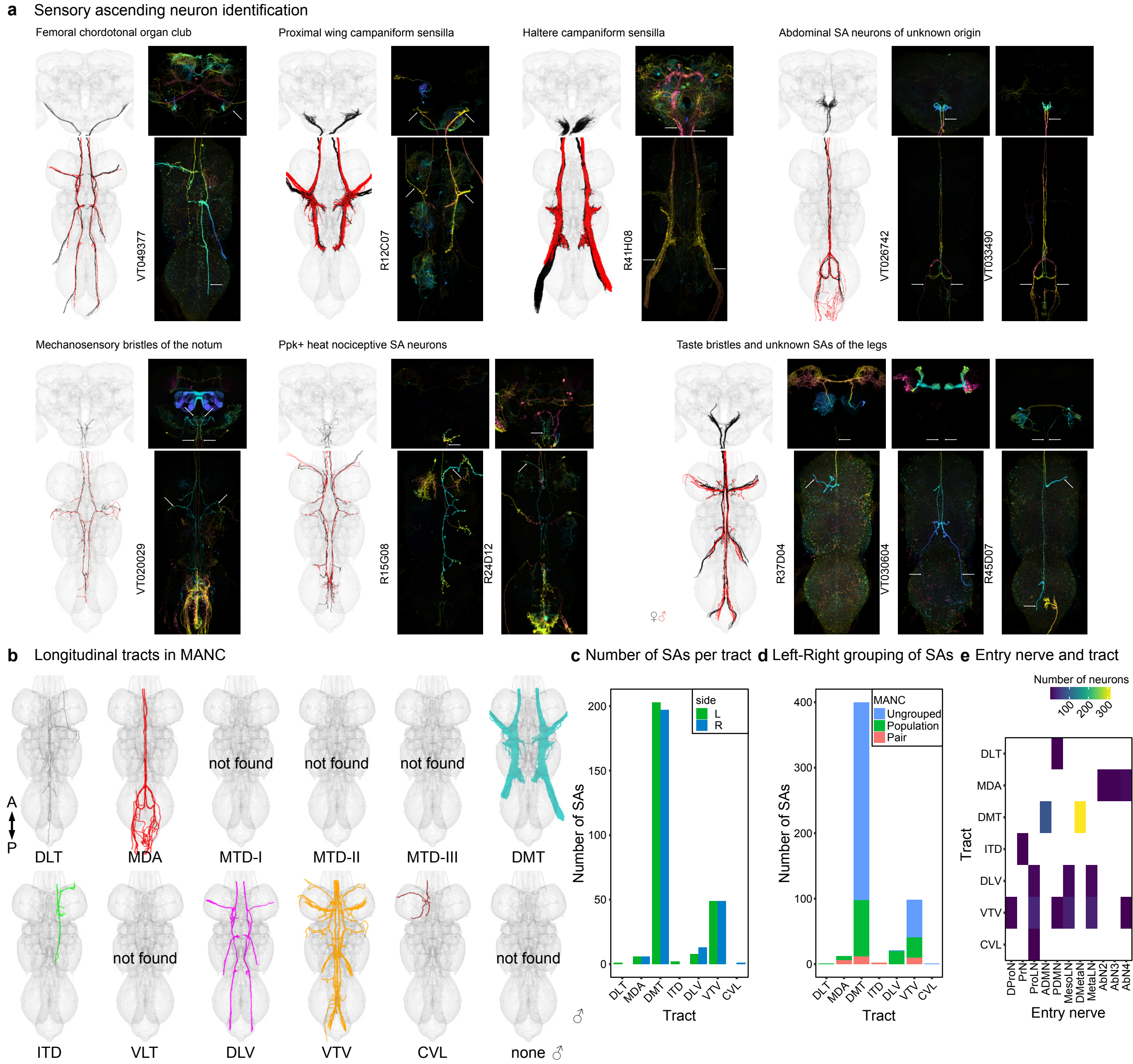
a Sensory ascending neuron identification
Femoral chordotonal organ club
Proximal wing campaniform sensilla
Haltere campaniform sensilla
Abdominal SA neurons of unknown origin
VT049377
R12C07
R41H08
VT026742
VT033490
Mechanosensory bristles of the notum
Ppk+ heat nociceptive SA neurons
Taste bristles and unknown SAs of the legs
VT020029
R15G08
R24D12
R37D04
VT030604
R45D07
♀♂
b Longitudinal tracts in MANC
A
P
DLT
MDA
MTD-I
MTD-II
MTD-III
DMT
not found
ITD
VLT
DLV
VTV
CVL
none ♂
c Number of SAs per tract
side
L
R
Number of SAs
Tract
0
50
100
150
200
DLT
MDA
DMT
ITD
DLV
VTV
CVL
♂
d Left-Right grouping of SAs
MANC
Ungrouped
Population
Pair
Number of SAs
Tract
0
100
200
300
400
e Entry nerve and tract
Number of neurons
100
200
300
Tract
Entry nerve
DProN
PrN
ProLN
ADMN
PDMN
MesoLN
DMetaN
MetaLN
AbN2
AbN3
AbN4
