# Supplementary material for: Comparative connectomics of the descending and ascending neurons of the Drosophila nervous system: stereotypy and sexual dimorphism
Source: bioRxiv. 2024 Jun 28:2024.06.04.596633. Originally published 2024 Jun 6. Preprint. [Version 2] doi: 10.1101/2024.06.04.596633 (PMC11185702; doi:10.1101/2024.06.04.596633)
Supplement: Supplement 4 [file media-4.zip › Extended_Data_Fig4_formatted600.pdf]

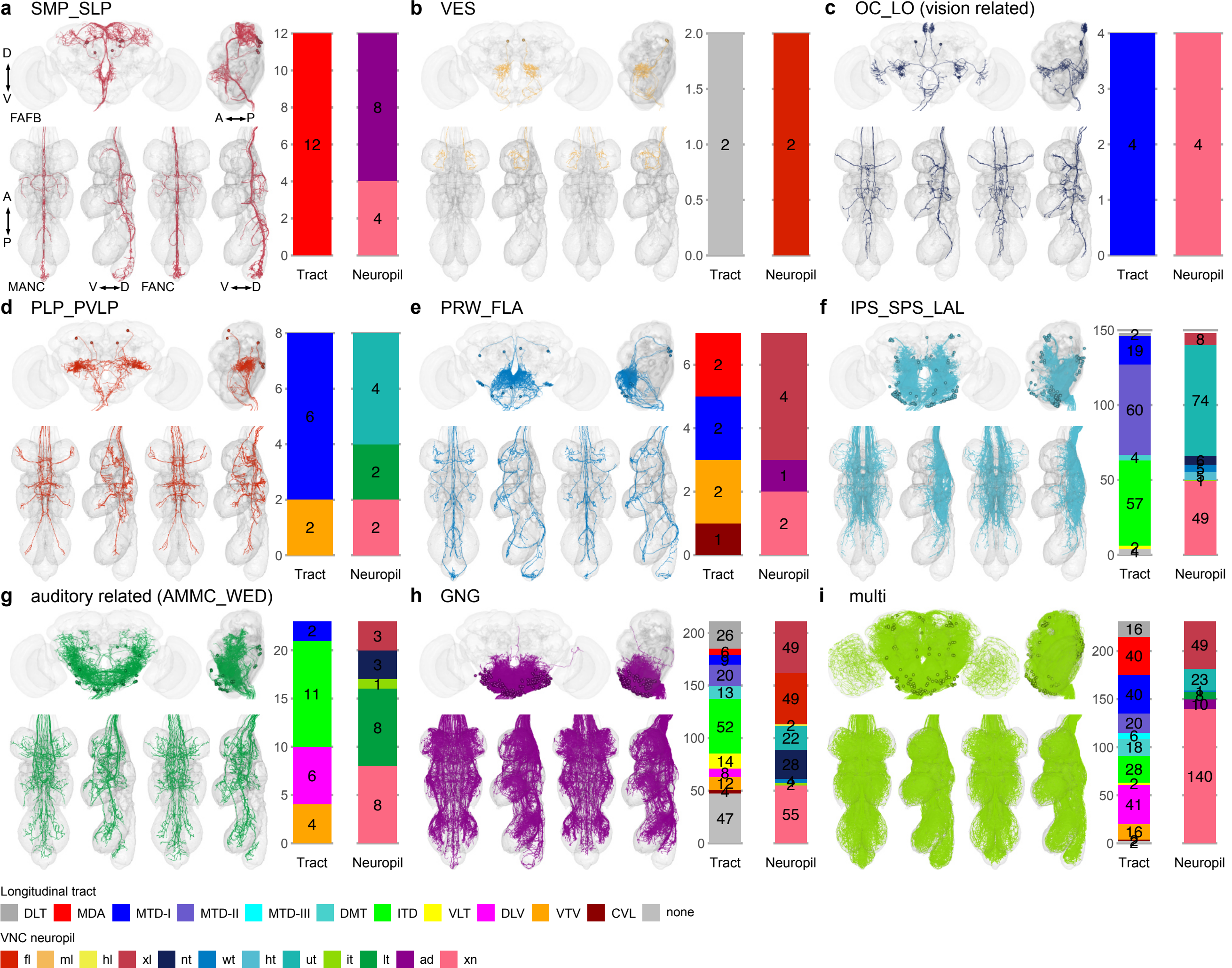
a SMP_SLP
D
V
FAFB
A
P
MANC
V D
FANC
V D
A P
12
8
4
Tract
Neuropil
b VES
2
2
Tract
Neuropil
c OC_LO (vision related)
4
4
Tract
Neuropil
d PLP_PVLP
6
2
4
2
2
Tract
Neuropil
e PRW_FLA
2
2
2
1
4
1
2
Tract
Neuropil
f IPS_SPS_LAL
19
60
4
57
8
74
49
Tract
Neuropil
g auditory related (AMMC_WED)
2
11
6
4
3
3
1
8
8
Tract
Neuropil
h GNG
26
20
13
52
14
8
12
47
49
49
22
28
55
Tract
Neuropil
i multi
16
40
40
20
6
18
28
41
16
49
23
10
140
Tract
Neuropil
Longitudinal tract
DLT
MDA
MTD-I
MTD-II
MTD-III
DMT
ITD
VLT
DLV
VTV
CVL
none
VNC neuropil
fl
ml
hl
xl
nt
wt
ht
ut
it
lt
ad
xn
