# Supplementary material for: Comparative connectomics of the descending and ascending neurons of the Drosophila nervous system: stereotypy and sexual dimorphism
Source: bioRxiv. 2024 Jun 28:2024.06.04.596633. Originally published 2024 Jun 6. Preprint. [Version 2] doi: 10.1101/2024.06.04.596633 (PMC11185702; doi:10.1101/2024.06.04.596633)
Supplement: Supplement 4 [file media-4.zip › Extended_Data_Fig10_formatted600.pdf]

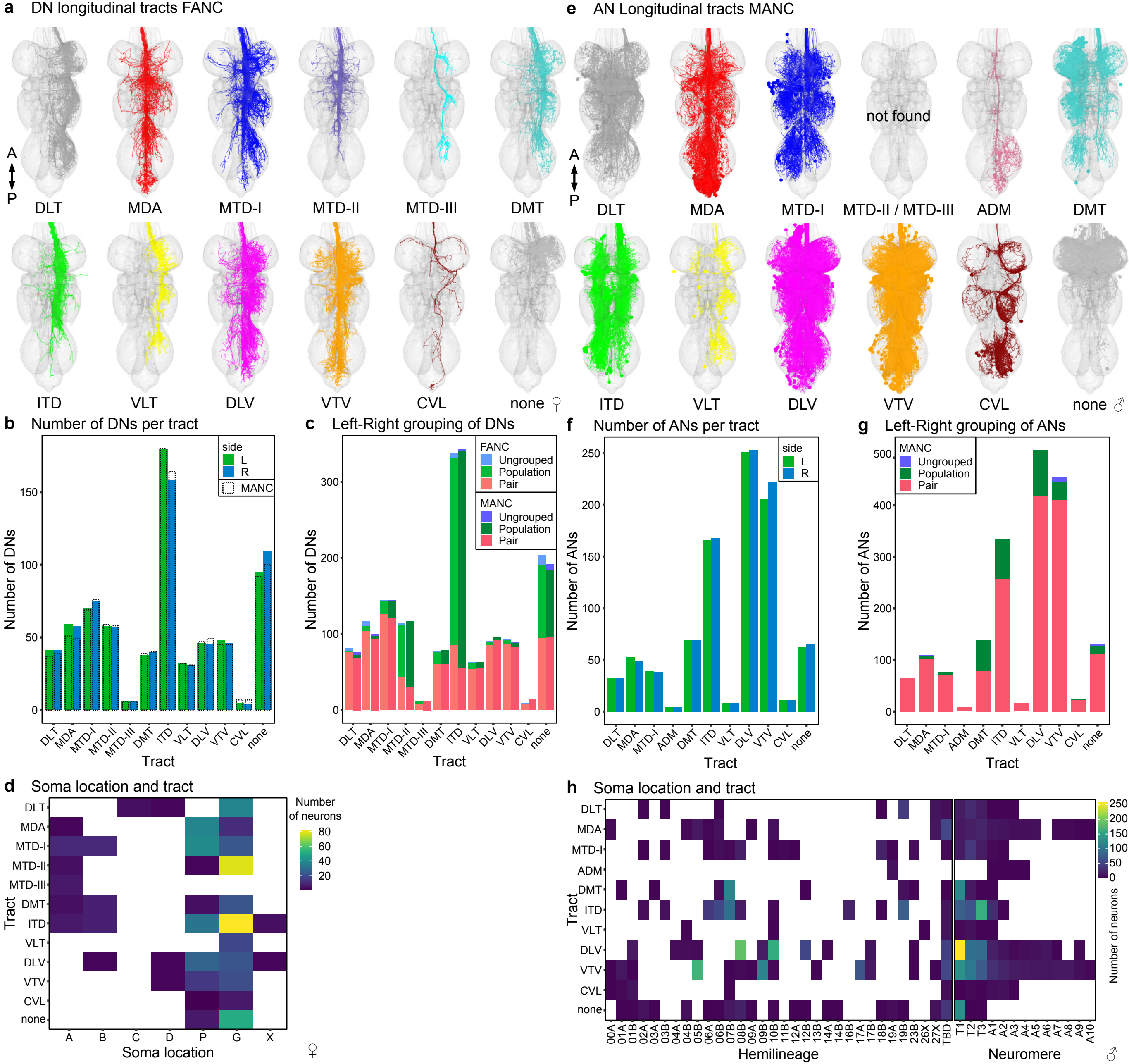

a DN longitudinal tracts FANC
A
P
DLT
MDA
MTD-I
MTD-II
MTD-III
DMT
ITD
VLT
DLV
VTV
CVL
none ♀
e AN Longitudinal tracts MANC
not found
DLT
MDA
MTD-I
MTD-II / MTD-III
ADM
DMT
ITD
VLT
DLV
VTV
CVL
none ♂
b Number of DNs per tract
side
L
R
MANC
Number of DNs
Tract
c Left-Right grouping of DNs
FANC
Ungrouped
Population
Pair
MANC
Ungrouped
Population
Pair
Number of DNs
Tract
f Number of ANs per tract
side
L
R
Number of ANs
Tract
g Left-Right grouping of ANs
MANC
Ungrouped
Population
Pair
Number of ANs
Tract
d Soma location and tract
Number of neurons
Tract
Soma location
A
B
C
D
P
G
X
♀
h Soma location and tract
Tract
Hemilineage
Neuromere
Number of neurons
♂
