# Supplementary material for: Comparative connectomics of the descending and ascending neurons of the Drosophila nervous system: stereotypy and sexual dimorphism
Source: bioRxiv. 2024 Jun 28:2024.06.04.596633. Originally published 2024 Jun 6. Preprint. [Version 2] doi: 10.1101/2024.06.04.596633 (PMC11185702; doi:10.1101/2024.06.04.596633)
Supplement: Supplement 4 [file media-4.zip › Fig3-sensory_ranking_formatted600.pdf]

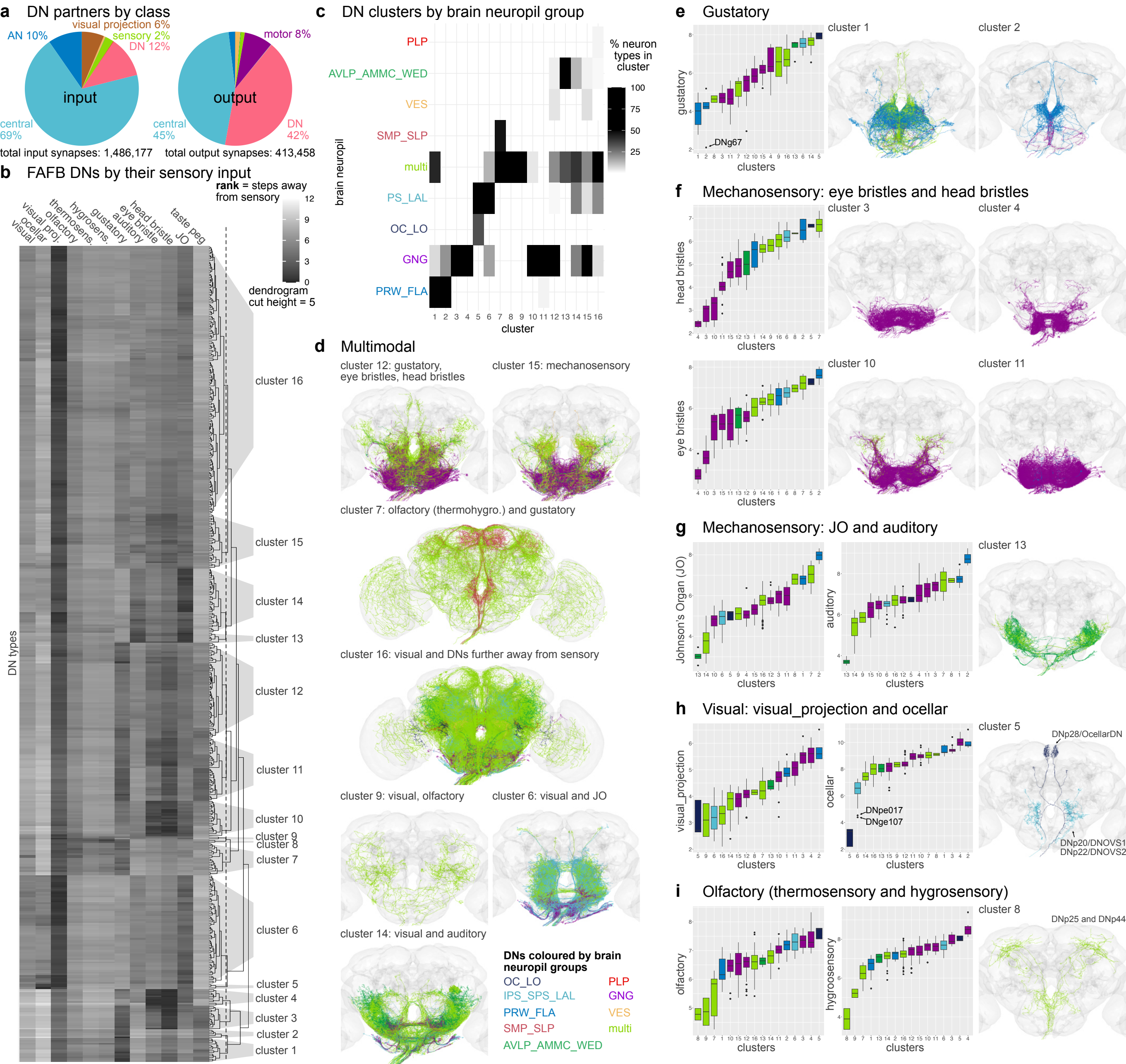
a DN partners by class
AN 10%
visual projection 6%
sensory 2%
DN 12%
input
central 69%
total input synapses: 1,486,177
motor 8%
output
central 45%
DN 42%
total output synapses: 413,458
b FAFB DNs by their sensory input
rank = steps away from sensory
dendrogram cut height = 5
visual
ocellar
visual proj.
olfactory
thermosens.
hygrosens.
gustatory
auditory
eye bristle
head bristle
JO
taste peg
DN types
cluster 16
cluster 15
cluster 14
cluster 13
cluster 12
cluster 11
cluster 10
cluster 9
cluster 8
cluster 7
cluster 6
cluster 5
cluster 4
cluster 3
cluster 2
cluster 1
c DN clusters by brain neuropil group
brain neuropil
PLP
AVLP_AMMC_WED
VES
SMP_SLP
multi
PS_LAL
OC_LO
GNG
PRW_FLA
% neuron types in cluster
cluster
d Multimodal
cluster 12: gustatory, eye bristles, head bristles
cluster 15: mechanosensory
cluster 7: olfactory (thermohygro.) and gustatory
cluster 16: visual and DNs further away from sensory
cluster 9: visual, olfactory
cluster 6: visual and JO
cluster 14: visual and auditory
DNs coloured by brain neuropil groups
OC_LO
IPS_SPS_LAL
PRW_FLA
SMP_SLP
AVLP_AMMC_WED
PLP
GNG
VES
multi
e Gustatory
gustatory
DNg67
clusters
cluster 1
cluster 2
f Mechanosensory: eye bristles and head bristles
head bristles
eye bristles
clusters
cluster 3
cluster 4
cluster 10
cluster 11
g Mechanosensory: JO and auditory
Johnson's Organ (JO)
auditory
clusters
cluster 13
h Visual: visual_projection and ocellar
visual_projection
ocellar
DNpe017
DNge107
clusters
cluster 5
DNp28/OcellarDN
DNp20/DNOVS1
DNp22/DNOVS2
i Olfactory (thermosensory and hygrosensory)
olfactory
hygrosensory
clusters
cluster 8
DNp25 and DNp44
