# Supplementary material for: Comparative connectomics of the descending and ascending neurons of the Drosophila nervous system: stereotypy and sexual dimorphism
Source: bioRxiv. 2024 Jun 28:2024.06.04.596633. Originally published 2024 Jun 6. Preprint. [Version 2] doi: 10.1101/2024.06.04.596633 (PMC11185702; doi:10.1101/2024.06.04.596633)
Supplement: Supplement 4 [file media-4.zip › Fig4-DNx02_formatted600.pdf]

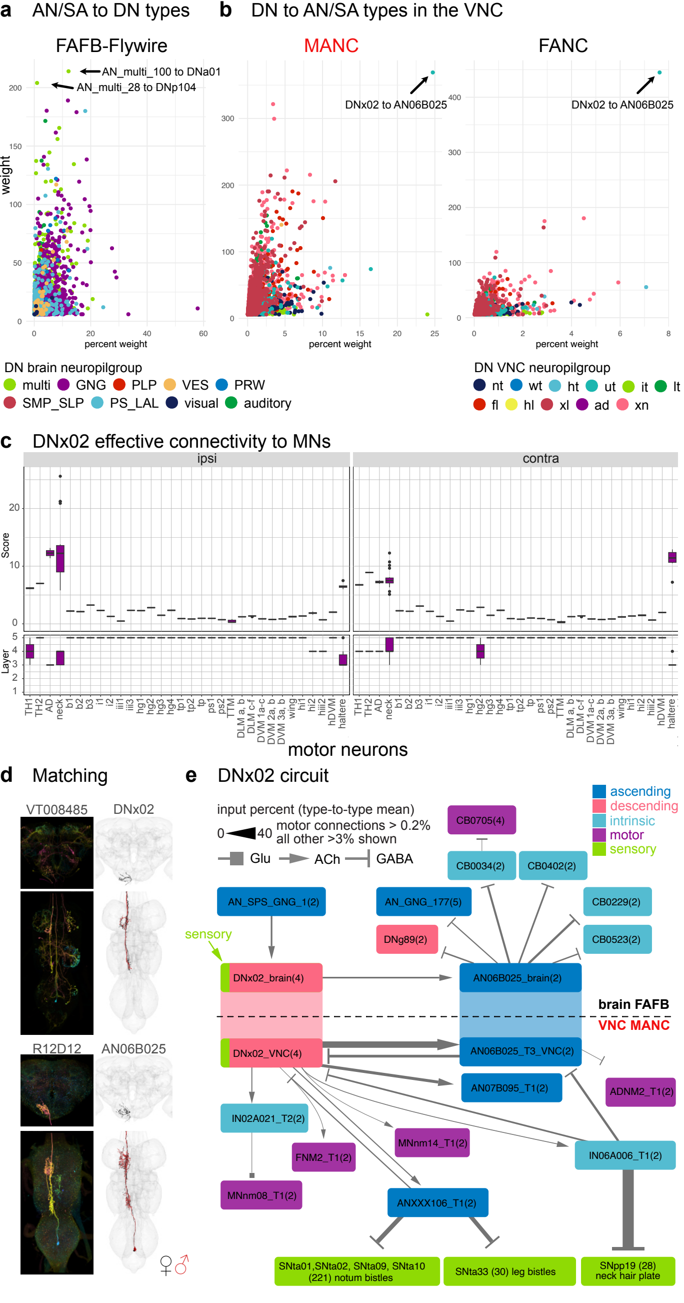

a AN/SA to DN types
FAFB-Flywire
AN_multi_100 to DNa01
AN_multi_28 to DNp104
weight
percent weight
0
20
40
60
50
100
150
200
b DN to AN/SA types in the VNC
MANC
DNx02 to AN06B025
0
5
10
15
20
25
100
200
300
percent weight
FANC
DNx02 to AN06B025
0
2
4
6
8
100
200
300
400
percent weight
DN brain neuropilgroup
multi GNG PLP VES PRW
SMP_SLP PS_LAL visual auditory
DN VNC neuropilgroup
nt wt ht ut it lt
fl hl xl ad xn
c DNx02 effective connectivity to MNs
ipsi
contra
Score
Layer
0
10
20
1
2
3
4
5
TH1 TH2 AD neck b1 b2 b3 i1 i2 iii1 iii3 hg1 hg2 hg3 hg4 tp1 tp2 tp ps1 ps2 TTM DLM a, b DLM c-f DVM 1a-c DVM 2a, b DVM 3a, b wing hi1 hi2 hiii2 hDVM haltere
motor neurons
d Matching
VT008485
DNx02
R12D12
AN06B025
♀♂
e DNx02 circuit
input percent (type-to-type mean)
0
40
motor connections > 0.2%
all other >3% shown
Glu
ACh
GABA
ascending
descending
intrinsic
motor
sensory
CB0705(4)
CB0034(2)
CB0402(2)
AN_SPS_GNG_1(2)
AN_GNG_177(5)
CB0229(2)
DNg89(2)
CB0523(2)
sensory
DNx02_brain(4)
AN06B025_brain(2)
brain FAFB
VNC MANC
DNx02_VNC(4)
AN06B025_T3_VNC(2)
AN07B095_T1(2)
ADNM2_T1(2)
IN02A021_T2(2)
MNnm14_T1(2)
FNM2_T1(2)
IN06A006_T1(2)
MNnm08_T1(2)
ANXXX106_T1(2)
SNta01,SNta02, SNta09, SNta10 (221) notum bistles
SNta33 (30) leg bistles
SNpp19 (28) neck hair plate
