# Supplementary material for: Comparative connectomics of the descending and ascending neurons of the Drosophila nervous system: stereotypy and sexual dimorphism
Source: bioRxiv. 2024 Jun 28:2024.06.04.596633. Originally published 2024 Jun 6. Preprint. [Version 2] doi: 10.1101/2024.06.04.596633 (PMC11185702; doi:10.1101/2024.06.04.596633)
Supplement: Supplement 4 [file media-4.zip › Fig5-stereotypy_formatted600.pdf]

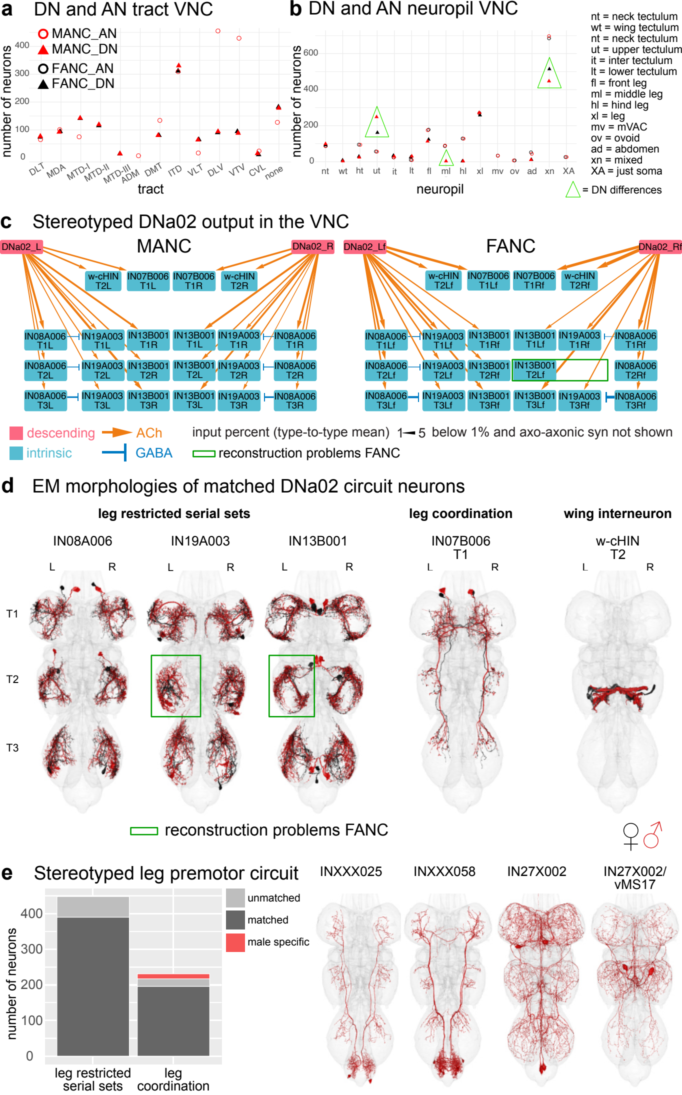
a DN and AN tract VNC
MANC_AN
MANC_DN
FANC_AN
FANC_DN
number of neurons
0
100
200
300
400
DLT
MDA
MTD-I
MTD-II
MTD-III
ADM
DMT
ITD
VLT
DLV
VTV
CVL
none
tract
b DN and AN neuropil VNC
number of neurons
0
200
400
600
nt
wt
ht
ut
it
lt
fl
ml
hl
xl
mv
ov
ad
xn
XA
neuropil
nt = neck tectulum
wt = wing tectulum
nt = neck tectulum
ut = upper tectulum
it = inter tectulum
lt = lower tectulum
fl = front leg
ml = middle leg
hl = hind leg
xl = leg
mv = mVAC
ov = ovoid
ad = abdomen
xn = mixed
XA = just soma
= DN differences
c Stereotyped DNa02 output in the VNC
MANC
DNa02_L
DNa02_R
w-cHIN T2L
IN07B006 T1L
IN07B006 T1R
w-cHIN T2R
IN08A006 T1L
IN19A003 T1L
IN13B001 T1R
IN13B001 T1L
IN19A003 T1R
IN08A006 T1R
IN08A006 T2L
IN19A003 T2L
IN13B001 T2R
IN13B001 T2L
IN19A003 T2R
IN08A006 T2R
IN08A006 T3L
IN19A003 T3L
IN13B001 T3R
IN13B001 T3L
IN19A003 T3R
IN08A006 T3R
FANC
DNa02_Lf
DNa02_Rf
w-cHIN T2Lf
IN07B006 T1Lf
IN07B006 T1Rf
w-cHIN T2Rf
IN08A006 T1Lf
IN19A003 T1Lf
IN13B001 T1Rf
IN13B001 T1Lf
IN19A003 T1Rf
IN08A006 T1Rf
IN08A006 T2Lf
IN19A003 T2Lf
IN13B001 T2Rf
IN13B001 T2Lf
IN08A006 T2Rf
IN08A006 T3Lf
IN19A003 T3Lf
IN13B001 T3Rf
IN13B001 T3Lf
IN19A003 T3Rf
IN08A006 T3Rf
descending
intrinsic
ACh
GABA
input percent (type-to-type mean) 1 5 below 1% and axo-axonic syn not shown
reconstruction problems FANC
d EM morphologies of matched DNa02 circuit neurons
leg restricted serial sets
leg coordination
wing interneuron
IN08A006
IN19A003
IN13B001
IN07B006 T1
w-cHIN T2
L
R
T1
T2
T3
reconstruction problems FANC
♀ ♂
e Stereotyped leg premotor circuit
number of neurons
0
100
200
300
400
leg restricted serial sets
leg coordination
unmatched
matched
male specific
INXXX025
INXXX058
IN27X002
IN27X002/ vMS17
