# Supplementary material for: Comparative connectomics of the descending and ascending neurons of the Drosophila nervous system: stereotypy and sexual dimorphism
Source: bioRxiv. 2024 Jun 28:2024.06.04.596633. Originally published 2024 Jun 6. Preprint. [Version 2] doi: 10.1101/2024.06.04.596633 (PMC11185702; doi:10.1101/2024.06.04.596633)
Supplement: Supplement 4 [file media-4.zip › Fig7-dimorphic_DNs_formatted1200.pdf]

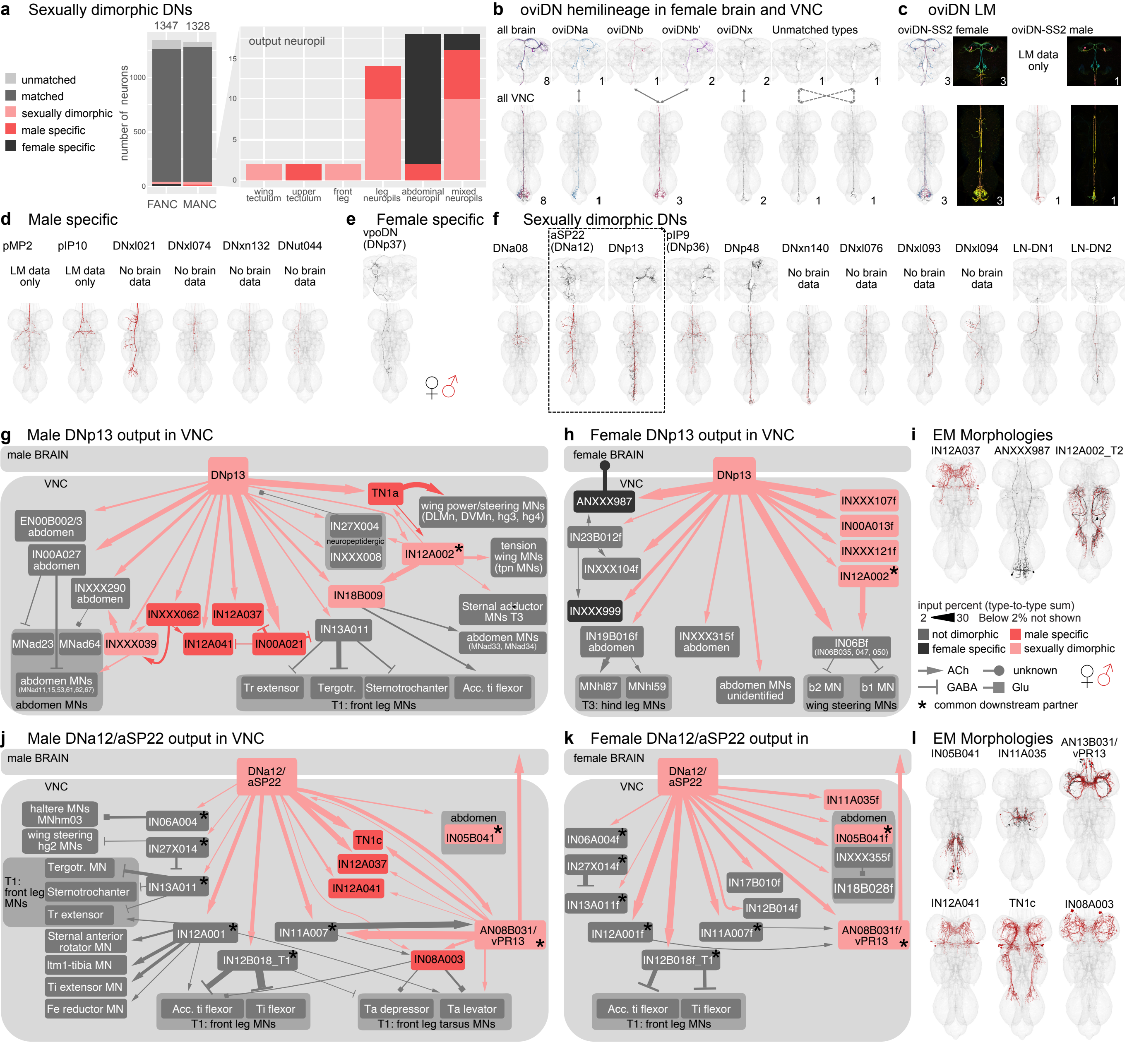

a Sexually dimorphic DNs
1347 1328
unmatched
matched
sexually dimorphic
male specific
female specific
number of neurons
FANC MANC
output neuropil
wing tectulum
upper tectulum
front leg
leg neuropils
abdominal neuropil
mixed neuropils
b oviDN hemilineage in female brain and VNC
all brain
oviDNa
oviDNb
oviDNb'
oviDNx
Unmatched types
all VNC
c oviDN LM
oviDN-SS2 female
oviDN-SS2 male
LM data only
d Male specific
pMP2
pIP10
DNxl021
DNxl074
DNxn132
DNut044
LM data only
No brain data
e Female specific
vpoDN (DNp37)
f Sexually dimorphic DNs
DNa08
aSP22 (DNa12)
DNp13
pIP9 (DNp36)
DNp48
DNxn140
DNxl076
DNxl093
DNxl094
LN-DN1
LN-DN2
No brain data
g Male DNp13 output in VNC
male BRAIN
VNC
DNp13
TN1a
wing power/steering MNs (DLMn, DVMn, hg3, hg4)
EN00B002/3 abdomen
IN27X004
neuropeptidergic
INXXX008
IN12A002*
tension wing MNs (tpn MNs)
IN00A027 abdomen
INXXX290 abdomen
IN18B009
Sternal adductor MNs T3
INXXX062
IN12A037
abdomen MNs (MNad33, MNad34)
MNad23
MNad64
INXXX039
IN12A041
IN00A021
IN13A011
abdomen MNs (MNad11,15,53,61,62,67)
abdomen MNs
Tr extensor
Tergotr.
Sternotrochanter
Acc. ti flexor
T1: front leg MNs
h Female DNp13 output in VNC
female BRAIN
VNC
DNp13
ANXXX987
INXXX107f
IN00A013f
IN23B012f
INXXX121f
INXXX104f
IN12A002*
INXXX999
IN19B016f abdomen
INXXX315f abdomen
IN06Bf (IN06B035, 047, 050)
MNhl87
MNhl59
T3: hind leg MNs
abdomen MNs unidentified
b2 MN
b1 MN
wing steering MNs
i EM Morphologies
IN12A037
ANXXX987
IN12A002_T2
input percent (type-to-type sum)
2 30 Below 2% not shown
not dimorphic
male specific
female specific
sexually dimorphic
ACh
unknown
GABA
Glu
* common downstream partner
j Male DNa12/aSP22 output in VNC
male BRAIN
VNC
DNa12/ aSP22
haltere MNs MNhm03
IN06A004*
wing steering hg2 MNs
IN27X014*
abdomen
IN05B041*
TN1c
IN12A037
IN12A041
T1: front leg MNs
Tergotr. MN
Sternotrochanter
Tr extensor
IN13A011*
Sternal anterior rotator MN
ltm1-tibia MN
Ti extensor MN
Fe reductor MN
IN12A001*
IN11A007*
AN08B031/ vPR13*
IN12B018_T1*
IN08A003
Acc. ti flexor
Ti flexor
T1: front leg MNs
Ta depressor
Ta levator
T1: front leg tarsus MNs
k Female DNa12/aSP22 output in
female BRAIN
VNC
DNa12/ aSP22
IN11A035f
abdomen
IN05B041f*
INXXX355f
IN18B028f
IN06A004f*
IN27X014f*
IN13A011f*
IN17B010f
IN12B014f
IN12A001f*
IN11A007f*
AN08B031f/ vPR13*
IN12B018f_T1*
Acc. ti flexor
Ti flexor
T1: front leg MNs
l EM Morphologies
IN05B041
IN11A035
AN13B031/ vPR13
IN12A041
TN1c
IN08A003
