# Supplementary figures and images for: Comparative connectomics of the descending and ascending neurons of the Drosophila nervous system: stereotypy and sexual dimorphism
Source: bioRxiv. 2024 Jun 28:2024.06.04.596633. Originally published 2024 Jun 6. Preprint. [Version 2] doi: 10.1101/2024.06.04.596633 (PMC11185702; doi:10.1101/2024.06.04.596633)

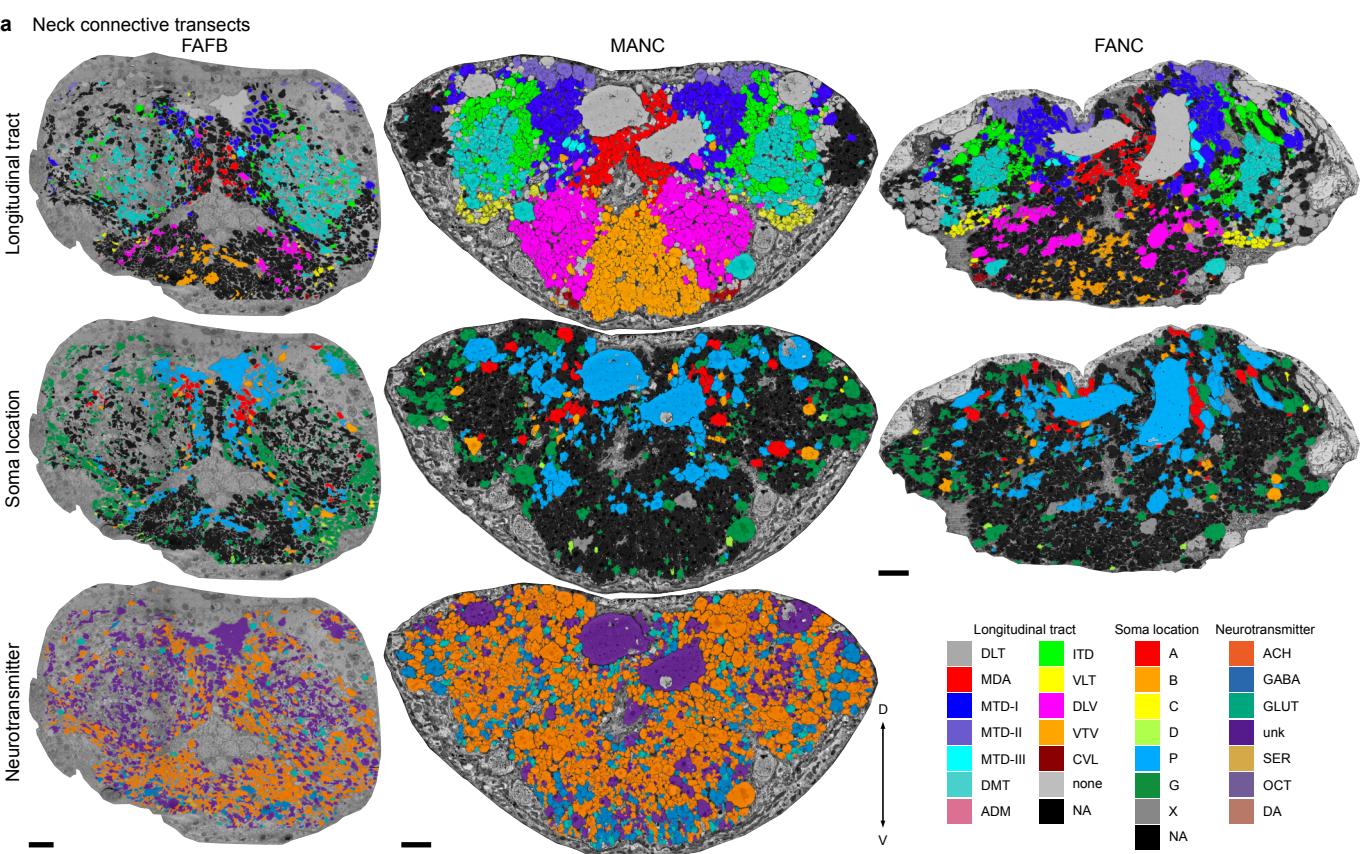

**b** Neuron counts

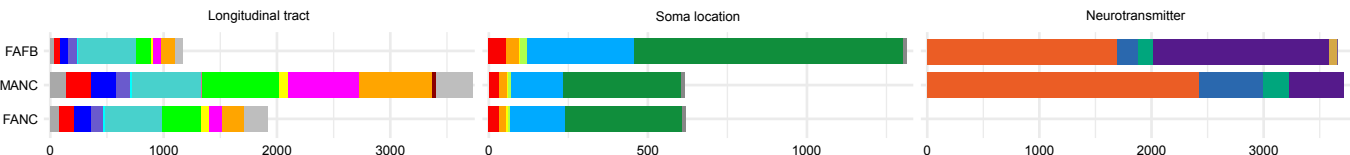

Supplement: Supplement 4 [file media-4.zip › Extended_Data_Fig1_formatted600.pdf]

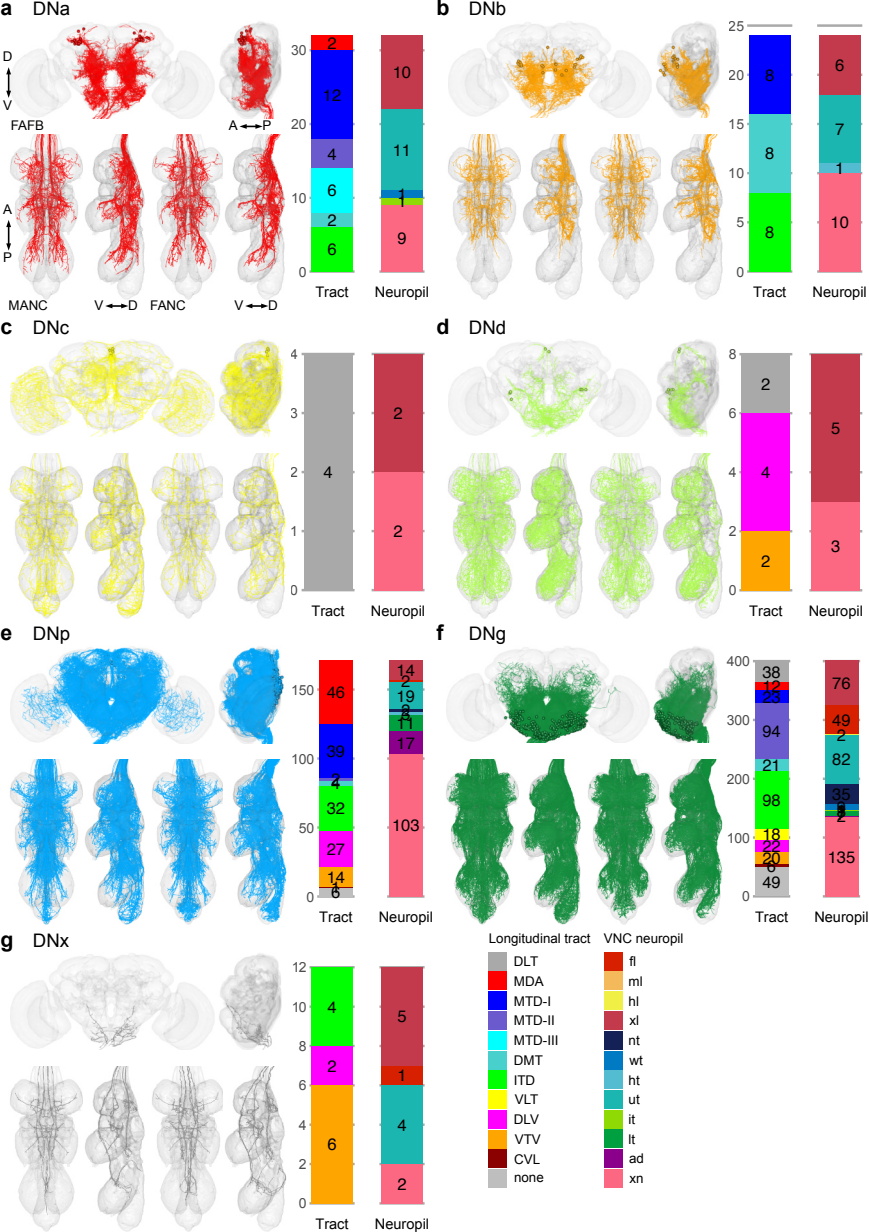

Supplement: Supplement 4 [file media-4.zip › Extended_Data_Fig3_formatted600.pdf]

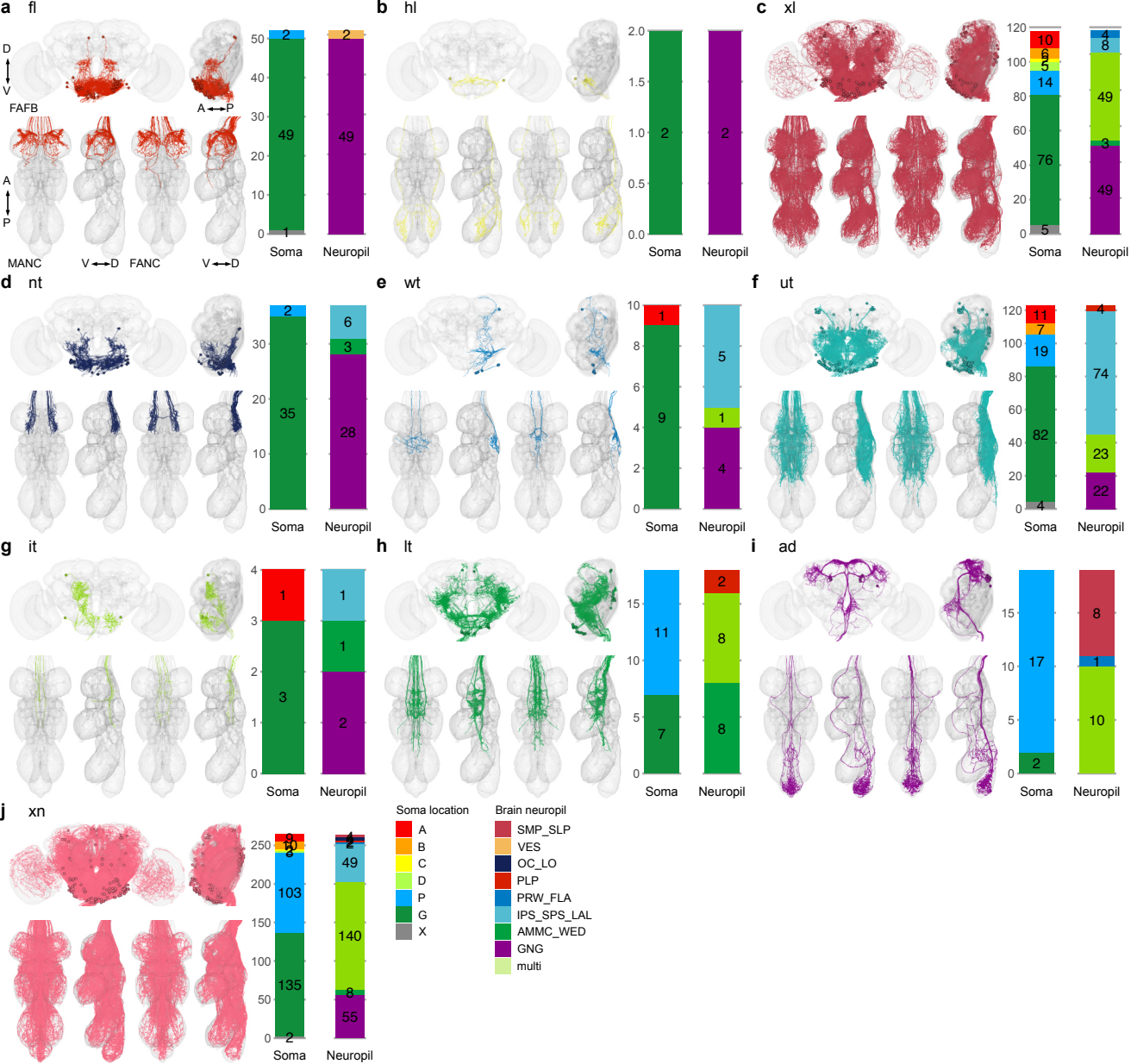

Supplement: Supplement 4 [file media-4.zip › Extended_Data_Fig5_formatted600.pdf]

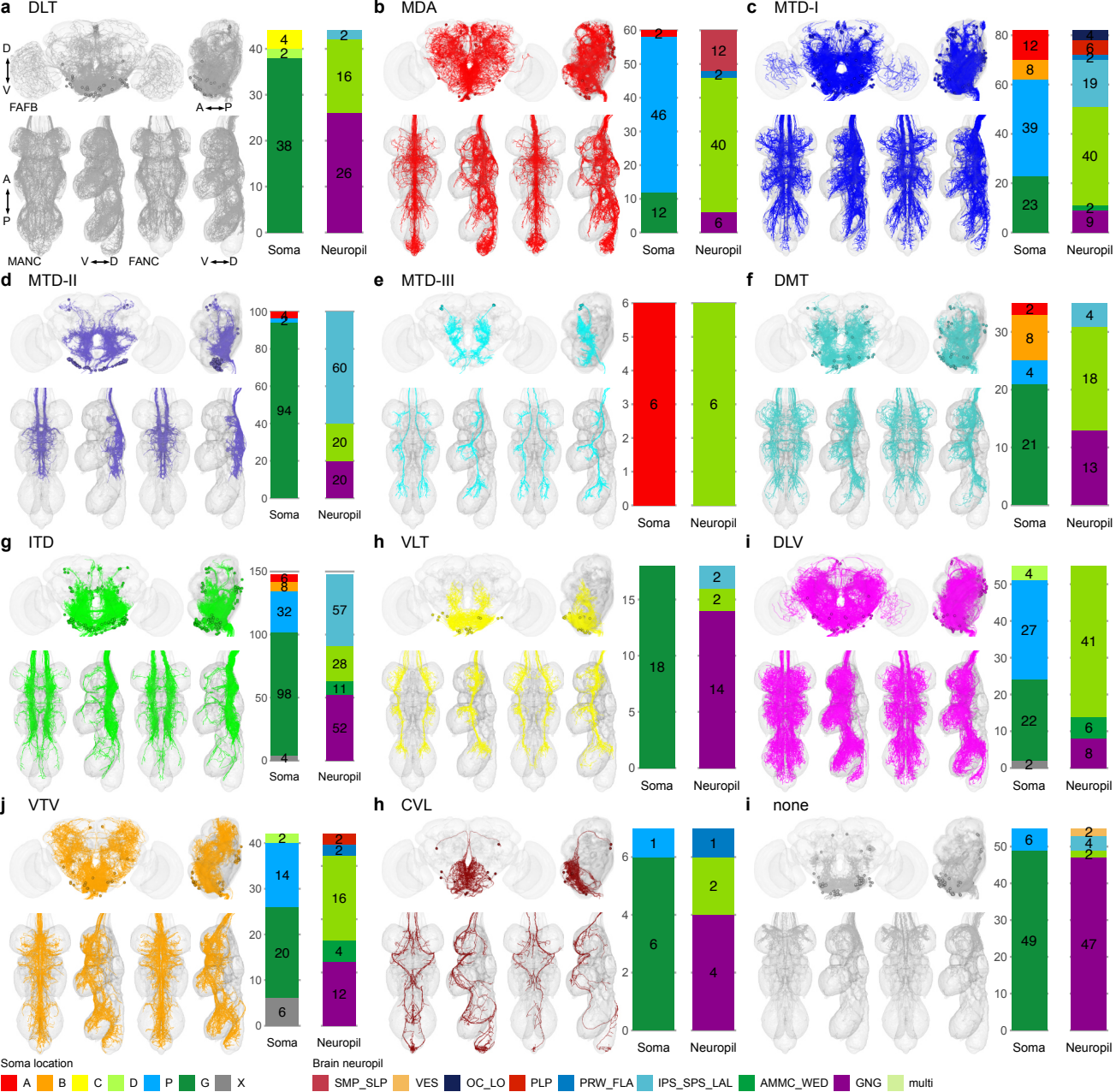

Supplement: Supplement 4 [file media-4.zip › Extended_Data_Fig6_formatted600.pdf]

**a** FAFB DNs by brain neuropil groups

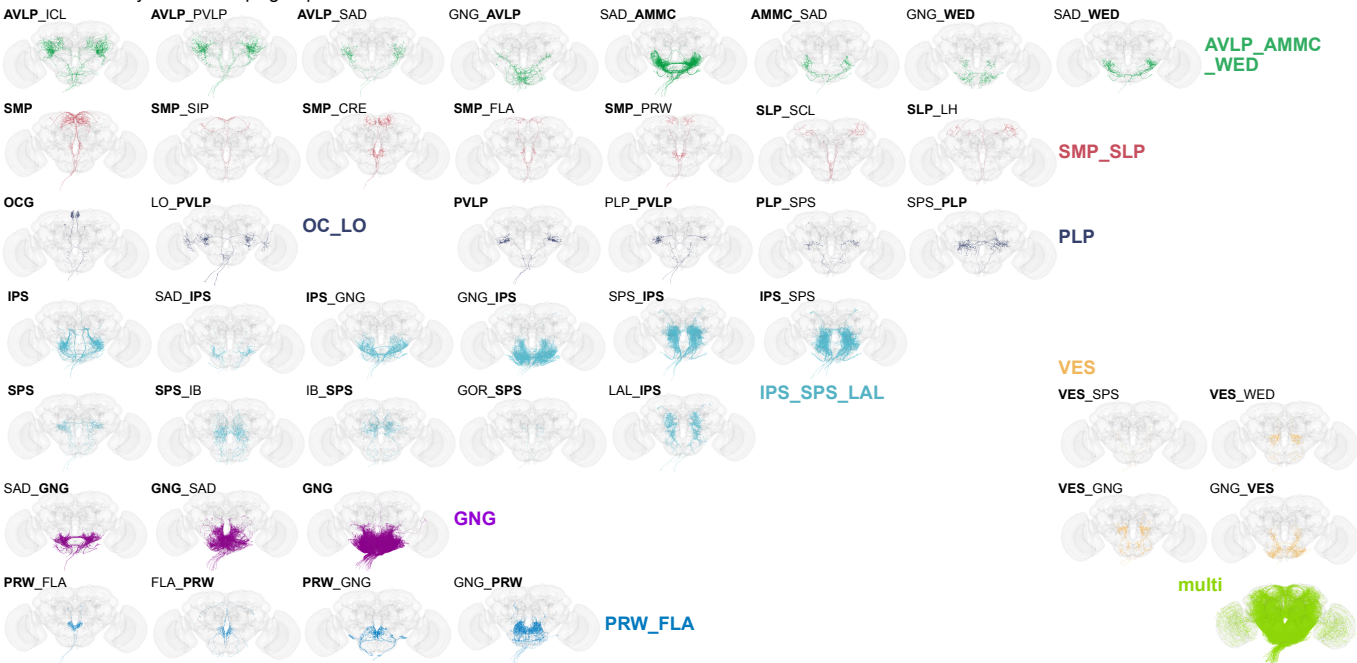

**b** FAFB ANs by brain neuropil groups

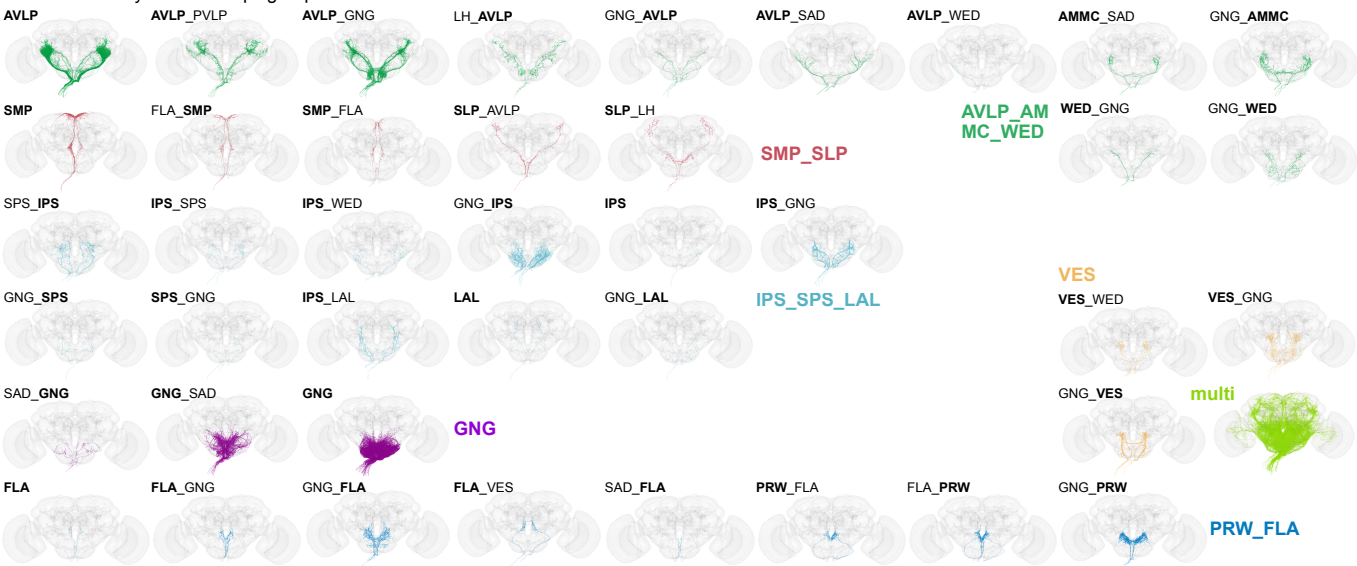

Supplement: Supplement 4 [file media-4.zip › Extended_Data_Fig8_formatted1200.pdf]

# a

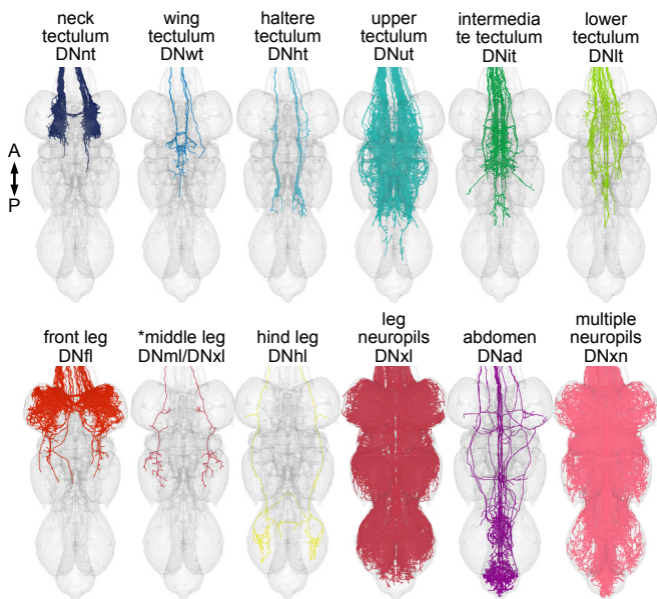

# b

VNC neuropil innervation of matched DNs and ANs

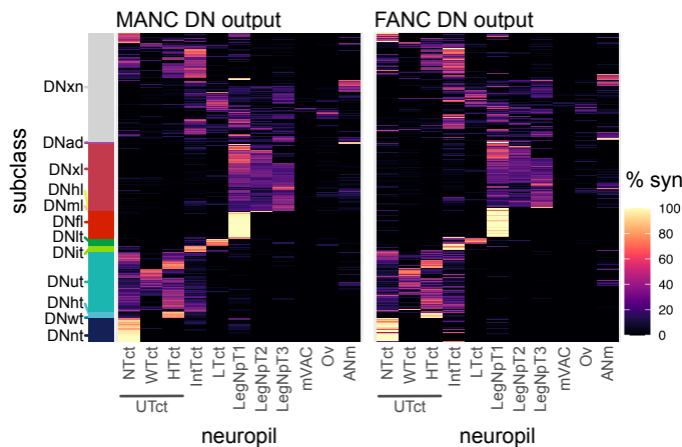

Supplement: Supplement 4 [file media-4.zip › Extended_Data_Fig9_formatted600.pdf]

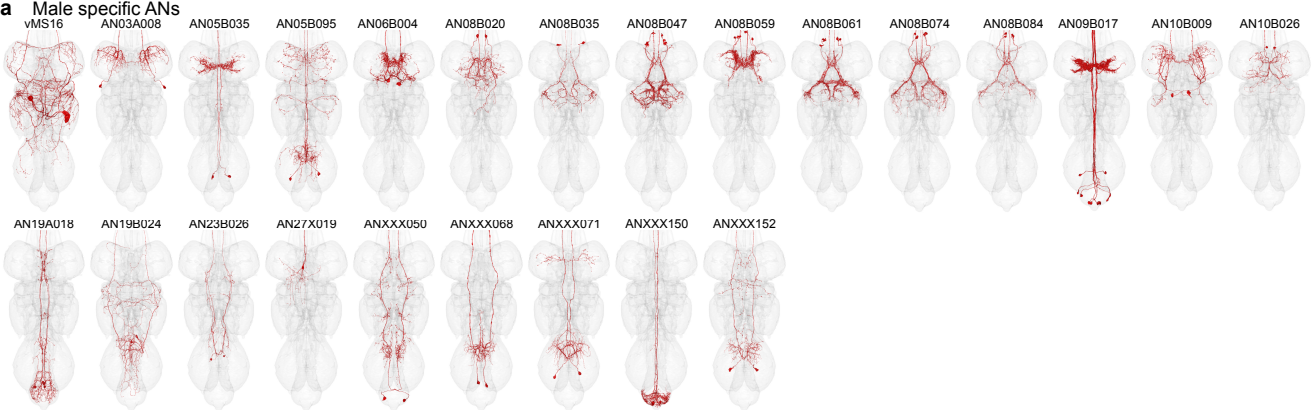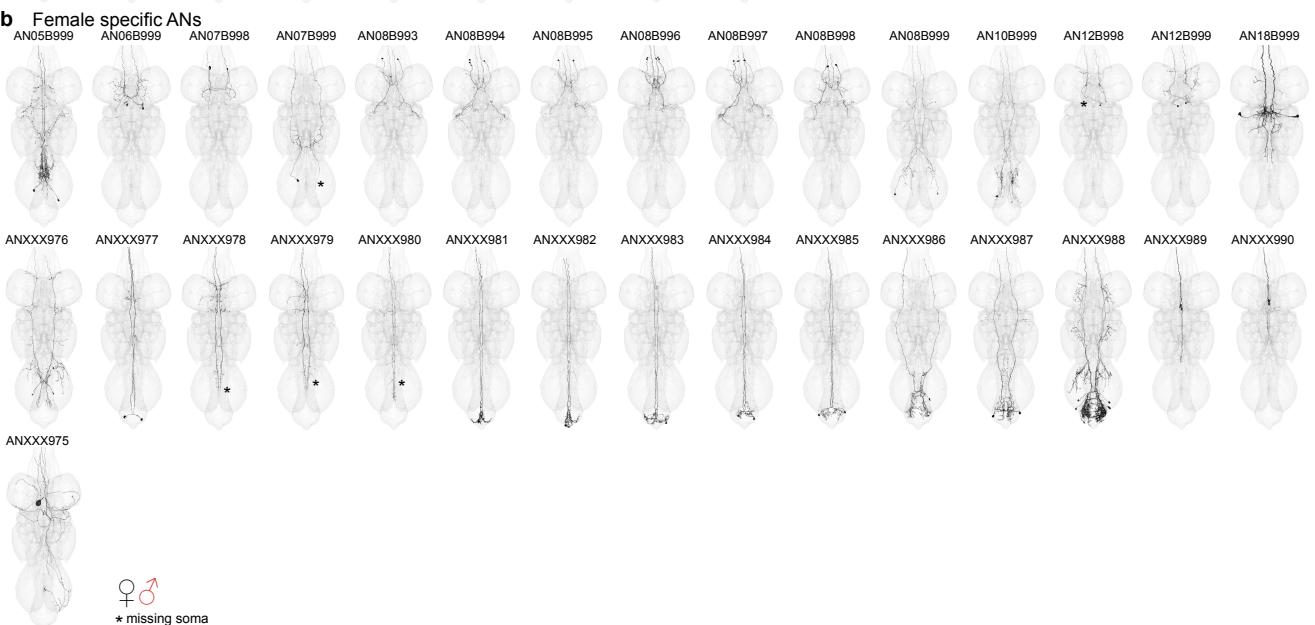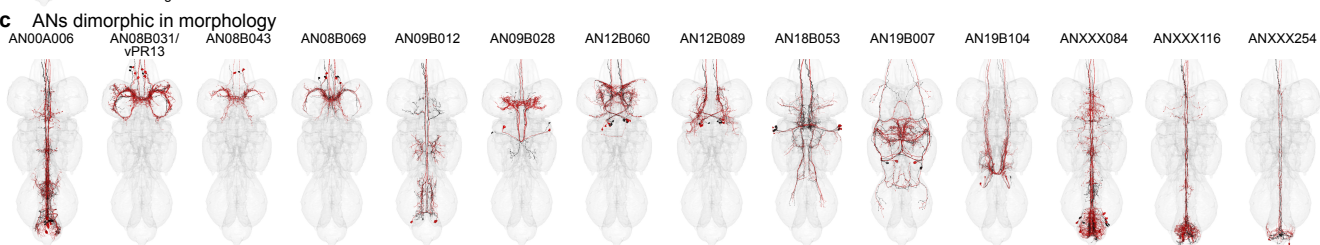

Supplement: Supplement 4 [file media-4.zip › Extended_Data_Fig11_formatted1200.pdf]

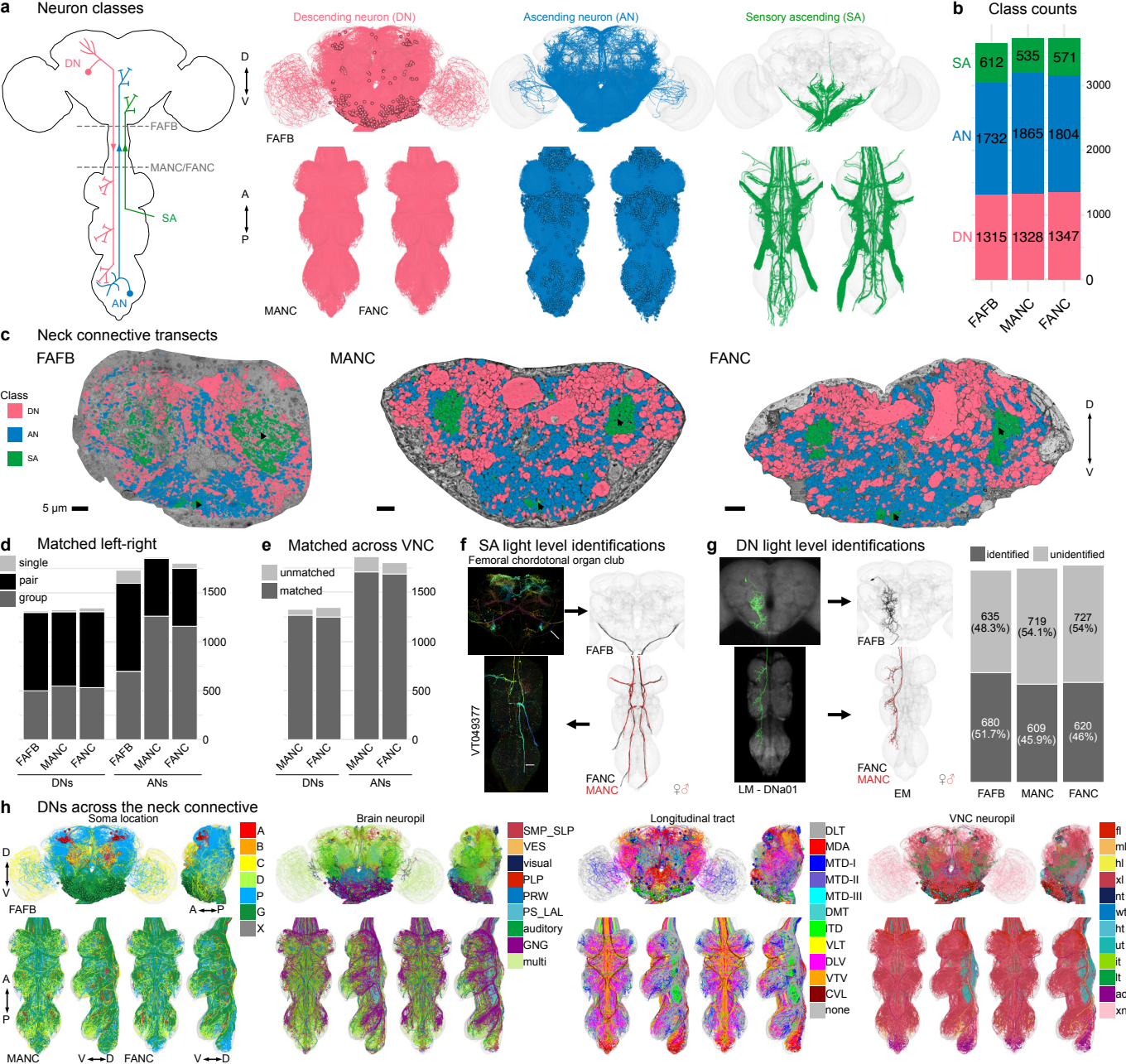

Supplement: Supplement 4 [file media-4.zip › Fig1-Overview_formatted600.pdf]

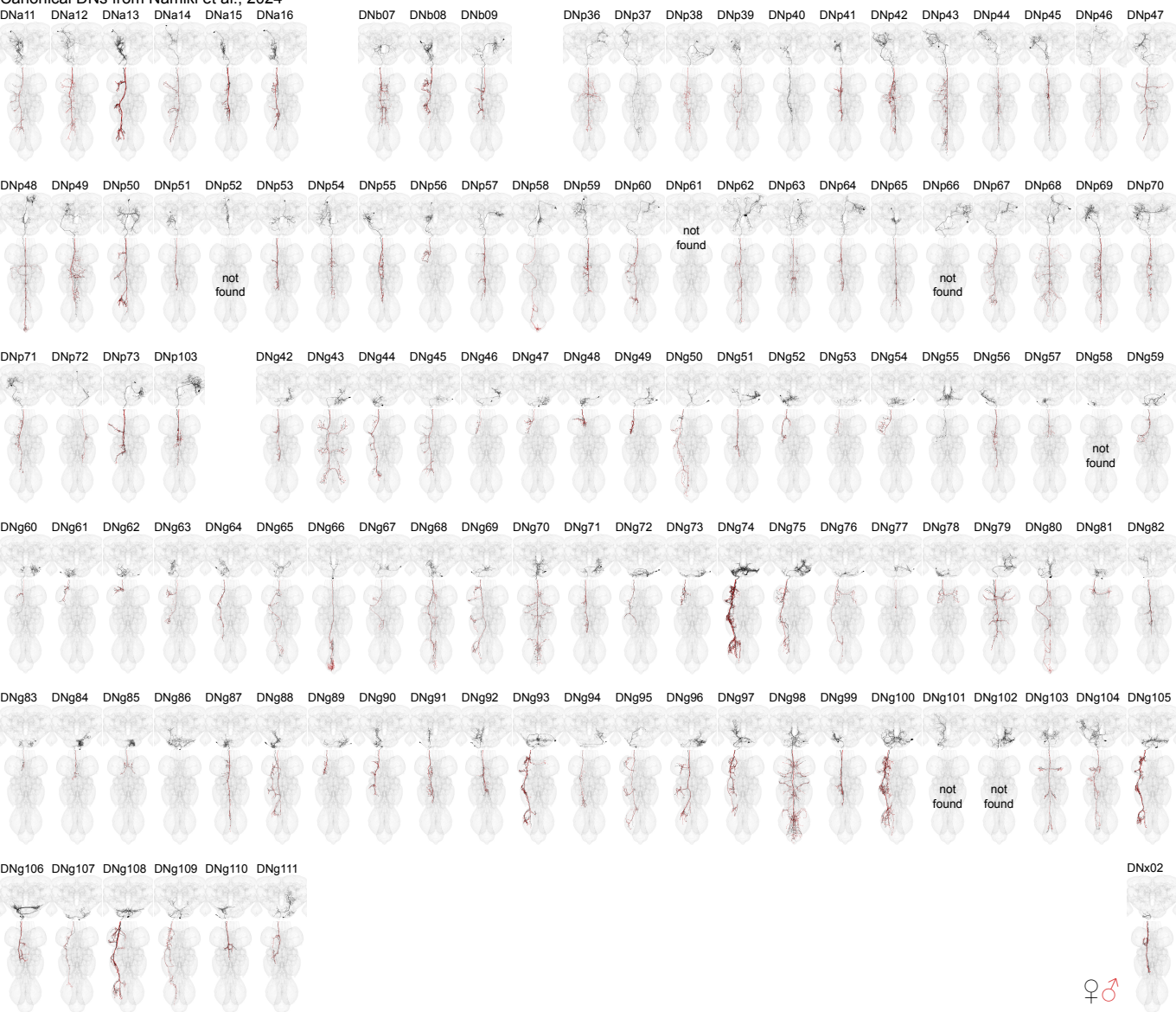

Supplement: Supplement 4 [file media-4.zip › Fig2-Namiki2_DNs_formatted_1200.pdf]

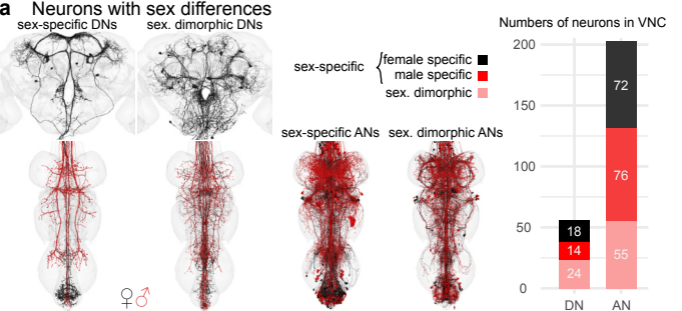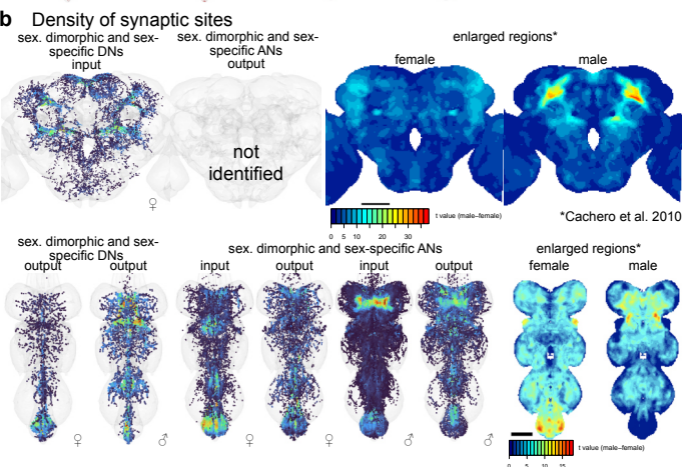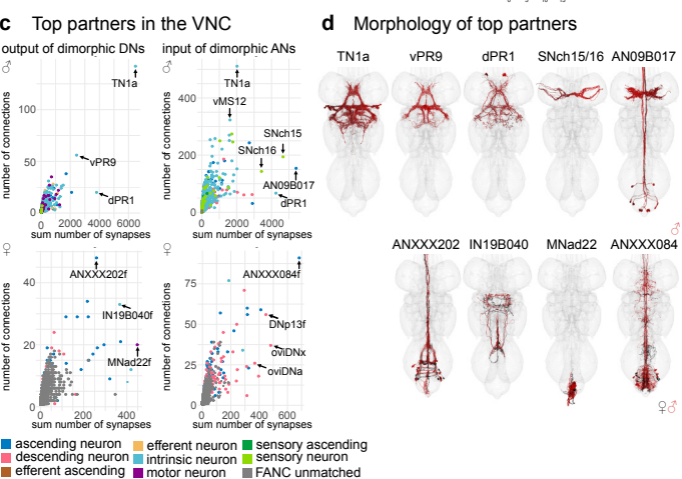

Supplement: Supplement 4 [file media-4.zip › Fig6-dimorphism_formatted600.pdf]
