# Supplementary material for: Comparative connectomics of the descending and ascending neurons of the Drosophila nervous system: stereotypy and sexual dimorphism
Source: bioRxiv. 2024 Jun 28:2024.06.04.596633. Originally published 2024 Jun 6. Preprint. [Version 2] doi: 10.1101/2024.06.04.596633 (PMC11185702; doi:10.1101/2024.06.04.596633)

Canonical DNs from Namiki et al., 2018

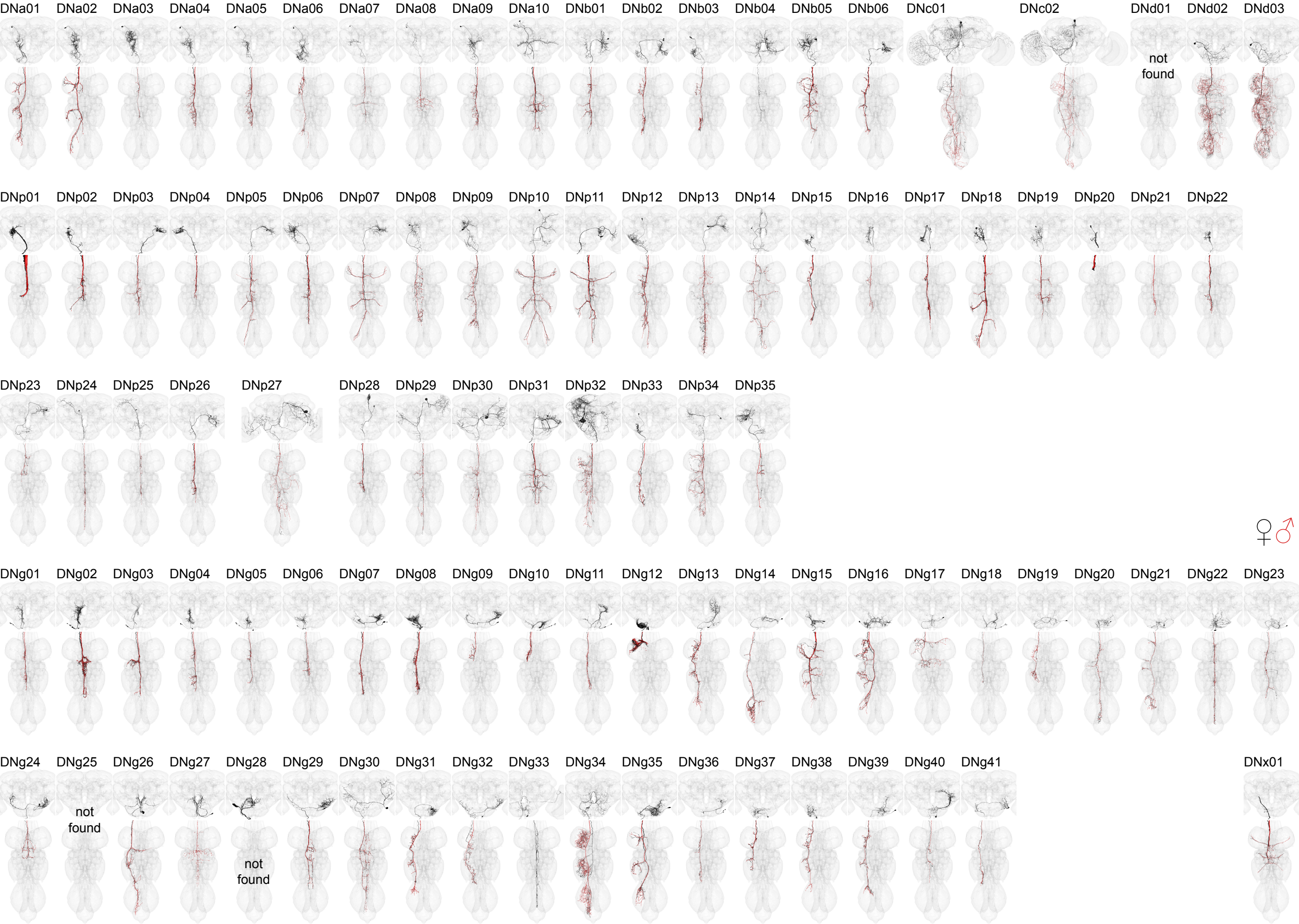

Supplement: Supplement 4 [file Extended_Data_Fig7_Namiki_DNs_formatted1200.pdf › Extended_Data_Fig7_Namiki_DNs_formatted1200.pdf]
