# Supplementary material for: Comparative connectomics of the descending and ascending neurons of the Drosophila nervous system: stereotypy and sexual dimorphism
Source: bioRxiv. 2024 Jun 6:2024.06.04.596633. Preprint. [Version 1] doi: 10.1101/2024.06.04.596633 (PMC11185702; doi:10.1101/2024.06.04.596633)
Supplement: Supplementary file 1 [file NIHPP2024.06.04.596633v1-supplement-4.pdf]

bioRxiv preprint doi: https://doi.org/10.1101/2024.06.04.596633; this version posted June 6, 2024. The copyright holder for this preprint (which was not certified by peer review) is the author/funder, who has granted bioRxiv a license to display the preprint in perpetuity. It is made available under aCC-BY 4.0 International license.

Supplemental figures

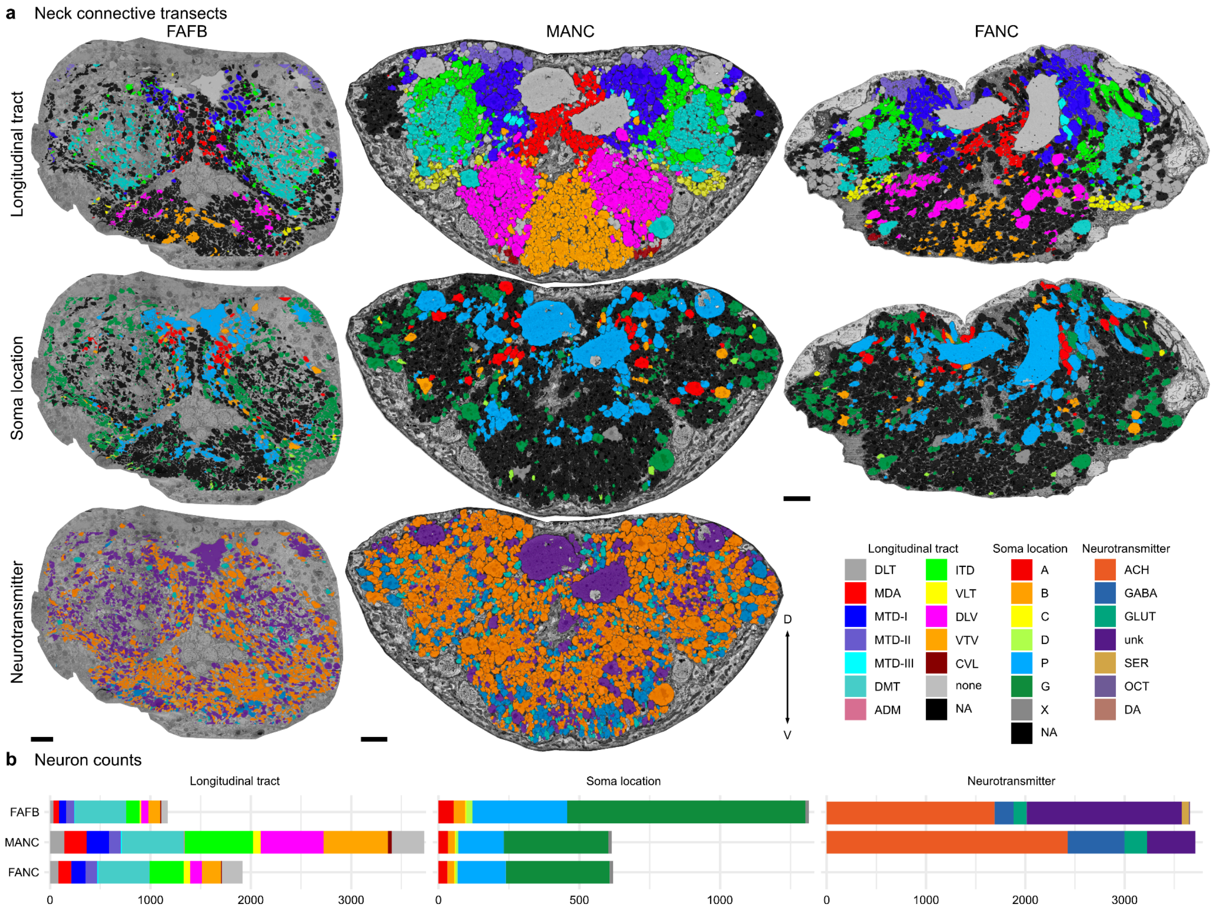

Fig.1 - figure supplemental 1: Cross section (frontal) of the neck connective in the three datasets. a, All neurons in the neck connective colour coded by their longitudinal tract, soma location or predicted neurotransmitter. **b**, Number of neurons by longitudinal tract, soma location or neurotransmitter in the three datasets. Neurotransmitter predictions are not yet available in FANC.

bioRxiv preprint doi: https://doi.org/10.1101/2024.06.04.596633; this version posted June 6, 2024. The copyright holder for this preprint (which was not certified by peer review) is the author/funder, who has granted bioRxiv a license to display the preprint in perpetuity. It is made available under aCC-BY 4.0 International license.

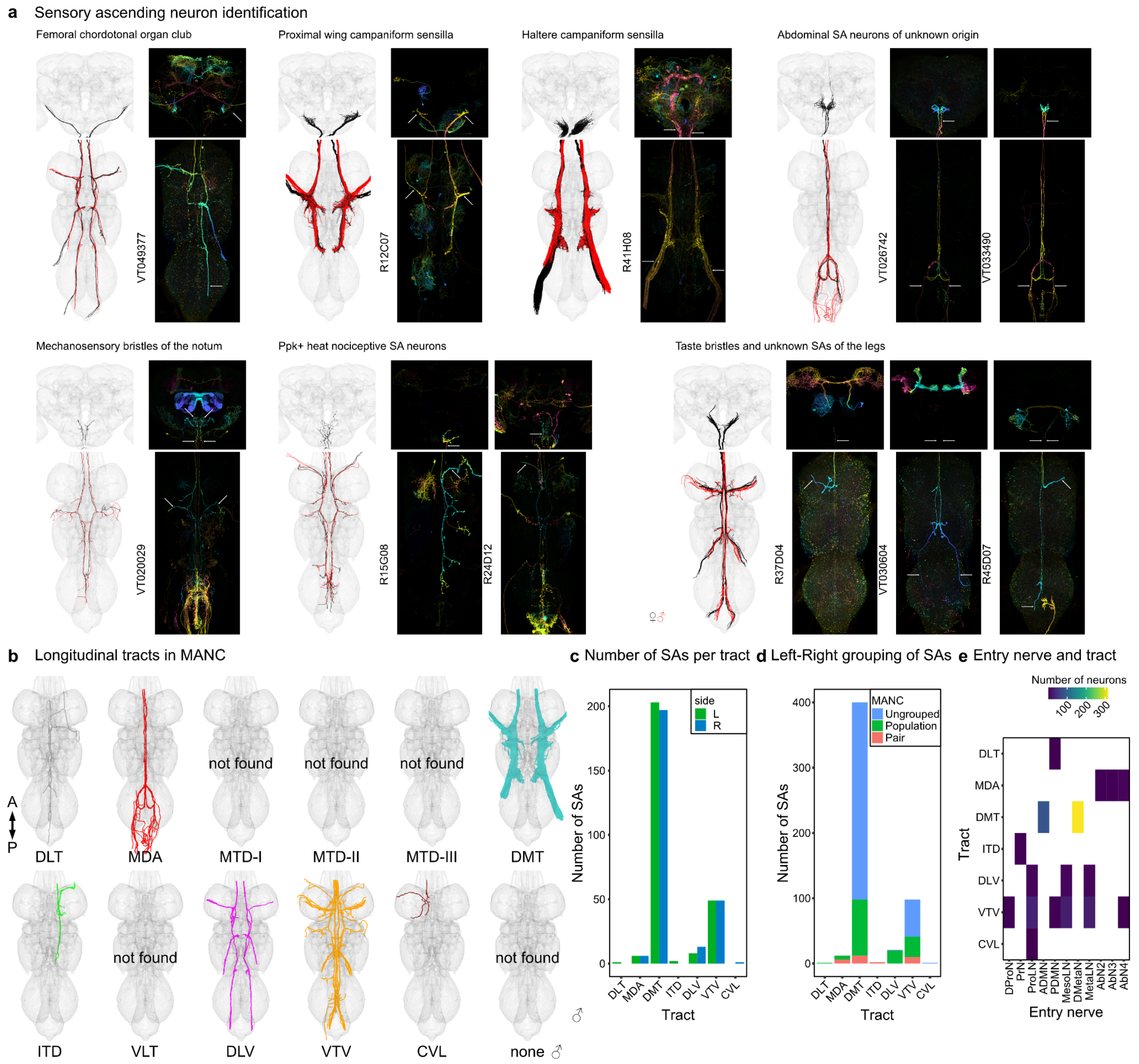

Fig.1 - figure supplemental 2: Sensory ascending neurons. a,Morphology of sensory ascending neurons identified in the three EM volumes. In black the EM morphology of DNs from female datasets (FAFB, FANC) in red from the male dataset (MANC). Next to them the LM images that allowed a grouping into sensory subclasses. **b**, Tract-based analysis of sensory ascending neurons in MANC. None of the SAs project along the MTD, or VLT tract. **c**, Number of SAs in each tract. **d**, Number of SA grouped into pairs or populations. **e**, Correlation of entry nerve to tract membership for MANC SAs (Marin et al. 2023).

bioRxiv preprint doi: https://doi.org/10.1101/2024.06.04.596633; this version posted June 6, 2024. The copyright holder for this preprint (which was not certified by peer review) is the author/funder, who has granted bioRxiv a license to display the preprint in perpetuity. It is made available under aCC-BY 4.0 International license.

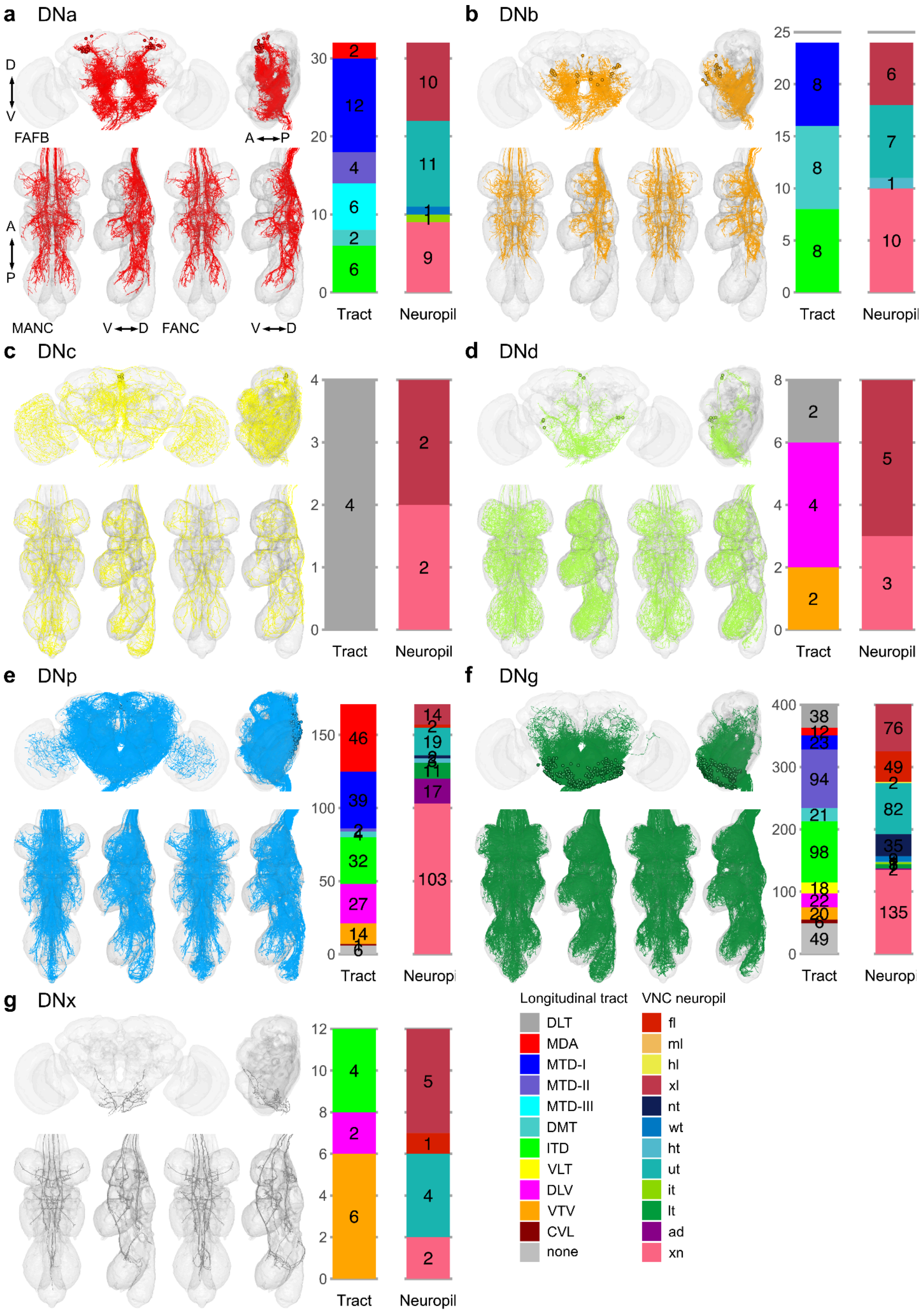

Fig.1 - figure supplemental 3: Morphology matched across the neck - soma. Morphology of LM matched DNs across the three datasets colour coded by the location of the cell body according to (Namiki et al. 2018). **a,** DNa neurons have an anterior dorsal soma; **b**, DNb an anterior ventral soma; **c**, DNc a soma in the pars intercerebralis; **d**, DNd a soma in an anterior outside cell cluster; **e**, DNp are one the posterior surface; **f**, DNg are located in the GNG and **g**, DNx are outside the brain. In each panel the top images show reconstruction in FAFB in anterior and lateral view; the two bottom left images show MANC and two bottom right FANC in ventral and lateral view, respectively. The bar charts represent the distribution of the VNC characteristics longitudinal tract and neuropil innervation for the neurons in each category for which the colour legend can be found at the bottom right.

bioRxiv preprint doi: https://doi.org/10.1101/2024.06.04.596633; this version posted June 6, 2024. The copyright holder for this preprint (which was not certified by peer review) is the author/funder, who has granted bioRxiv a license to display the preprint in perpetuity. It is made available under aCC-BY 4.0 International license.

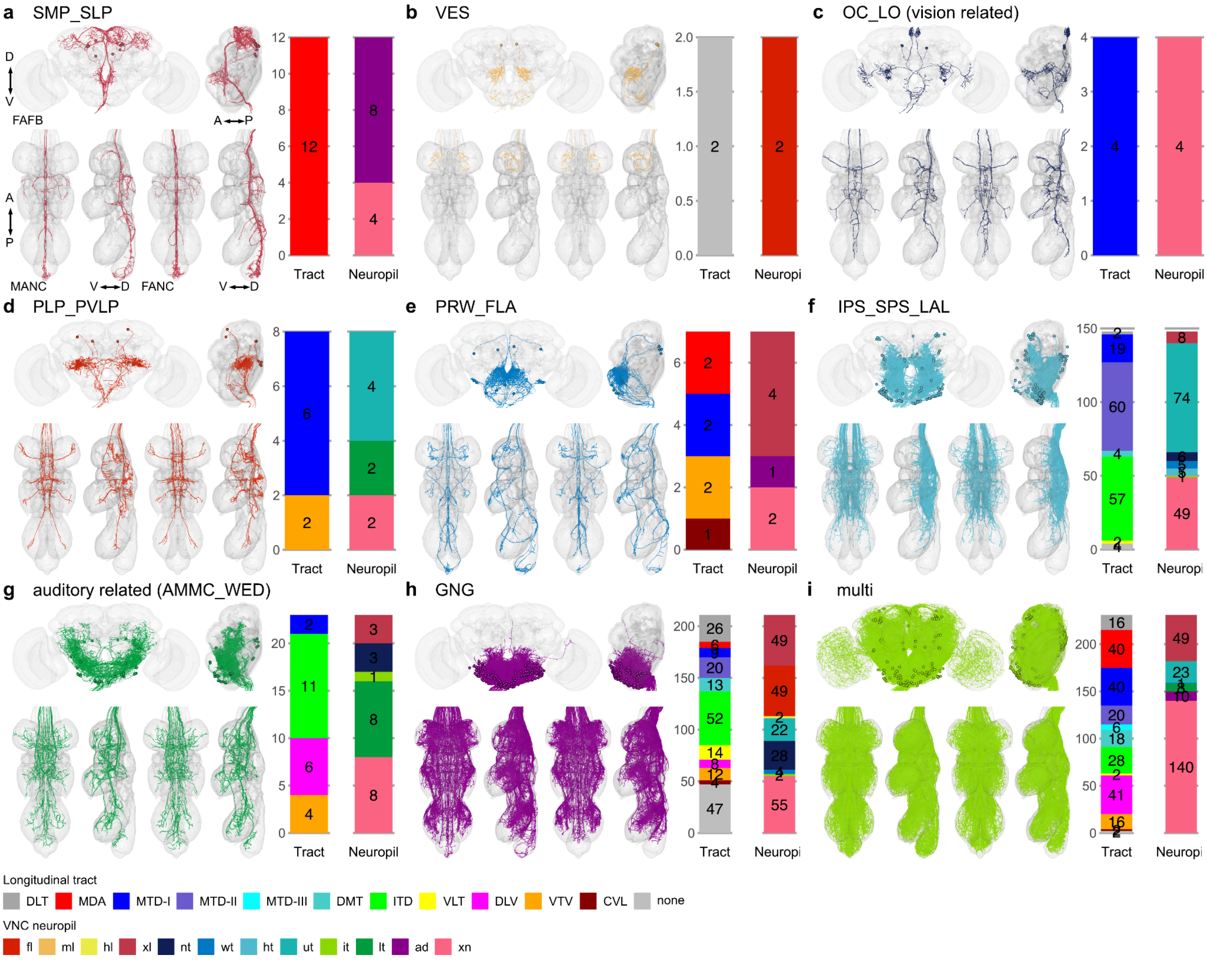

Fig.1 - figure supplemental 4: Morphology matched across the neck - brain neuropil. Morphology of LM matched DNs across the three datasets colour coded by their brain neuropil innervation. DNs with input neuropil. **a,** superior medial protocerebrum and superior lateral protocerebrum (SMP_SLP); **b**, vest (VES); **c**, ocellar ganglion and lobular (OC_LO, vision related); **d**, posterior lateral protocerebrum (PLP); **e**, prow and flange (PRW_FLA); **f**, posterior slope and lateral accessory lobe (IPS_SPS_LAL); **g**, antennal mechanosensory and motor center and wedge (AMMC_WED, auditory related); **h**, gnathal ganglia (GNG) and **i**, multiple innervations of neuropils across the brain (multi). In each panel the top images show reconstruction in FAFB in anterior and lateral view; the two bottom left images show MANC and two bottom right FANC in ventral and lateral view, respectively. The bar charts represent the distribution of the VNC characteristics longitudinal tract and neuropil innervation for the neurons in each category for which the colour legend can be found at the bottom.

bioRxiv preprint doi: https://doi.org/10.1101/2024.06.04.596633; this version posted June 6, 2024. The copyright holder for this preprint (which was not certified by peer review) is the author/funder, who has granted bioRxiv a license to display the preprint in perpetuity. It is made available under aCC-BY 4.0 International license.

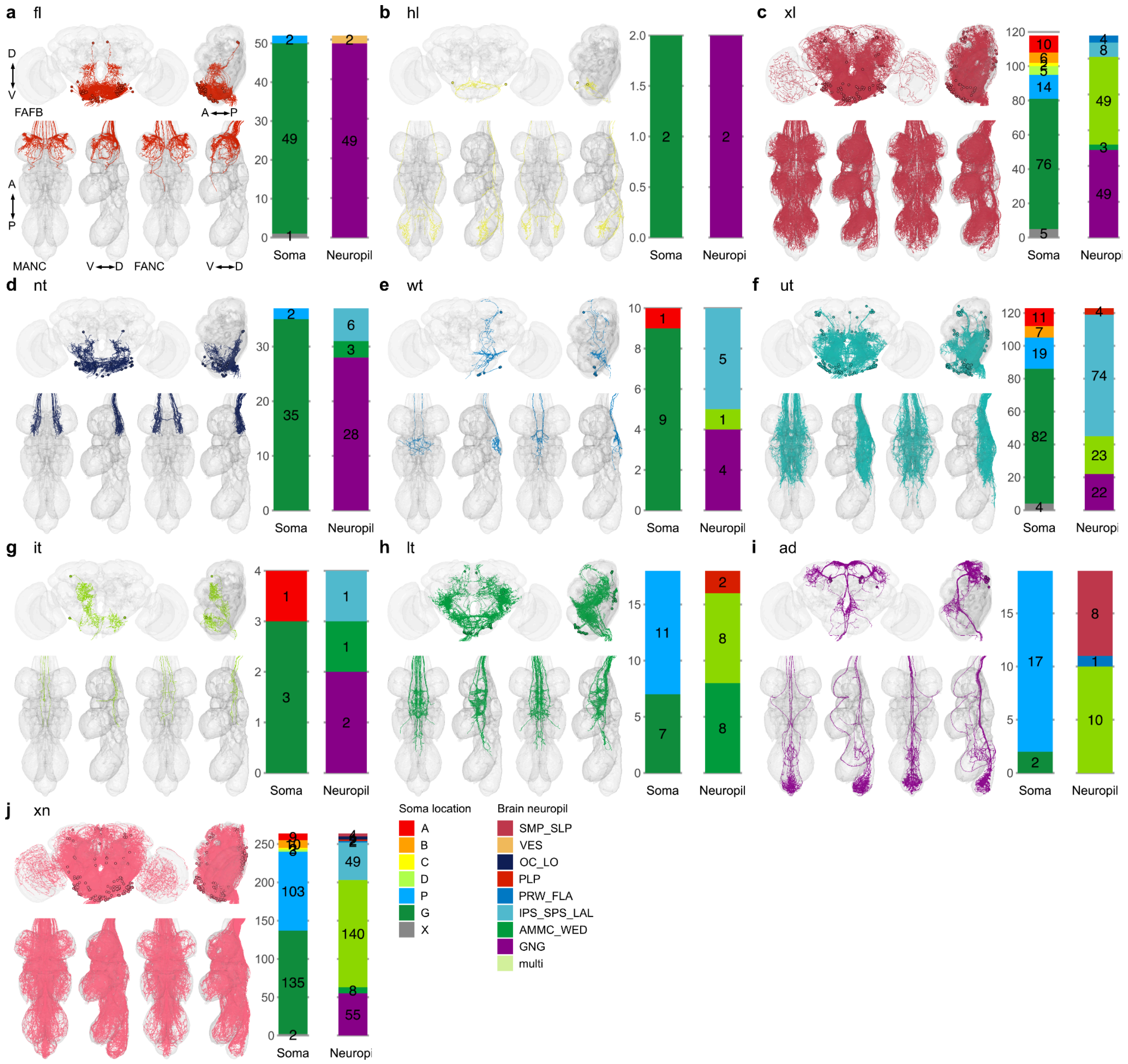

Fig.1 - figure supplemental 5: Morphology matched across the neck - VNC neuropil. Morphology of LM matched DNs across the three datasets colour coded by VNC neuropil innervation. DNs with output neuropil. **a,** front leg (fl); **b**, hind leg (hl); **c**, multiple innervation in leg compartments (xl); **d**, neck tectulum (nt); **e**, wing tectulum (wt); **f**, multiple innerveration into upper tectulum neuropils of the neck, wings and halteres (ut); **g**, intermediate tectulum (it); **h**, lower tectulum (lt); **i**, abdomen (ad) and **j**, multiple innervations of neuropils across the VNC (xn). In each panel the top images show reconstruction in FAFB in anterior and lateral view; the two bottom left images show MANC and two bottom right FANC in ventral and lateral view, respectively. The bar charts represent the distribution of the brain characteristics soma location and neuropil innervation for the neurons in each category for which the colour legend can be found at the bottom right.

bioRxiv preprint doi: https://doi.org/10.1101/2024.06.04.596633; this version posted June 6, 2024. The copyright holder for this preprint (which was not certified by peer review) is the author/funder, who has granted bioRxiv a license to display the preprint in perpetuity. It is made available under aCC-BY 4.0 International license.

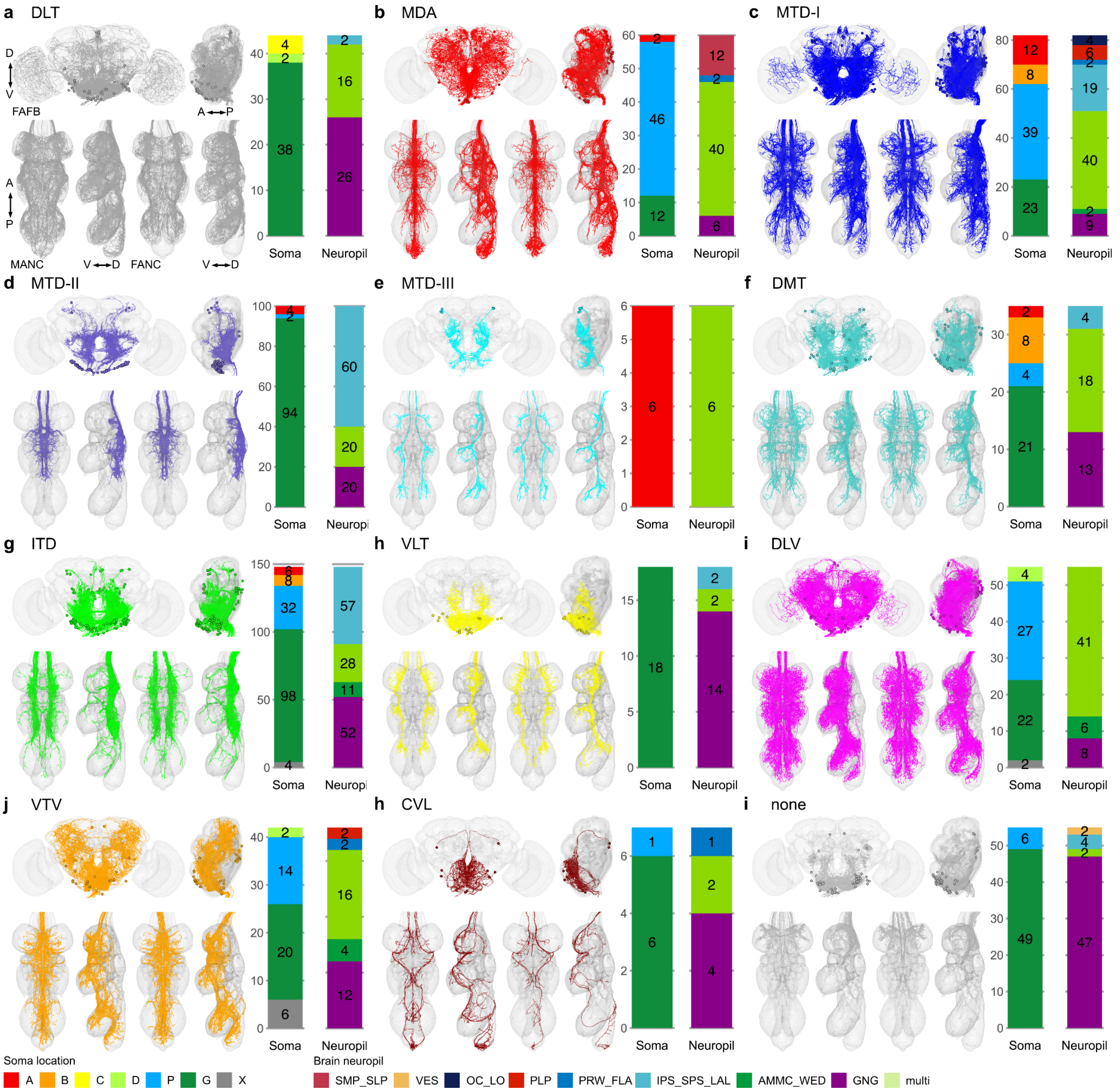

Fig.1 - figure supplemental 6: Morphology matched across the neck - tract. Morphology of LM matched DNs across the three datasets colour coded by longitudinal tract membership in the VNC. DNs in the tract. **a**, dorsal lateral tract (DLT); **b**, median dorsal abdominal tract (MDA); **c**, ventral route of the mediate tract of dorsal cervical fasciculus (MTD-I); **d**, dorsal route of the mediate tract of dorsal cervical fasciculus (MTD-II); **e**, lateral route of the mediate tract of dorsal cervical fasciculus (MTD-III); **f**, dorsal median tract (DMT); **g**, intermediate tract of dorsal cervical fasciculus (ITD); **h**, ventral lateral tract (VLT); **i**, dorsal lateral tract of ventral cervical fasciculus (DLV); **j**, ventral median tract of ventral cervical fasciculus (VTV); **h**, curved ventral lateral tract (CVL) and **i**, no tract membership (none). In each panel the top images show reconstruction in FAFB in anterior and lateral view; the two bottom left images show MANC and two bottom right FANC in ventral and lateral view, respectively. The bar charts represent the distribution of the brain characteristics soma location and neuropil innervation for the neurons in each category for which the colour legend can be found at the bottom.

bioRxiv preprint doi: https://doi.org/10.1101/2024.06.04.596633; this version posted June 6, 2024. The copyright holder for this preprint (which was not certified by peer review) is the author/funder, who has granted bioRxiv a license to display the preprint in perpetuity. It is made available under aCC-BY 4.0 International license.

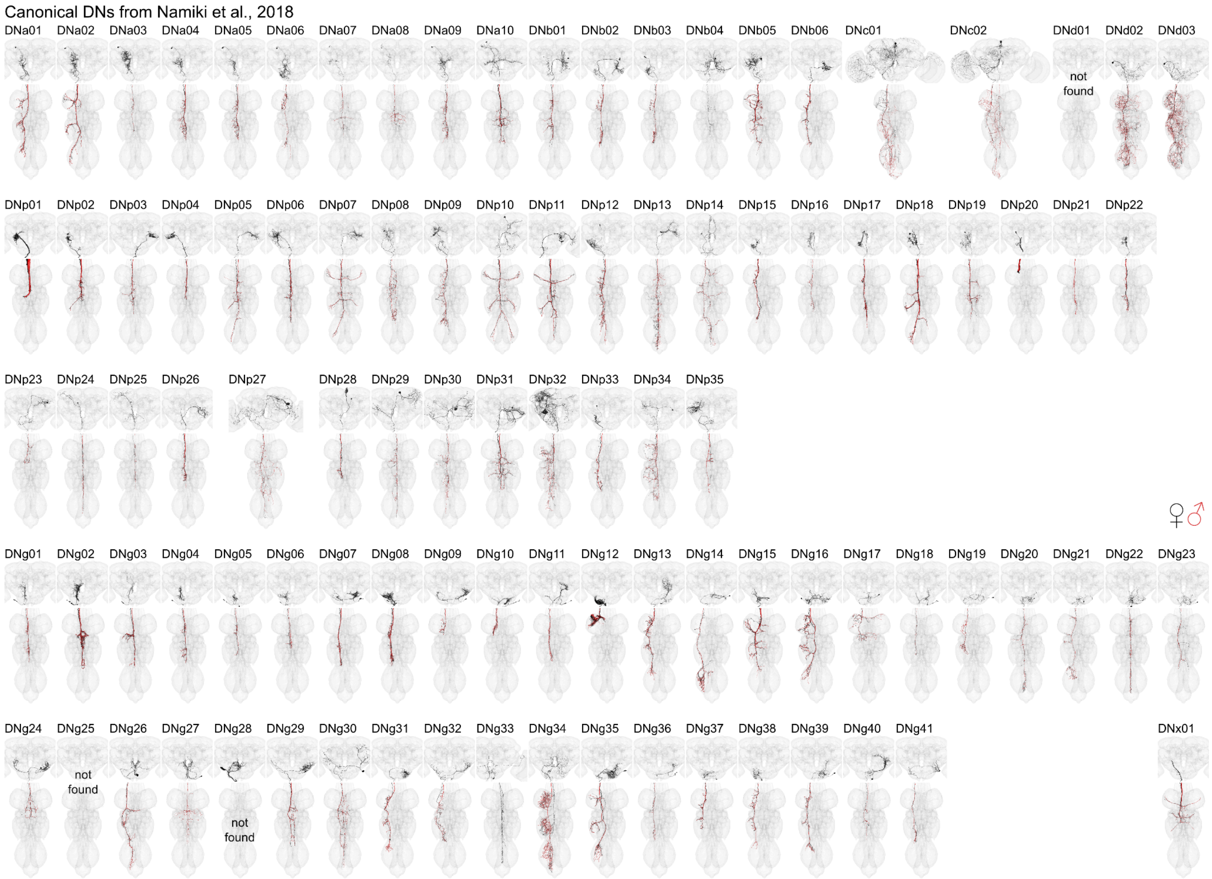

Fig.2 - figure supplemental 1: DN matching to Namiki et al. 2018. Morphology of identified DNs across all three datasets with nomenclature as described in (Namiki et al. 2018). Two DN types could not be identified (DNd01, DNg25) in any of the three EM datasets and one DN type (DNg28) is only identifiable in the brain. See supplementary table DN_identification for slide codes and for DN synonyms from the literature. In black the morphology of DNs in female datasets (FAFB, FANC) in red from the male dataset (MANC).

bioRxiv preprint doi: https://doi.org/10.1101/2024.06.04.596633; this version posted June 6, 2024. The copyright holder for this preprint (which was not certified by peer review) is the author/funder, who has granted bioRxiv a license to display the preprint in perpetuity. It is made available under aCC-BY 4.0 International license.

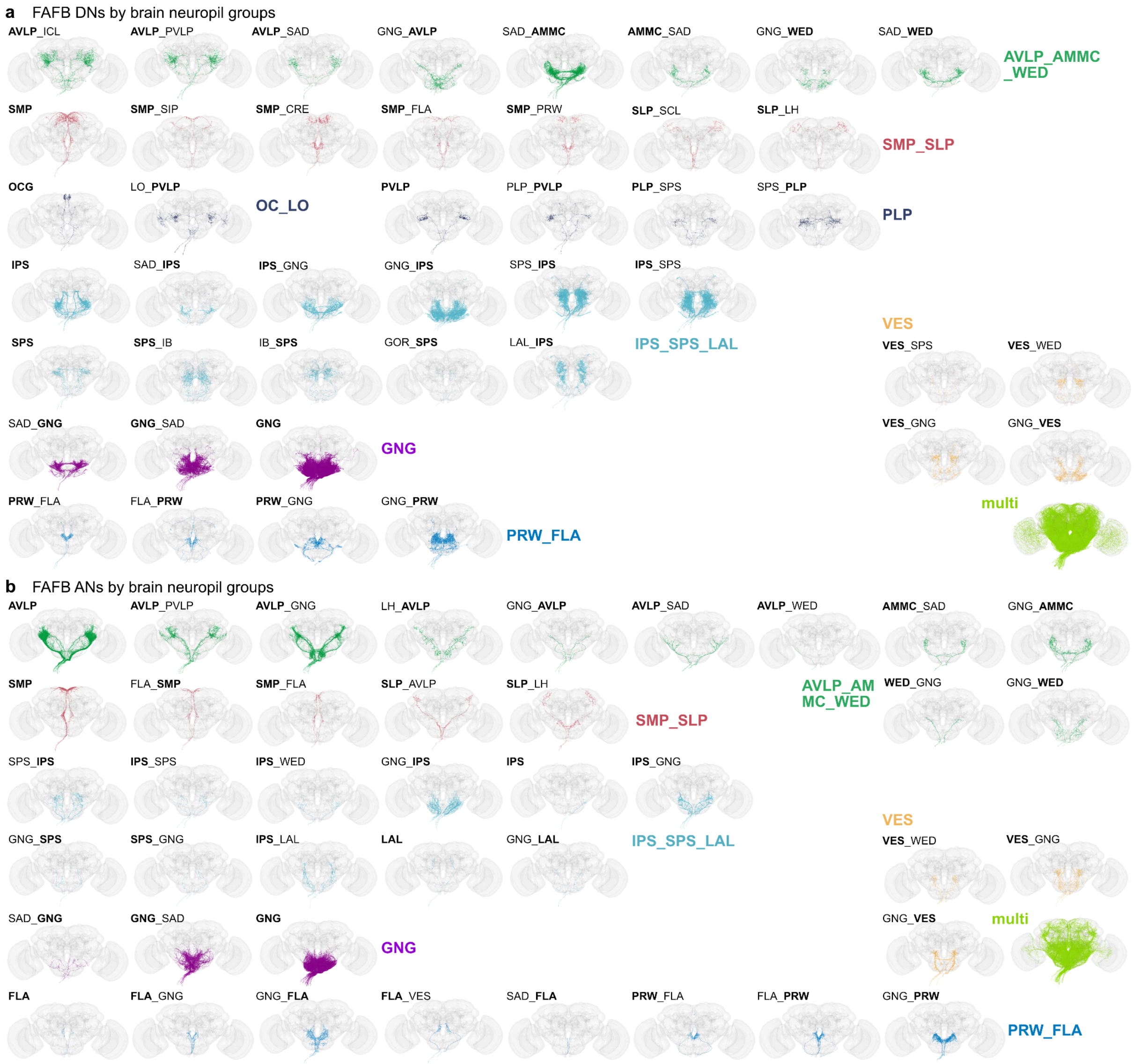

Fig.3 - figure supplemental 1: Brain neuropil groups. Morphology of DNs and ANs by primary brain neuropil. **a**, All DNs in FAFB were assigned one or two input neuropils. DNs that receive input from more than two neuropils are referred to as multi. **b**, All ANs in FAFB were assigned one or two output neuropils. ANs that output to more than two neuropils are referred to as multi. Morphologies are coloured by broader neuropil groups: auditory related neuropils (AVLP_AMMC_WED), higher order multimodal sensory integration 1 (SMP_SLP), vision related (OC_LO), multimodal sensory integration 2 (PVLP, PLP), sensory modalities from the GNG (GNG, SAD), multimodal sensory integration and steering (IPS_SPS_LAL), vest related (VES) and flange/prow (PRW_FLA).

bioRxiv preprint doi: https://doi.org/10.1101/2024.06.04.596633; this version posted June 6, 2024. The copyright holder for this preprint (which was not certified by peer review) is the author/funder, who has granted bioRxiv a license to display the preprint in perpetuity. It is made available under aCC-BY 4.0 International license.

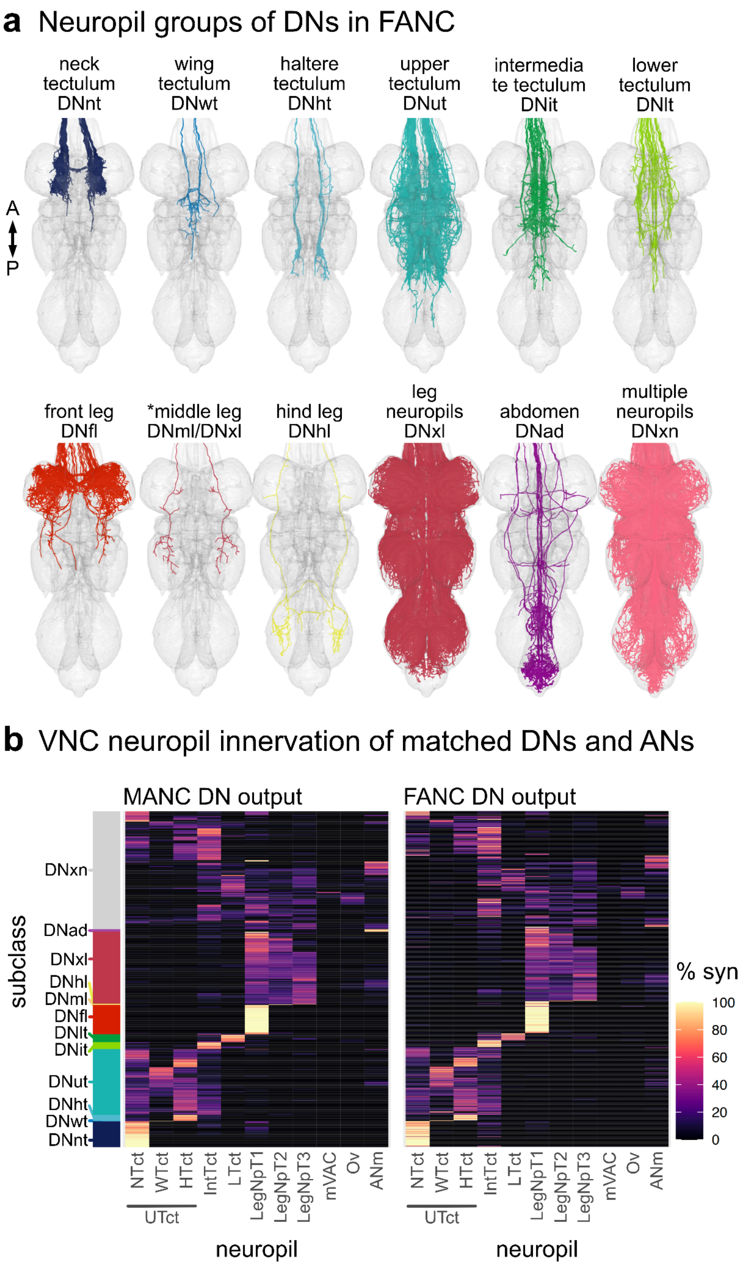

Fig.5 - figure supplemental 1: Neuropil-based analysis of descending neurons in FANC. a, Primary neuropil assignment of DNs in the FANC dataset to compare to previously published one in the MANC dataset (Cheong et al. 2023). **b**, Synaptic output in % by VNC neuropil of matched DNs in MANC and FANC. Each row represents one DN type, order is conserved between the two datasets. Left bar indicates the previously assigned neuropil based subclasses from the MANC dataset (Cheong et al. 2023).

bioRxiv preprint doi: https://doi.org/10.1101/2024.06.04.596633; this version posted June 6, 2024. The copyright holder for this preprint (which was not certified by peer review) is the author/funder, who has granted bioRxiv a license to display the preprint in perpetuity. It is made available under aCC-BY 4.0 International license.

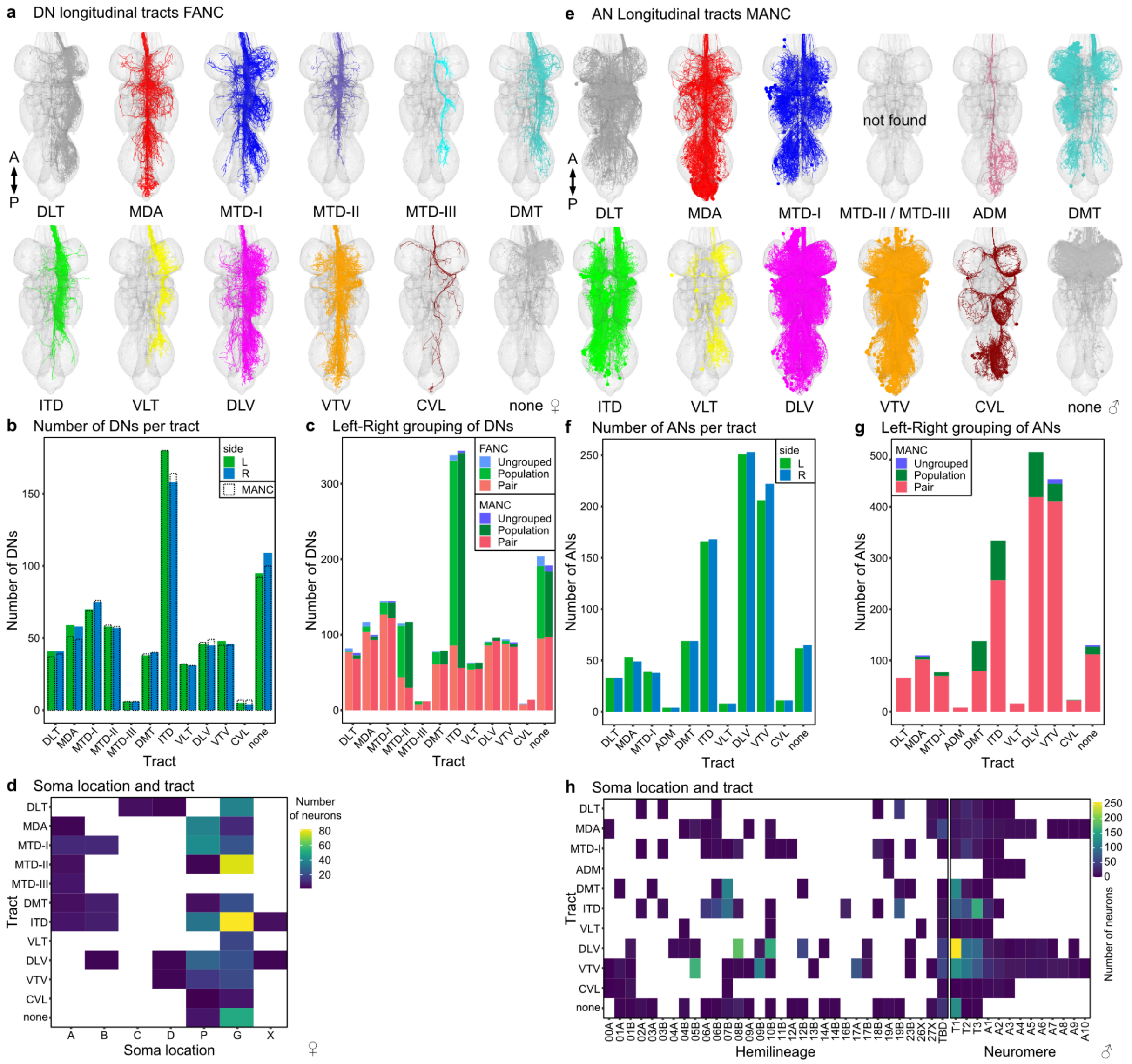

Fig.5 - figure supplemental 2: Tract-based analysis of descending neurons in FANC and ascending neurons in MANC. a, Tract assignment of all left side DNs in the FANC dataset to compare to previously published tract assignment in the MANC dataset (Cheong et al. 2023). **b**, Number of DNs for each tract in comparison to MANC DNs (dotted line). **c**, DNs grouped into pairs or populations comparing FANC to MANC. **d**, Correlation of soma location and tract membership for identified FANC DN types based on LM data (Namiki et al. 2018). **e**, Tract assignment of all left side ANs in the MANC dataset. None of the ANs project along the MTD-II or MTD-III tract. A small additional tract was observed for ANs, referred to as AN specific dorsal medial tract (ADM). **f**, Number of ANs in each tract. **g**, ANs grouped into pairs or populations comparing MANC to FANC. **h**, Correlation of hemilineage and neuromere to tract membership for MANC ANs (Marin et al. 2023).

bioRxiv preprint doi: https://doi.org/10.1101/2024.06.04.596633; this version posted June 6, 2024. The copyright holder for this preprint (which was not certified by peer review) is the author/funder, who has granted bioRxiv a license to display the preprint in perpetuity. It is made available under aCC-BY 4.0 International license.

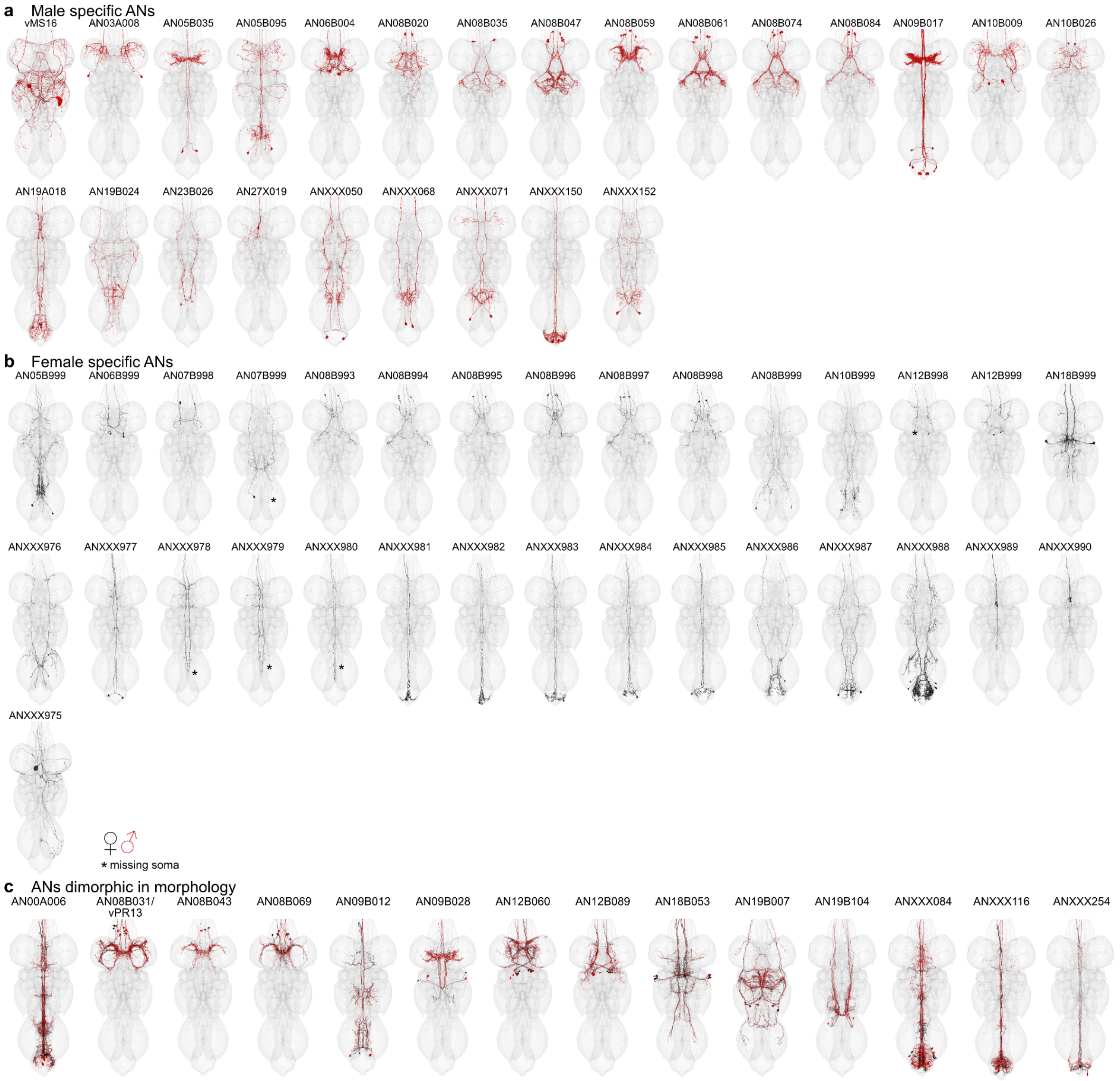

Fig.8 - figure supplemental 1: Potentially sexually dimorphic or sex-specific ANs in the VNC. a, Morphology of all the potentially male specific ANs by type. **b**, Morphology of the potentially female specific ANs by newly assigned types. **c**, Morphology of the potentially sex. dimorphic ANs by MANC type names. In black the EM morphology from the female dataset (FANC) in red from the male dataset (MANC). Stars indicate ANs with missing soma in FANC due to missing EM image data.

bioRxiv preprint doi: https://doi.org/10.1101/2024.06.04.596633; this version posted June 6, 2024. The copyright holder for this preprint (which was not certified by peer review) is the author/funder, who has granted bioRxiv a license to display the preprint in perpetuity. It is made available under aCC-BY 4.0 International license.

Supplemental information

Supplementary file 1 Seed planes

Two tables listing the xyz coordinates, svid, root_id, side and class of profiles passing through the FAFB or FANC seed plane.

Supplementary file 2 Typing and matching

13 tables listing the neuronal ids and annotations used in the manuscript for: FAFB_DNs, FANC_DNs, MANC_DNs, dimorphic_DNs, DN_identification, FAFB_ANs_SAs, FANC_ANs, FANC_SAs, MANC_ANs, dimorphic_ANs, AN_identification, FAFB_SA_identification, other_MANC_FANC_matching

Supplementary file 3 User edits

Two tables listing the number of edits to the neck connective neurons in FANC and FAFB summarised by lab.
